# Robust validation and performance comparison of immunogenicity assays assessing IgG and neutralizing antibodies to SARS-CoV-2

**DOI:** 10.1371/journal.pone.0262922

**Published:** 2022-02-07

**Authors:** Marie E. Bonhomme, Cyrille J. Bonhomme, Lisa Strelow, Atul Chaudhari, Adrienne Howlett, Carl Breidenbach, Jack Hester, Christopher Hammond, Michéal Fuzy, Laura Harvey, Vanessa Swanner, Jeymie Ellis, Rebecca R. Greway, Victoria A. Pisciella, Tina Green, Lisa Kierstead

**Affiliations:** 1 Laboratory Services, Vaccine Sciences Lab, PPD^®^, Part of Thermo Fisher Scientific, Richmond, Virginia, United States of America; 2 Department of Laboratory Biostatistics, PPD^®^, Part of Thermo Fisher Scientific, Richmond, Virginia, United States of America; National Institutes of Health, UNITED STATES

## Abstract

To enable benchmarking of immunogenicity between candidate severe acute respiratory syndrome coronavirus 2 (SARS-CoV-2) vaccines, there is a need for standardized, validated immunogenicity assays. In this article, we report the design and criteria used to validate immunogenicity assays and the outcome of the validation of serologic and functional assays for the evaluation of functional immune response and antibody titers in human serum. A quantitative cell-based microneutralization (MNT) assay, utilizing a reference standard, for detecting anti-SARS-CoV-2 spike protein-neutralizing antibodies in human serum and Meso Scale Discovery’s multiplex electrochemiluminescence (MSD ECL) assay for immunoglobulin G (IgG) antibodies to SARS-CoV-2 spike, nucleocapsid, and receptor-binding domain (RBD) proteins were assessed for precision, accuracy, dilutional linearity, selectivity, and specificity using pooled human serum from coronavirus disease 2019 (COVID-19)-confirmed recovered donors. Both assays met prespecified acceptance criteria for precision, relative accuracy, dilutional linearity, selectivity, and specificity. Both assays demonstrated high specificity for the different SARS-CoV-2 antigens or virus tested, and no significant cross-reactivity with seasonal coronaviruses. An evaluation to compare the neutralizing activity in the MNT assay to the IgG measured using the MSD ECL assay showed a strong correlation between the presence of neutralizing activity and amount of antibodies against the spike and RBD proteins in sera from both convalescent and vaccinated individuals. Finally, the MNT assay was calibrated to the WHO reference standard to enable reporting of results in international units, thus facilitating comparison of immunogenicity data generated by different assays and/or laboratories. The MSD ECL assay has previously been calibrated. In conclusion, these validated assays for the evaluation of functional immune response and antibody titers following SARS-CoV-2 vaccination could provide a relatively simple standardized approach for accurately comparing immune responses to different vaccines and/or vaccination regimens.

## Introduction

The emergence of severe acute respiratory syndrome coronavirus 2 (SARS-CoV-2) in late 2019, and the start of the subsequent coronavirus disease 2019 (COVID-19) pandemic [1], led to a rapid international vaccine development response [2, 3]. By the end of 2020, more than 170 potential SARS-CoV-2 vaccines were in preclinical development and more than 60 were undergoing clinical evaluation [2].

Both the World Health Organization (WHO) and the US Food and Drug Administration (FDA) have published considerations and guidance for the development and evaluation of SARS-CoV-2 vaccines [4, 5]. The guidance stipulates that preclinical studies and clinical trials of vaccines should include evaluation of humoral, cellular, and functional immune responses. Antigen-specific enzyme-linked immunosorbent assays (ELISAs) are recommended to characterize the humoral response, and functional activity should be evaluated with *in vitro* neutralization assays using either wild-type virus or pseudovirus microneutralization (MNT) [4, 5]. The WHO “Considerations for Evaluation of COVID-19 Vaccines” document comments that the serologic correlates of protection evaluated must be justified and supported by the best scientific evidence available and that there should be evidence of assay validation and standardization [4]. Similarly, the FDA Guidance for Industry “Development and Licensure of Vaccines to Prevent COVID-19” notes that assays utilized for the evaluation of immunogenicity should demonstrate suitability for their intended purposes and be validated before use in pivotal clinical trials [5].

However, current SARS-CoV-2 vaccine immunogenicity assays are limited by the lack of standardized methods for benchmarking serologic responses that would enable comparison between different candidate vaccines [6]. To ensure the accelerated development and availability of multiple vaccines globally, there is an urgent need for laboratory assays that would enable “bridging” or comparison of efficacy between candidate vaccines [7]. To address this need for standardized validated immunogenicity assays, we developed and validated two assays to be used in combination to evaluate the immunogenicity of SARS-CoV-2 vaccines using human serum samples. One assay is a cell-based MNT assay for detecting SARS-CoV-2 spike protein-neutralizing antibodies, and the other, from Meso Scale Discovery (MSD), a multiplex electrochemiluminescence (MSD ECL) assay for determination of Immunoglobulin G (IgG) antibodies to SARS-CoV-2 spike, nucleocapsid, and receptor-binding domain (RBD) proteins. In this article, we describe the design and criteria used to validate these immunogenicity assays and report the outcomes of the validation of these assays in terms of precision, accuracy, dilutional linearity, selectivity, and specificity, as well as a comparison of performance between the MNT and MSD ECL assays in human serum samples from COVID-19 convalescent and vaccinated individuals (mRNA vaccine mRNA-1273 [8] or BNT162b2), which were known to contain anti-SARS-CoV-2 antibodies. Consequently, both assays were validated and deemed acceptable for the assessment of functional immune response and antibody concentrations (Ab[C]) for immunogenicity testing during SARS-CoV-2 vaccine trials.

## Methods

### Validation criteria for the MNT and MSD ECL assays

Method validations are performed to ensure that the assay is accurate, specific, and reproducible over the specified range that a target is evaluated. Multiple parameters are assessed during validation, including precision, relative accuracy, dilutional linearity, selectivity, and specificity. The assessment of these different parameters allows the determination of the limits of quantification (LOQ) and limits of detection (LOD).

Precision experiments are designed to determine inter-, intra-, and total assay precision. Intra-assay variability represents within-run variation, whereas inter-assay variability represents the between-run variation (reproducibility) attributable to different days, analysts, reagents, etc. The intermediate assay precision is the sum of the intra- and inter-assay variability. The overall variability (percent geometric coefficient of variation [%GCV]) was expected to be < 40% GCV for the MNT assay and < 25% GCV for the MSD ECL assay.

Accuracy is the closeness of agreement between the value that is accepted, either as the conventional true value or an accepted reference value, and the value determined. It is typically evaluated by testing a range of known Ab[C]s to determine the range over which the assay is accurate. Accuracy was reported as percent recovery and calculated by dividing the measured concentration by the expected concentration. Accuracy estimates were expected to be within ± 1.30-fold (77% to 130%) of expected concentrations throughout the quantifiable range.

Selectivity is the ability of the assay to measure and differentiate the analyte in the presence of components that may be expected to be present in the sample. Selectivity is assessed by spiking different concentrations of the reference standard (RS) (or sample with known Ab[C]) into samples with known low or negative Ab[C]s. The overall recovery estimates by spike concentration were derived using a mixed model containing a dependent variable for the natural log (ln)- transformed recovery ratio estimates. The overall recovery estimate across all spike levels within the quantifiable range were expected to be between 66.7% and 150%.

Dilutability, or dilutional linearity, is an attribute of a biologic assay that demonstrates that a test sample can be diluted through a series, yielding equivalent dilution-corrected Ab[C]s across that series. The dilution bias was estimated by fitting a mixed model that included fixed terms for the sample effect and for the average dilution effect (slope) and a random term representing the variability in the dilution effect across the individual test samples. Dilution bias was expected to be within 0.5 to 2.0-fold for a 16-fold dilution for the assay to be considered acceptably dilutable.

Analytic specificity is the ability of an analytic method to determine only the component it purports to measure, or the extent to which the assay responds only to all subsets of a specified analyte and not to other substances present in the sample. Specificity is demonstrated in competition experiments using homologous and heterologous competitor antigens or viruses. Specificity was determined by comparing the Ab[C] of the serum in competition with homologous or heterologous competitors to the Ab[C] of a mock-treated sample. The assays were considered acceptably specific if ≥75% of the samples showed (i) ≥75% inhibition in competition with the homologous antigens and (ii) ≤25% inhibition in competition with a heterologous antigen.

The LOD of an assay is the lowest concentration that has a high probability of producing a response that can be distinguished from the background response (i.e., the response at zero concentration). Antibody-depleted human serum (ADHS) and/or known negative samples were spiked with different levels of known positive samples. The LOD for the validation of these assays was set as the concentration of spiked sample that provided a statistically significant increase in Ab[C] or signal in the unspiked sample. Significance was based on a *t*-distribution at the 5% significance level and determined using the mean and standard deviation of the individual differences. The assay LOD was expected to be below the lower limit of quantification (LLOQ).

The upper and lower limits of quantification (ULOQ and LLOQ) define the Ab[C] range over which the assay is accurate and precisely quantitates samples. The final LOQs (working range) were set based on the acceptable performance of the assay and determined by evaluating the precision profile of the Ab[C]s and the relative accuracy of the assay. The LLOQ must be greater than the assay LOD.

In addition to the parameters listed above, system suitability criteria including duplicate variability (extravariability), standard curve modeling and controls (including root mean square error, minimum and maximum slope and the concentration corresponding to the median signal response), blank Ab[C] limit, and quality control (QC) limits, were evaluated and/or confirmed.

### MNT assay methods

The SARS-CoV-2 MNT assay (SARS-CoV-2 MNT; PPD^®^, part of Thermo Fisher Scientific, Richmond, Virginia, USA) was designed to determine the ability of SARS-CoV-2 spike neutralizing antibodies to inhibit the infection of 293T-angiotensin-converting enzyme 2 (ACE2) cells by SARS-CoV-2 spike D614G variant [9] reporter virus particles (RVP) that express green fluorescent protein (GFP).

In this assay, serum samples were pre-incubated with a known quantity of RVP (Integral Molecular Inc., Philadelphia, Pennsylvania, USA) for 60 (± 5) minutes prior to addition to 293T-ACE2 cells (Integral Molecular Inc.) in 96-well plates. Infection of the 293T-ACE2 cells by RVP was enumerated 48 (± 4) hours after exposure by counting the number of green fluorescent cells (i.e., foci-forming units [FFU]) using a Cytation™ 5-cell imaging reader (BioTek^®^ Instruments Inc., Winooski, Vermont, USA). Each sample was tested in duplicate.

In routine operation of the assay, a RS (human serum from COVID-19-confirmed convalescent donors [Lot V62RS-X132-CVMN; BioIVT LLC, Westbury, New York, USA]), was included in the assay and assigned an arbitrary concentration of 1,000 arbitrary units (AU)/ml. A ten-point, two-fold dilution series of the RS, starting at a dilution of 1:50, was performed in duplicate on each assay plate and the culmination of all dilution points referred to as the standard curve. A four-parameter logistic function was used to model the standard curve dilution series for each plate. In addition, each plate included two blank negative control wells (cell culture media only), four positive virus control wells (culture media containing SARS-CoV-2-GFP at a 1:50 final dilution), and three QC samples tested in duplicate. The QC samples comprised low and high Ab[C] QCs (LQC and HQC, respectively) human sera with low and high anti-SARS-CoV-2 Ab[C], respectively, (BioIVT LLC) and mid Ab[C] QC (MQC) human sera with mid-range anti-SARS-CoV-2 Ab[C] (Antibody Systems, Inc., Hurst, Texas, USA) tested at final dilutions of 1:50 (LQC), 1:200 (MQC), and 1:800 (HQC).

Each sample was tested in a four-point, four-fold dilution series from 1:50 to 1:3,200. The serum Ab[C] was determined by interpolating the mean of the duplicate FFU values off the fitted RS curve. The interpolated Ab[C]s were then dilution-corrected and the final sample Ab[C] was therefore the Ab[C] associated with the lowest dilution with an Ab[C] within the assay LOQ. If the sample Ab[C] was greater than the ULOQ at all dilutions, the sample was tested at a pre-dilution of 1:16 in the assay.

For validation experiments, up to eight experimental samples, each at in-well dilutions of 1:50, 1:200, 1:800, and 1:3,200 in duplicate, were included on each plate. Neutralizing antibody results were expressed in AU/ml as per the curve generated from the RS dilution series.

In addition, the RS lot V62RSX132-CVMN was calibrated against the WHO international standard for anti-SARS-CoV-2 immunoglobulin (referred as WHO RS hereafter, National Institute for Biological Standards and Control [NIBSC] international RS; NIBSC code 20/136), with an objective to determine the concentration assignment in international units per millilitre (IU/ml) and conversion factor from AU/ml to IU/ml. For this purpose, ten assay runs were performed across 25 days with each plate containing two independent preparations of the V62RSX132-CVMN and two independent preparations of the WHO RS. Both the V62RSX132-CVMN and WHO RS were analyzed in a 2-fold dilution series starting at 1:50 and ending at 1:25,600. Fold-bias for Ab[C]s of QCs was further evaluated after applying the conversion factor.

### MSD ECL assay methods

The quantitative MSD ECL assay was designed to detect antibodies to three different SARS-CoV-2 proteins in human serum, with Ab[C]s then determined by indirect binding to an ECL reagent. The MSD ECL assay kit was utilized as per the manufacturers’ instructions, except in circumstances noted below.

Ab[C]s were determined in an indirect binding format. Specifically, RS (standard curve), QC, and test samples were incubated on an MSD 96-well, 10-Spot Custom SARS-CoV-2 3-PLEX plate coated with SARS-CoV-2 spike, nucleocapsid, and RBD antigens. Any anti-SARS-CoV-2 antibodies present in the test sample bind to the plates and form an antibody–antigen complex. Following incubation with SULFO-TAG-labeled anti-human IgG total antibodies, ECL from bound SULFO-TAG-labeled antibodies was measured in relative light units (RLU) using the MSD SECTOR S600 Plate Reader. Ab[C]s in the test samples were then determined by interpolating their RLU values from the standard curve generated from serially diluted RS samples.

In routine operation of the assay, a RS, four QC samples, and a blank control sample in two wells were analyzed on each plate within each run to ensure the quality of the test sample results for that plate. The RS (Lot A00V0004), and three QC samples (Lots A00C0731, A00C0732, and A00C0733), were provided by MSD. The standard curve was based on a dilution series in MSD assay diluent (Diluent 100) starting with a 1:10 dilution (point 1) followed by a 2.5-fold serial dilution for a total of 10 points, modeled using a weighted 4-parameter logistic regression function. The RS was arbitrarily assigned a concentration of 700 AU/ml for spike protein, 800 AU/ml for nucleocapsid, and 300 AU/ml for RBD antigens. The three QC samples provided by MSD and a fourth QC sample, consisting of a COVID-19 convalescent patient serum sample (Sanguine BioSciences, Sherman Oaks, CA, USA), were tested at a final in-well dilution of 1:5,000. Sample blanks (MSD Diluent 100 only) were tested in duplicate on each plate. Each plate included up to 32 experimental samples in duplicate, at an in-well final dilution of 1:500, 1:5,000, or 1:50,000.

### MNT assay validation

#### Precision, LOQ, and LOD

Inter-, intra-, and total assay precision were assessed via the responses of 23 human serum samples, all of which had been pre-screened to span the anticipated range of the assay. An addition sample, known to be negative (ADHS) was utilized to evaluate the LOD. All samples were analyzed across 18 assay runs performed by six analysts. Each analyst performed at least three runs, comprising three plates per run across separate days; the runs were performed across at least 2 weeks.

#### Selectivity

To establish whether the assay could measure a known concentration of neutralizing antibody within negative or positive samples with known low Ab[C]s, one analyst performed one assay run of three plates. The negative and low Ab[C] samples were analyzed neat and also spiked with various dilutions of the RS.

#### Relative accuracy

At least one assay run was performed by one analyst. The run consisted of eight plates on which serum samples were analyzed at the standard assay dilutions (1:50 through 1:3,200). To assess the range over which the assay was accurate, an 8-point, 2-fold dilution series of the RS in cell culture medium, resulting in expected Ab[C]s of 1,000 (neat) to 7.8125 AU/ml, was prepared on the day of the assay. These preparations were treated as test samples and run in duplicate eight times within the assay and were analyzed independently as eight separate curves (one per plate).

#### Dilutional linearity

Seven neat samples were evaluated at final in-well dilutions of 1:50 to 1:3,200 and seven 1:16 pre-diluted samples at final in-well dilutions of 1:800 to 1:51,200 in one assay run, comprising two plates, performed by one analyst.

#### Specificity

Assay specificity was tested using a depletion method. Magnetic beads (Dynabeads^®^ Antibody Coupling Kit, Thermo Fisher Scientific, Waltham, Massachusetts, USA) were coupled with homologous or one of two heterologous competitor proteins by overnight incubation with 20 μg protein per mg of beads followed by washing in kit storage buffer. The homologous competitor was the SARS-CoV-2 spike protein (S1 + S2 ECD His tag; Sino Biological). The heterologous competitor proteins were influenza A/Hong Kong/45/2019 (H3N2) neuraminidase His-Tag and human coronavirus OC43 spike glycoprotein (full-length) sheep Fc-Tag (HEK293) (both heterologous antigens from LGC Ltd, Teddington, UK). Mock samples, with beads incubated with water and phosphate-buffered saline, were used as the comparator. For antibody depletion, four serum samples were incubated separately with each type of antigen-coupled beads and mock beads for 2 hours. Following removal of the coupled beads, depleted and non-depleted serum samples were tested together on the same plate at the primary sample dilution series.

### MSD ECL assay validation

#### Precision, relative accuracy, dilutional linearity, and LOQ

To investigate assay precision (inter-, intra-, and total assay precision), and plate lot, dilutional linearity, and LOQ, 21 samples (RP1–RP21; Antibody Systems, Inc., Hurst, Texas, USA), pre-screened to have Ab[C]s that span the quantifiable assay range, were analyzed across 15 runs performed by five analysts utilizing two different plate lots. Four quality control samples (QC1–QC4) were tested on each plate and were used to evaluate intra-assay precision.

Samples for the determination of assay relative accuracy were prepared from a human serum sample that had high Ab[C]s against all three of the SARS-CoV-2 antigens being tested, to ensure the entire concentration range of the assay was covered. The sample was tested as an 11-point, 2.5-fold dilution series from pre-dilution of 1 (neat) to a pre-dilution of 1:9,536.743 in MSD Diluent 100 across 15 runs performed by five analysts.

#### LOD and selectivity

LOD and selectivity were evaluated in two assay runs performed by two analysts using ADHS (tested at 1:500) and serum samples (tested at 1:5,000) with known low or negative Ab[C], respectively. ADHS and serum samples were analyzed neat and when spiked with various dilutions of the same serum sample used for the relative accuracy evaluation, and which had demonstrated high Ab[C]s across all three of the SARS-CoV-2 antigens.

#### Specificity

To evaluate assay specificity, eight serum samples identified as representing the mid-to-high range of Ab[C] quantifiable by the assay standard curve were divided into seven aliquots. The first two aliquots were analyzed without competition (water and MSD Diluent 100, respectively). Three aliquots were analyzed in competition with homologous antigens, SARS-CoV-2 N, SARS-CoV-2 S, and SARS-CoV-2 RBD (Meso Scale Diagnostics LLC, Rockville, Maryland, USA) at final concentrations of 10 μg/ml. As significant cross-reactivity was expected between the anti-spike and RBD antibodies and the RBD and spike antigens, respectively, these latter aliquots also enabled the evaluation of heterologous inhibition of antibodies as a result of such cross-reactions. Finally, two aliquots were analyzed in competition with a heterologous seasonal human coronavirus antigen (OC43 S, Sino Biological, Wayne, Pennsylvania, USA) also at 10 μg/ml. Two assay runs were performed by two analysts, with each run consisting of two plates. Each sample was tested at a dilution of 1:5,000.

### Comparison of assay performance

The correlation between the neutralizing Ab[C] generated in the MNT assay and the Ab[C] generated in the MSD ECL assay were evaluated using pair-wise comparisons of results from human sera from convalescent patients and vaccinated individuals. A panel of 32 independent samples from convalescent patients with known Ab[C] across the range of the assays were tested. In addition, a panel of 52 samples from individuals who received one or two doses of mRNA vaccine (mRNA-1273 [Moderna Inc., Cambridge, Massachusetts, USA] or BNT162b2 [Pfizer Inc. New York, New York, USA]), and 30 pairs of serum samples (Days 1 and 28 post-vaccination) from individuals vaccinated in a Moderna clinical trial (mRNA-1273-P201; NCT identifier: NCT04405076 [8]) were also tested.

### Data analysis and statistical methods

Values were calculated as means, least-squares means, or geometric means, with variability measured in the form of 90% or 95% CI, as appropriate, and %GCV, which was calculated as $\left(100\times \left(e^{\sqrt{{\hat{\sigma }}^{2}}}-1\right)\right)$ [10].

Statistical analyses were performed using SAS^®^ software (SAS, Carey, North Carolina, USA). In general, analyses were performed on ln-transformed data, which is a standard approach to evaluate any biological data. Variability estimates, measured in %GCV, were determined using the mixed procedure in the SAS^®^ software. Samples and/or plates with technical errors were excluded from the analyses.

#### Standard curves

Standard curve fitting was performed using SAS^®^ software. Plates were excluded from the data analysis if more than two standard curve points per plate were found to be invalid or if the root mean square error estimate, a measure of how closely the predicted values fit the observed values that was determined for each plate, exceeded pre-defined limits.

## Results

### MNT assay validation

The incorporation of a RS, developed in-house from a pool of convalescent sera, into the MNT assay allowed it to become a fully quantitative assay to improve overall precision over the standard IC_50_ assay.

#### Precision, relative accuracy, LOQ, and LOD

The ULOQ and LLOQ were set to ensure samples would be interpolated off the linear range of the standard curve. The non-dilution corrected LLOQ was 1.0 AU/ml and the ULOQ was 5.5 AU/ml. Dilution-corrected LOQs are obtained by multiplying each value by the dilution at which the sample was tested; the LLOQ and ULOQ associated with the primary dilutions in the assays were 50 AU/ml and 17,600 AU/ml, respectively.

**Fig 1:**
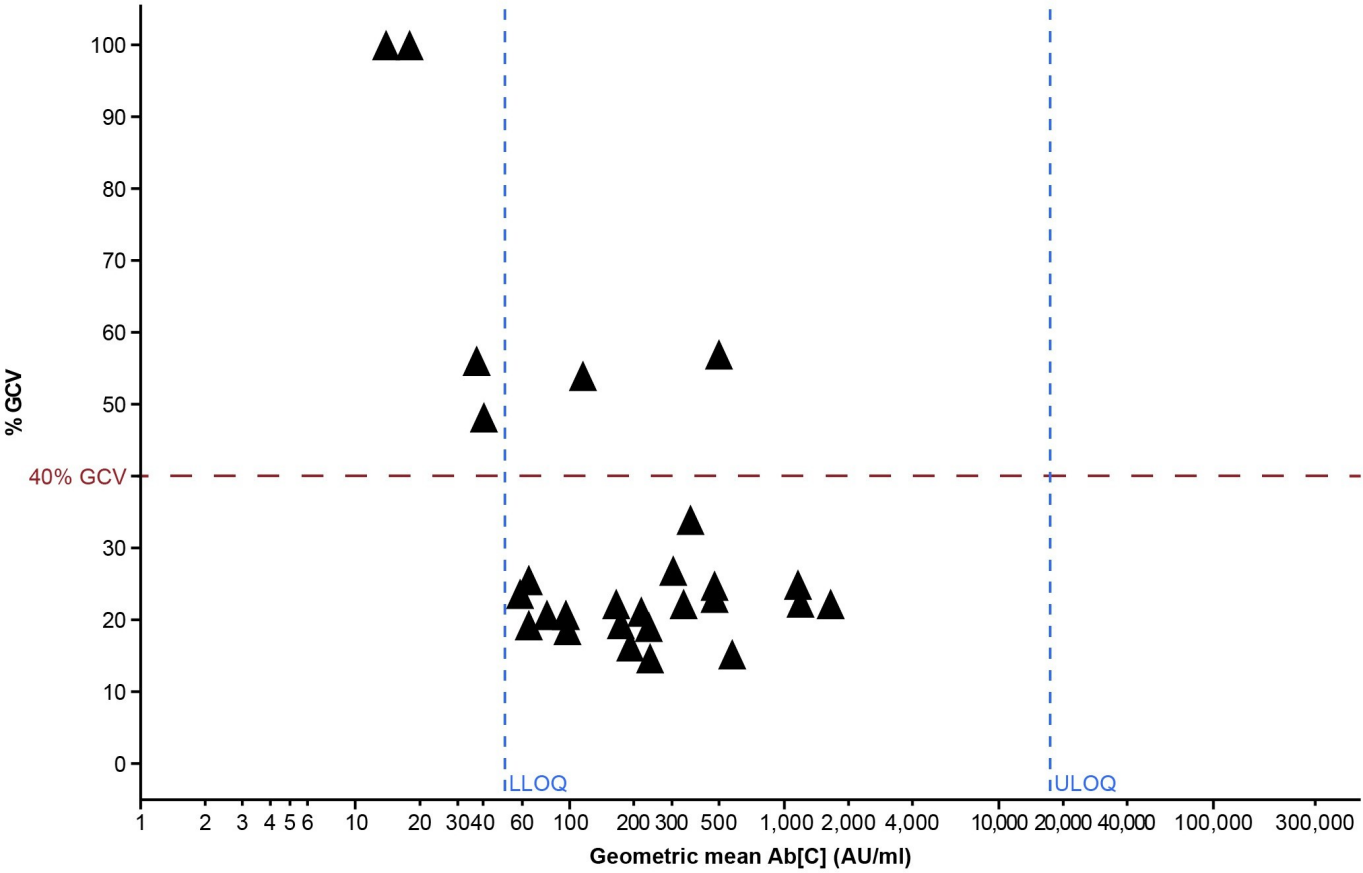
Anti-SARS-CoV-2-neutralizing antibody MNT assay precision. Precision profiles are shown across increasing geometric mean Ab[C]s (across 18 assay runs); precision profiles for QC samples are also shown. The horizontal red line shows the pre-defined %GCV criterion of < 40%. Vertical blue dashed lines represent the LOQs; %GCV values that exceed the Y-axis maximum value are displayed as 100% GCV. Ab[C] = antibody concentration; AU = arbitrary units; %GCV = percent geometric coefficient of variation; LLOQ = lower limit of quantitation; QC = quality control; SARS-CoV-2 = severe acute respiratory syndrome coronavirus 2; ULOQ = upper limit of quantitation.

**Fig 2:**
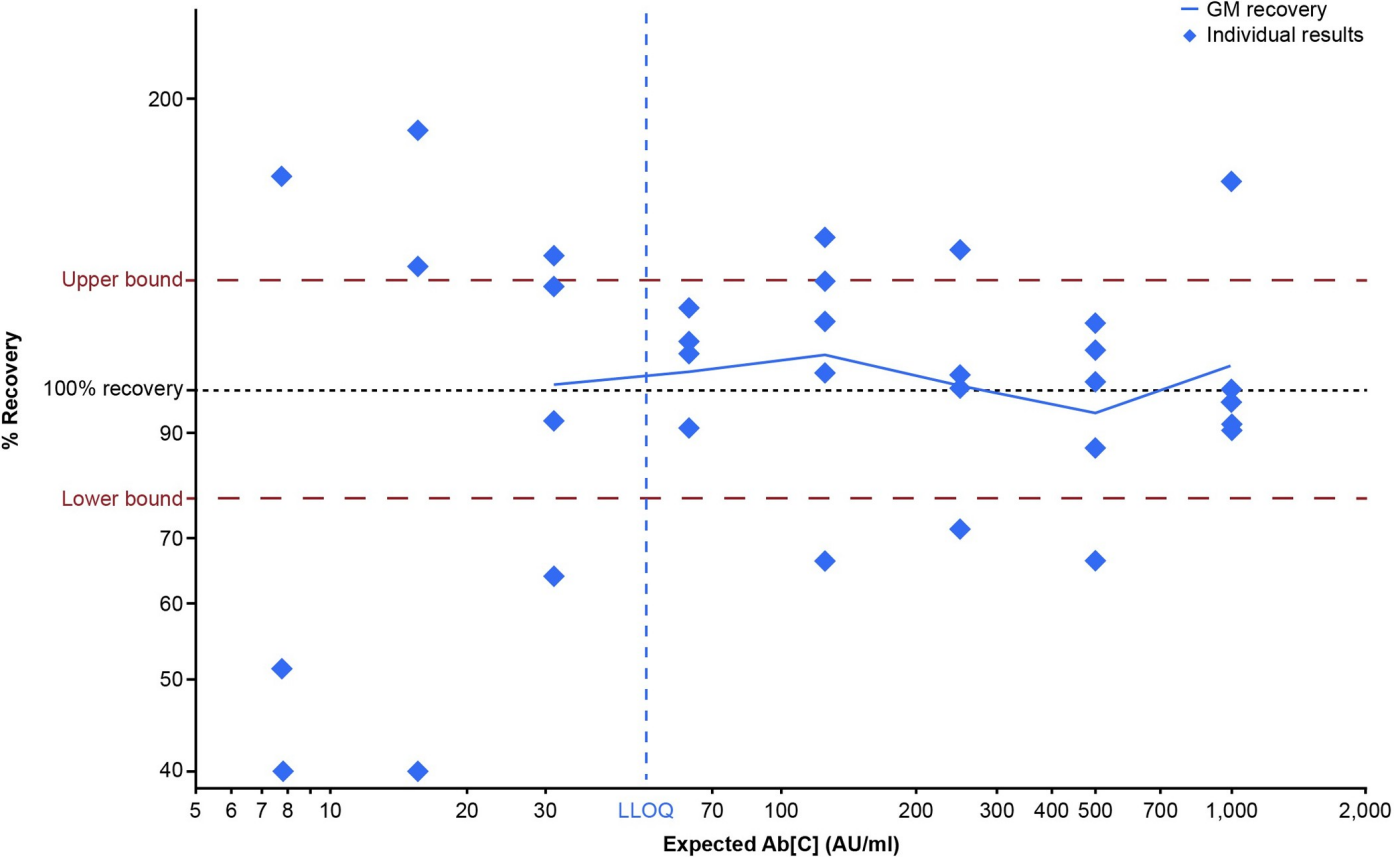
Anti-SARS-CoV-2-neutralizing antibody MNT assay relative accuracy. Overall and individual percentage recovery estimates from eight assay plates in a two-fold dilution series with expected concentrations from 30 to 1,000 AU/ml. The horizontal red dashed lines represent the pre-defined criteria of 77% to 130% for assay accuracy. Ab[C] = antibody concentration; AU = arbitrary units; GM = geometric mean; LLOQ = lower limit of quantitation; SARS-CoV-2 = severe acute respiratory syndrome coronavirus 2.

After application of the LOQs, 91.3% of the samples had variability estimates (%GCV) of < 40% (Fig 1) and the overall assay variability (%GCV) was 25.3%. It is of note that some samples had %GCV above 40% within the quantifiable range, which was representative of the sample-to-sample variability expected for functional assays. The percent recovery estimates (relative accuracy) for all samples within the LOQs were between 95% and 109% (Fig 2). Therefore, the assay was considered precise and accurate.

The LOD was set as the inverse-transformation of the 95% upper limit for multiple measurements (n = 18) of an ADHS sample. The resulting LOD was 43 AU/ml, which was lower than the LLOQ associated with the first dilution of the assay (50 AU/ml), as were all ADHS Ab[C] values.

#### Selectivity

Percent recovery estimates across concentration levels within the LOQs were 98% and 116% for the samples evaluated, a combined value of 107% recovery (90% confidence interval [CI]: 86, 132). This value met the acceptance criteria for selectivity–an overall percent recovery for concentrations within the LOQs between 66.7% and 150%.

#### Dilutional linearity

The assay showed dilutional linearity, with a dilution fold-bias estimate of 0.73-fold (95% CI: 0.42, 1.25) between neat samples and 1:16 pre-diluted samples. This met the acceptance criteria for dilutional linearity–a bias per 16-fold dilution between 0.5-fold and 2.0-fold.

#### Specificity

The assay met the pre-defined criteria of homologous inhibition ≥75% (89.6% for the SARS-CoV-2 spike protein) and heterologous inhibition ≤25% (2.9% for an influenza A neuraminidase protein and −31.3% for the human heterologous seasonal coronavirus OC43 spike protein). Overall specificity estimates are shown in S1 Fig.

### MSD ECL assay validation

The MSD ECL assay, based on an assay kit developed by Meso Scale Diagnostics LLC (Rockville, Maryland, USA), enables fully quantitative detection of IgG antibodies to three different SARS-CoV-2 proteins (spike, nucleocapsid, and RBD) in human serum.

#### Precision, relative accuracy, LOQ, and LOD

The LOQs were set based on acceptable performance of the assay, subject to being within the concentrations corresponding to the second lowest and second highest standard curve points and not less than the LOD. The assay LLOQs at the primary 1:5,000 dilution were 230, 270, and 190 AU/ml for the spike, nucleocapsid, and RBD antigens, respectively; the ULOQs at the primary 1:5,000 dilution were 140,000, 160,000, and 60,000 AU/ml, respectively.

**Fig 3:**
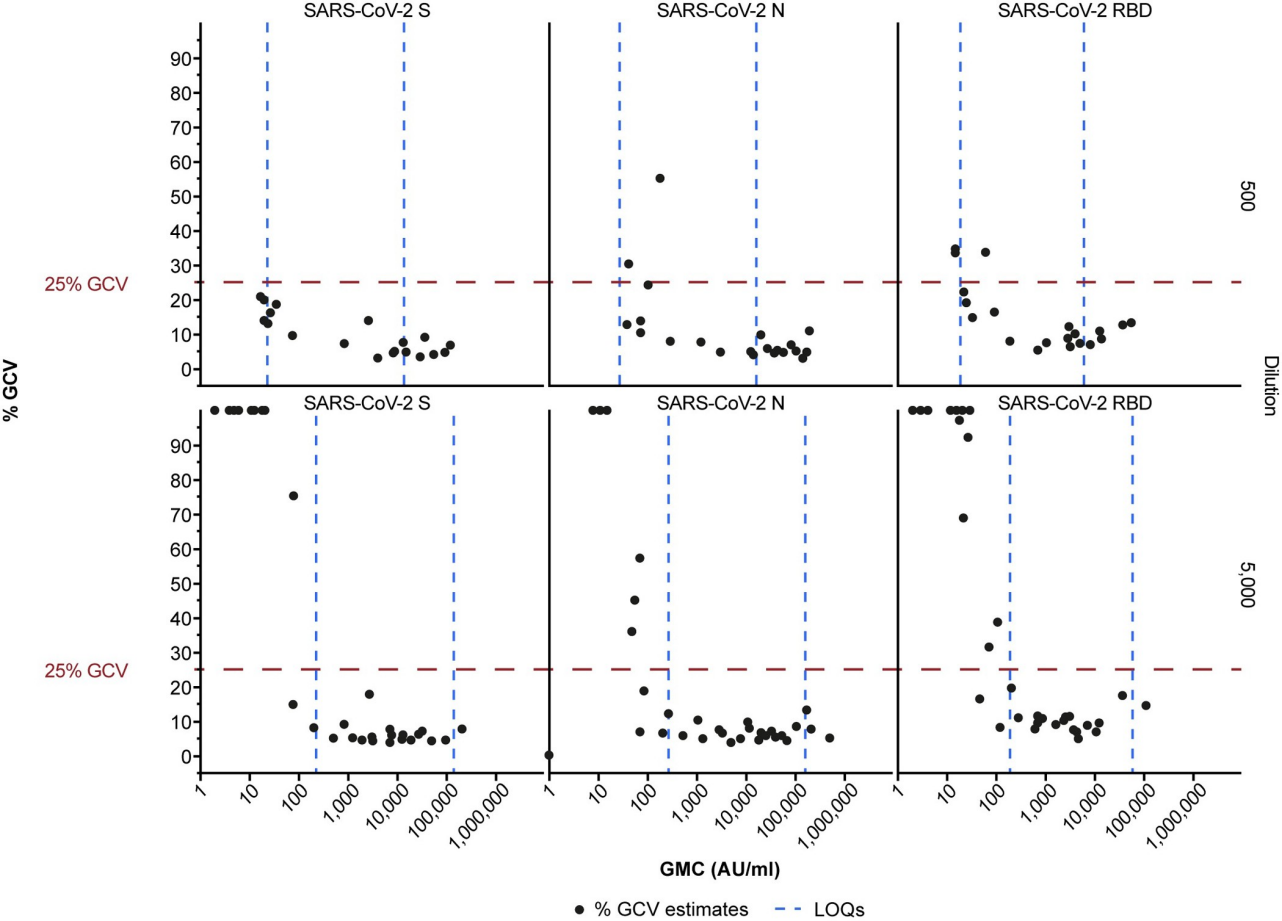
Anti-SARS-CoV-2 antibody MSD ECL assay precision. Precision profiles are shown for each of the three antigens in the assay, SARS-CoV-2 spike (SARS-CoV-2 S), nucleocapsid (SARS-CoV-2 N), and receptor-binding domain (SARS-CoV-2 RBD) across increasing geometric mean Ab[C]s (across 15 runs); precision profiles for quality control samples are also shown. The horizontal red dashed line represents the pre-defined %GCV criterion of ≤25%. GMC values that exceeded the X-axis are displayed as the minimum or maximum value. %GCV values that exceeded the Y-axis maximum value are displayed at the top of the graph. The LLOQ and ULOQ are shown by vertical dashed lines. AU = arbitrary units; GMC = geometric mean concentration; %GCV = percent geometric coefficient of variation; LLOQ = lower limit of quantitation; LOQ = limit of quantitation; SARS-CoV-2 = severe acute respiratory syndrome coronavirus 2; ULOQ = upper limit of quantitation.

**Fig 4:**
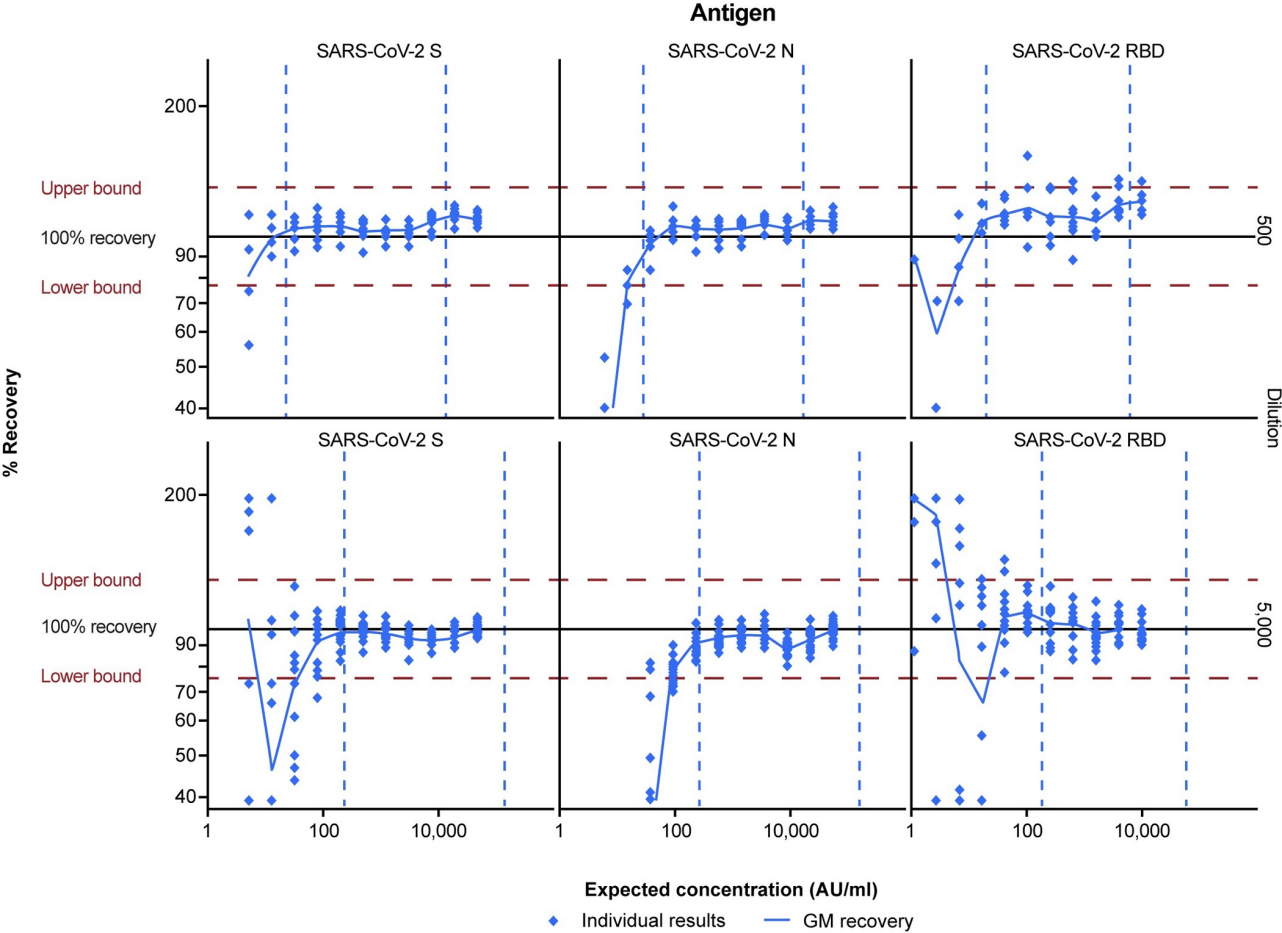
Anti-SARS-CoV-2 antibody MSD ECL assay relative accuracy. Overall and individual percentage recovery estimates (across 15 runs) are shown for each of the three antigens in the assay, SARS-CoV-2 spike (SARS-CoV-2 S), nucleocapsid (SARS-CoV-2 N), and receptor-binding domain (SARS-CoV-2 RBD), in a two-fold dilution series with expected concentrations from 51,171 to 5.4 AU/ml, 54,748 to 5.7 AU/ml, and 10,802 to 1.1 AU/ml, for SARS-CoV-2 S, SARS-CoV-2 N, and SARS-CoV-2 RBD, respectively. The horizontal red dashed lines represent the pre-defined criteria for assay accuracy of 77% to 130%. Vertical dashed lines represent the LLOQ and ULOQ. AU = arbitrary units; GM = geometric mean; LLOQ = lower limit of quantitation; SARS-CoV-2 = severe acute respiratory syndrome coronavirus 2; ULOQ = upper limit of quantitation.

After application of the LOQs at the primary 1:5,000 dilution, 100% of samples for the spike, nucleocapsid, and RBD antigens had %GCVs ≤25% (Fig 3). Therefore, assay precision was within the pre-defined acceptability parameters: ≥80% of samples with geometric mean concentrations within the established quantifiable range of the assay with ≤25% GCV. Overall percent recovery estimates for accuracy at the 1:5,000 dilution were 97% for the spike antigen, 95% for the nucleocapsid antigen, and 100% for the RBD antigen (Fig 4). Therefore, the percent recovery estimates were also within the prespecified range.

The LOD was set as the lowest Ab[C] that had a statistically significantly greater response than that in the ADHS sample. The LODs were 4.0, 4.0, and 3.0 AU/ml for the spike, nucleocapsid, and RBD antigens, respectively. As these values were lower than the LLOQ, the assays met the LOD acceptance criteria for each antigen.

#### Selectivity

The overall recovery estimates for the samples with expected concentration levels within the LOQs were 100% (90% CI: 97, 103) for the spike antigen, 100% (90% CI: 97, 104) for the nucleocapsid antigen, and 107% (90% CI: 102, 112) for the RBD antigen. All estimates were within acceptance criteria for selectivity–an overall percent recovery for concentrations within the LOQs between 66.7% and 150%.

#### Dilutional linearity

The assay showed dilutional linearity. The dilution fold-bias estimates per 10-fold sample dilution were 0.89 (95% CI: 0.83, 0.97), 0.91 (95% CI: 0.88, 0.94), and 0.88 (95% CI: 0.75, 1.02) for the spike, nucleocapsid, and RBD antigens, respectively. These values were below the pre-defined two-fold dilution bias limit for linearity.

#### Specificity

After competition with homologous antigens, 100% of the samples showed ≥75% inhibition after adsorption with the homologous antigens (SARS-CoV-2 N, SARS-CoV-2 S, and SARS-CoV-2 RBD), meeting the pre-defined specificity criterion. After competition with heterologous SARS-CoV-2 or heterologous seasonal coronavirus antigens, ≥93.8% of the samples showed ≤25% inhibition after adsorption with each respective SARS-CoV-2 heterologous assay antigen (cross-reactivity between the spike and RBD antigens is expected). Furthermore, ≥75% of the samples demonstrated ≤25% inhibition for adsorption with the heterologous seasonal coronavirus OC43 spike antigen (75.0%, 96.9%, and 100%, respectively). The pre-defined criterion for specificity was ≤25% inhibition when competed with each respective heterologous assay antigen. The overall specificity estimates are shown graphically in S1 Fig.

### Comparison of MNT and MSD ECL assay performance

**Fig 5:**
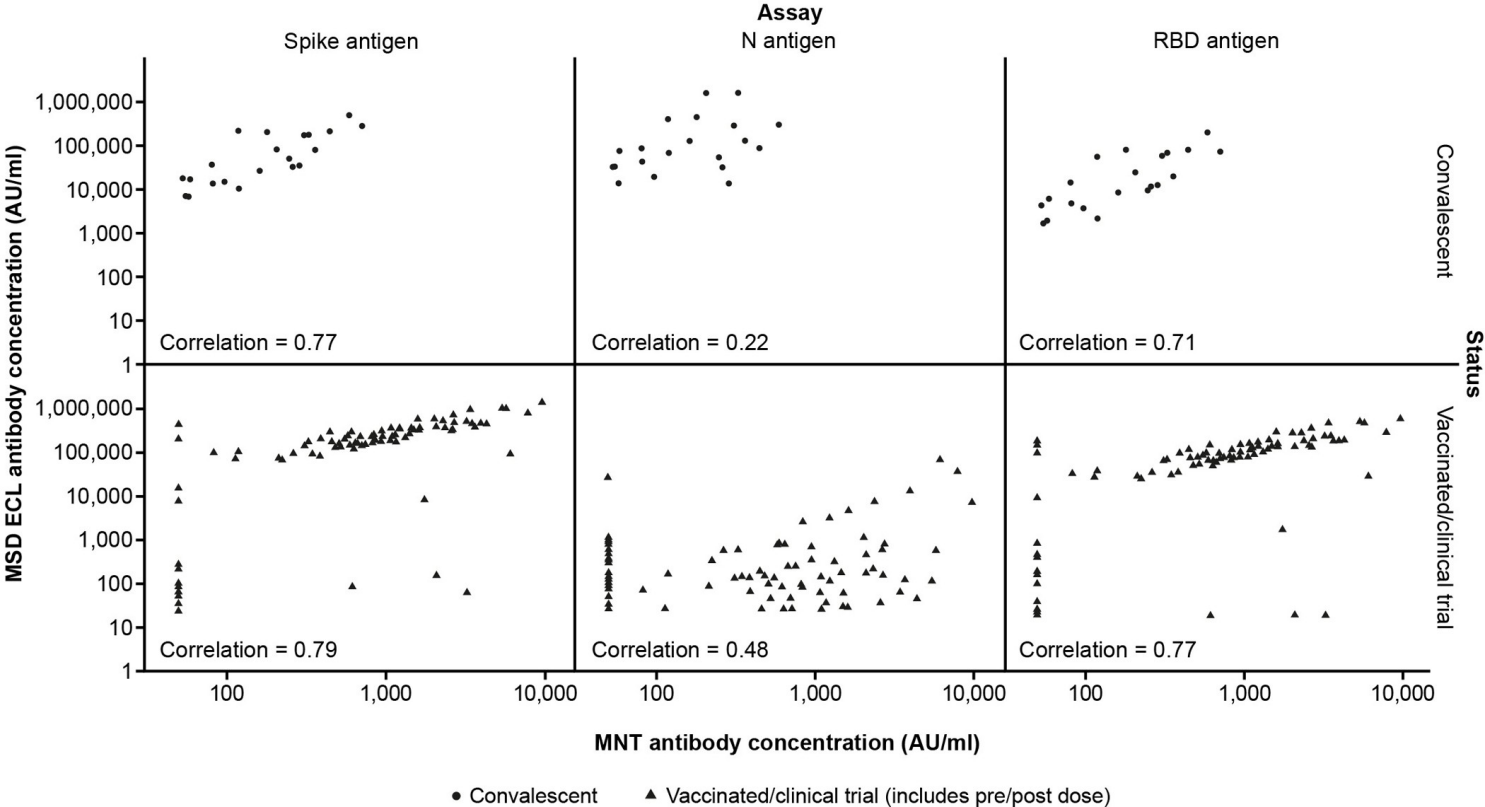
Correlation between MNT and MSD ECL assays in convalescent and vaccinated human samples. Comparison of assay results for convalescent sera samples (n = 32) and sera samples from vaccinated individuals (n = 52) from individuals who received one or two doses of mRNA-1273 or BNT162b2, and 30 pairs [Days 1 and 28 post-vaccination] from individuals vaccinated in a Moderna clinical trial) [8] using the microneutralization assay and in the multiplex ECL assay for detection IgG antibodies against SARS-CoV-2 spike, nucleocapsid, and receptor-binding domain antigens. ECL = electrochemiluminescence; IgG = immunoglobulin G; MNT = microneutralization assay; MSD ECL antibody concentration = antibody concentration as determined in the MSD multiplex electrochemiluminescence assay; N antigen = SARS-CoV-2 nucleocapsid protein antigen; RBD antigen = SARS-CoV-2 receptor-binding domain protein antigen; Spike antigen = SARS-CoV-2 spike protein antigen; SARS-CoV-2 = severe acute respiratory syndrome coronavirus 2.

Fig 5 shows the correlation between the results of the MNT and MSD ECL assays in convalescent and vaccinated human serum samples. Across 32 convalescent samples, the correlations between the MNT and the spike, nucleocapsid, and RBD MSD ECL assay Ab[C]s were 0.77, 0.22, and 0.71, respectively. Across samples from vaccinated individuals (52 samples from individuals who received one or two doses of mRNA vaccine mRNA-1273 or BNT162b2, and 30 pairs [Days 1 and 28 post-vaccination] from individuals who received mRNA-1273 in a Moderna clinical trial mRNA-1273-P201; NCT identifier NCT04405076 [8]), the correlations between the MNT and the spike, nucleocapsid, and RBD MSD ECL assay Ab[C]s were 0.79, 0.48, and 0.77, respectively. These results showed strong correlations between neutralizing activity in the samples as detected by the MNT assay and the concentrations of IgG antibodies against spike and RBD antigens in the MSD ECL assay. The correlation between nucleocapsid antibody detection and MNT assay activity was weak in convalescent samples, although there was a moderate correlation in the vaccinated samples, more than double that in the convalescent samples.

### MNT assay calibration

**Fig 6:**
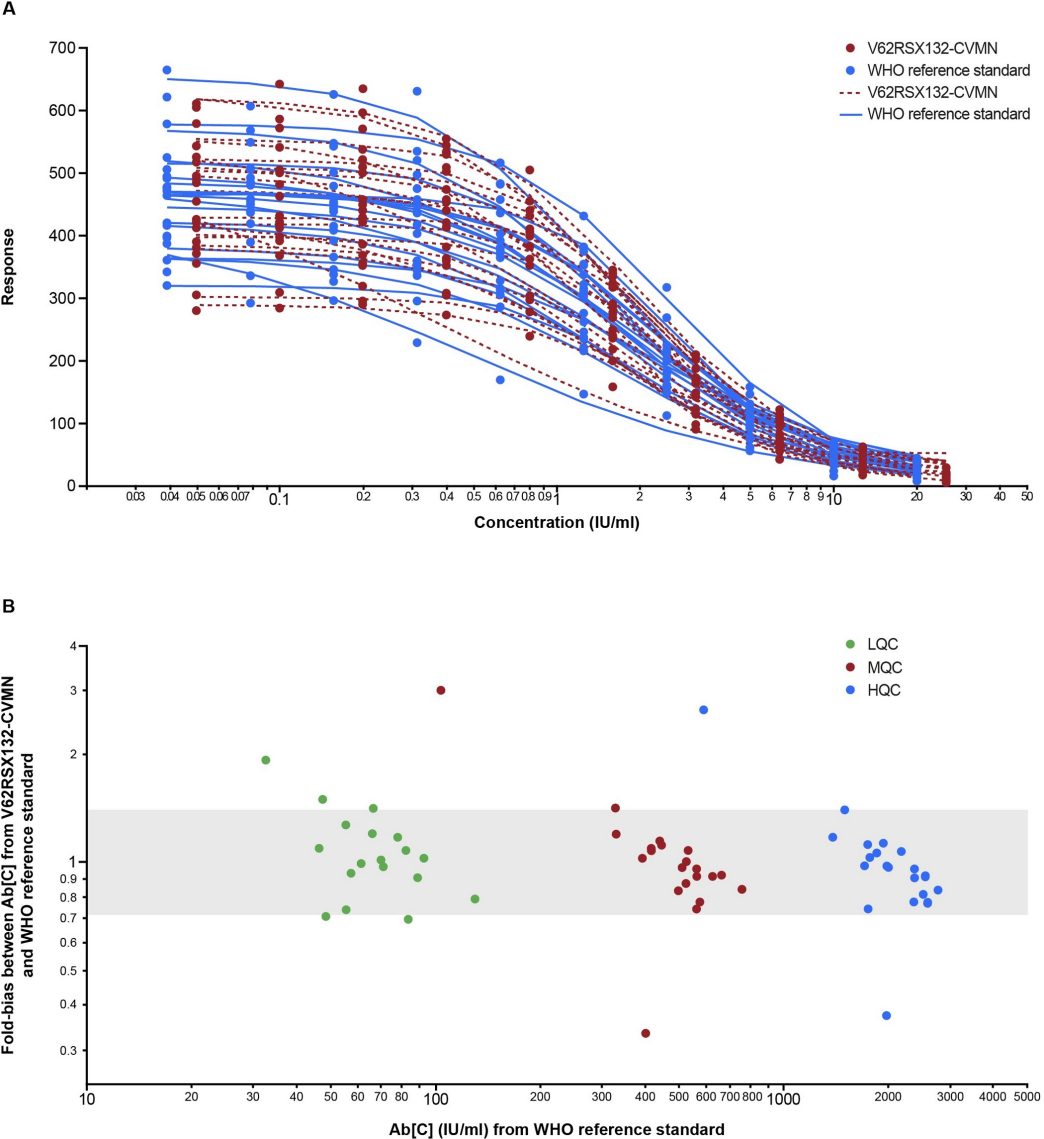
Calibration of MNT RS curve to WHO RS curve. Ten assay runs were performed across 25 days with each plate containing two independent preparations of the V62RSX132-CVMN and two independent preparations of the WHO RS. (A) An overlay of standard curves for V62RSX132-CVMN and WHO RS after application of new assignments for the V62RSX132-CVMN. (B) Fold-bias estimates comparing interpolated QCs Ab[C] (IU/ml) utilizing PPD V62RSX132-CVMN to interpolated QCs Ab[C] (IU/ml) utilizing WHO RS. Graph symbol numbers represent the run number, and the highlighted area represents ±1.40-fold. RS = reference standard.

The RS lot V62RSX132-CVMN was calibrated against WHO RS. The overlay of standard curves for V62RSX132-CVMN and WHO RS and a fold-bias for Ab[C] of QCs from both the RSs are depicted in Fig 6. The conversion factor relating IU/ml to AU/ml was determined as 1 IU/ml = 1.275 AU/ml. After application of the new concentration assignment to the V62RSX132-CVMN RS, the curves for the V62RSX132-CVMN RS and the WHO RS appear to be fairly well-aligned with few outliers (Fig 6A). Similarly, the QCs Ab[C]s obtained after applying the conversion factor to the QCs interpolated off the V62RSX132-CVMN RS compared to the QCs Ab[C]s interpolated off the WHO RS showed the majority of results to be within ±1.40-fold (Fig 6B, highlighted area).

## Discussion

We have described the design and criteria to validate immunogenicity assays and the outcomes of the validation of two immunogenicity assays to support the detection of SARS-CoV-2 antibodies in terms of precision, accuracy, dilutional linearity, selectivity, and specificity. We also compared the performance of these assays across a panel of the same SARS-CoV-2 convalescent patient serum samples used in the validation studies and in samples from vaccinated individuals. Both assays met all pre-specified acceptance criteria for validation and are acceptable for use in the assessment of antibody responses in clinical trial samples from Phase I studies and beyond.

Assay specificity is a key validation parameter that can present experimental design challenges. Specificity was demonstrated in both assays between SARS-CoV-2 antigens and against other seasonal coronaviruses using depletion experiments. However, the MNT assay required a modified approach. For the MNT assay, homologous and heterologous antigens were bound to magnetic beads prior to being used to deplete serum antibodies. To our knowledge, this is the first MNT assay developed for the detection of anti-SARS-CoV-2 neutralizing antibodies in human serum for which specificity has been demonstrated by means of antibody depletion by pre-incubation with homologous and heterologous proteins.

An additional unique characteristic of the MNT assay is the incorporation of an RS, developed in-house from a pool of convalescent sera, making it a fully quantitative assay. MNT assays using the traditional IC_50_ method for titer determination have an expected precision range of 30–60%, which is a typical range for functional assays such as MNT or opsonophagocytic killing assays. Adding a RS to the MNT assay enabled achievement of a much lower precision, with an overall assay variability (%GCV) of 25.3%. The evaluation of humoral and functional immune responses to various newly developed SARS-CoV-2 vaccines have utilized combinations of assays from several different sources [7, 11, 12], which does not enable immunogenicity to be directly compared between vaccines. In addition, the evaluation of neutralization responses using human serum panels lacks a well-controlled benchmark for comparisons between assays and vaccines [6]. The inclusion of a RS in the MNT assay, as described here, enables the reproducible quantitative evaluation of neutralizing antibody activity, providing a method that enables the direct comparison of immunogenicity endpoint data between vaccines [6, 7].

There was a clear correlation between the neutralizing activity, measured by the MNT assay, and the concentration of IgG antibodies measured by the MSD ECL assays against the virus spike and RBD proteins, but a weak correlation with the nucleocapsid protein. As the most abundant and potent neutralizing antibodies in convalescent patients target the spike and RBD proteins [13], the stronger correlation between spike and RBD IgG levels measured in the MSD ECL assay and the neutralizing activity in the MNT assay was in line with the immune response observed in convalescent patients [13]. Therefore, these assays enable the evaluation of both functional immune responses (i.e., neutralization) and the levels of specific IgG antibodies produced in response to vaccination, as candidate vaccines mainly target the virus spike and RBD proteins [4]. Thus, these assays enable the definition of a correlate of vaccine-induced protection as proposed under the WHO and FDA guidance [4, 5].

WHO International Reference Panels are now available from the National Institute for Biological Standards and Control (NIBSC) for use in the development, evaluation, and calibration of anti-SARS-CoV-2 serologic assays [14, 15]. It is noteworthy that MSD has now calibrated the RS used in the MSD ECL assay to the NIBSC international RS (NIBSC code: 20/136) and results for the MSD ECL assay can now be reported in international units (binding antibody units [BAU]/ml). The present study calibrated the MNT assay RS to the NIBSC international RS (NIBSC code 20/136); with the conversion factor of 1 IU/ml = 1.275 AU/ml, the concentration assignment for the PPD V62RSX132-CVMN RS is 1275 IU/ml. The calibration of both the MSD and MNT assays to the WHO International Reference Panels allows for comparison of vaccine immunogenicity data between different assays and laboratories and, ultimately, between vaccine manufacturers to evaluate the comparability of humoral and functional immune responses to various SARS-CoV-2 vaccines and/or vaccination regimens. In addition, with the emergence of SARS-CoV-2 variants, additional antigens and pseudoviruses are being evaluated in the MNT and MSD ECL assays, including the B.1.351 variant (which first emerged in South Africa), the P.1 variant (which first emerged in Brazil), and the B.1.1.7 variant (which first emerged in the United Kingdom).

## Conclusions

In conclusion, availability of the validated MNT and MSD ECL assays for the assessment of functional immune response and the Ab[C]s generated in response to SARS-CoV-2 vaccines may contribute to fulfilling the recommendations of the WHO and FDA considerations and guidance for the development and evaluation of SARS-CoV-2 vaccines [4, 5]. These assays could also provide a standardized approach for accurately comparing immune responses to different vaccines and/or vaccination regimens.

## Supporting information

S1 Fig Specificity analysis results for the microneutralization assay and the multiplex ECL assay, showing the percentage inhibition of homologous and heterologous competitor antigens. Covid MNT = coronavirus disease microneutralization assay; ECL = electrochemiluminescence; HC OC43 = human seasonal coronavirus OC43; MSD ECL CoV-2 N = Meso Scale Discovery’s multiplex electrochemiluminescence assay for SARS-CoV-2 nucleocapsid protein; MSD ECL CoV-2 S = Meso Scale Discovery’s multiplex electrochemiluminescence assay for SARS-CoV-2 spike protein; MSD ECL CoV-2 RBD = Meso Scale Discovery’s multiplex electrochemiluminescence assay for SARS-CoV-2 receptor-binding domain protein; RBD = receptor-binding protein.
(TIF)

S1 Table MNT assay precision profiles. %GCV = percent geometric coefficient of variation; GMC = geometric mean concentration; HQC = high quality control; LQC = low quality control; MNT = microneutralization; MQC = mid quality control.
(PDF)

S2 Table MNT assay accuracy. Ab[C] = antibody concentration; MNT = microneutralization; NE = not estimable.
(PDF)

S3 Table MSD ECL assay precision profiles: (A) spike, (B) nucleocapsid, and (C) receptor-binding domain antigens. A = accuracy; GMC = geometric mean concentration; %GCV = percent geometric coefficient of variation; MSD ECL = multiplex electrochemiluminescence; N = nucleocapsid; P = precision; RBD = receptor-binding domain; S = spike; SARS-CoV-2 = severe acute respiratory syndrome coronavirus 2.
(PDF)

S4 Table MSD ECL assay accuracy: (A) spike, (B) nucleocapsid, and (C) receptor-binding domain antigens. MSD ECL = multiplex electrochemiluminescence; N = nucleocapsid; NE = not estimable; RBD = receptor-binding domain; S = spike; SARS-CoV-2 = severe acute respiratory syndrome coronavirus 2.
(PDF)

S5 Table Correlation between the results of the MNT and MSD ECL assays: (A) spike, (B) nucleocapsid, and (C) receptor-binding domain antigens. Ab[C] = antibody concentration; MNT = microneutralization; MSD ECL = multiplex electrochemiluminescence; N = nucleocapsid; NE = not estimable; RBD = receptor-binding domain; S = spike; SARS-CoV-2 = severe acute respiratory syndrome coronavirus 2.
(PDF)

S6 Table MNT reference standard calibration to WHO international reference panel. MNT = microneutralization; PPD = Pharmaceutical Product Development; WHO = World Health Organization.
(PDF)

S7 Table Specificity analysis: (A) Competitors used (B) MSD ECL assay, and (C) MNT assay. Ab[C] = antibody concentrations; AU = arbitrary units; MNT = microneutralization; MSD ECL = multiplex electrochemiluminescence; N = nucleocapsid; PBS = phosphate-buffered saline; RBD = receptor-binding domain; S = spike; SARS-CoV-2 = severe acute respiratory syndrome coronavirus 2.
(PDF)
