# Supplementary figures and images for: Robust validation and performance comparison of immunogenicity assays assessing IgG and neutralizing antibodies to SARS-CoV-2
Source: PLoS One. 2022 Feb 7;17(2):e0262922. doi: 10.1371/journal.pone.0262922 (PMC8820625; doi:10.1371/journal.pone.0262922)

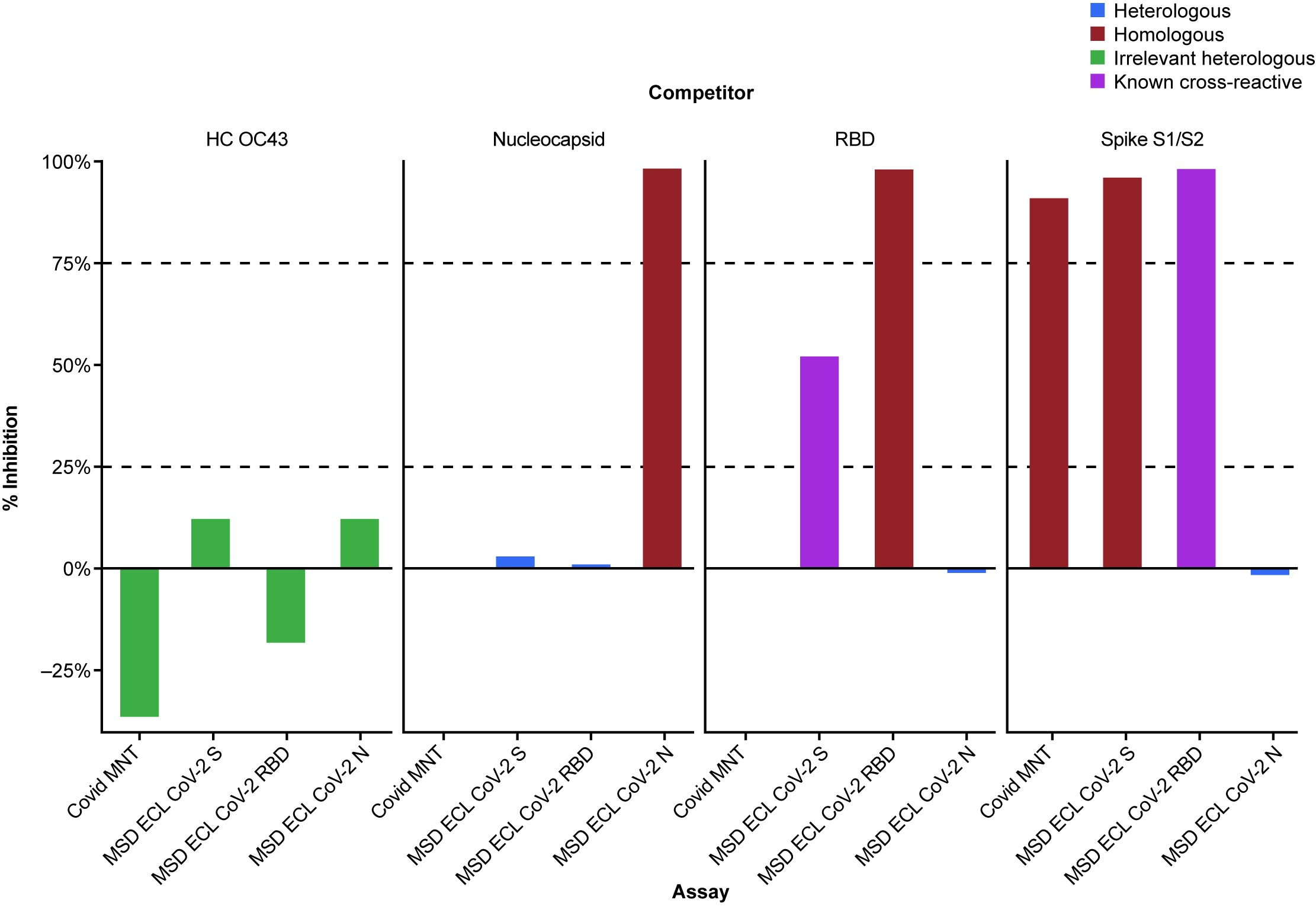

Supplement: S1 Fig — Covid MNT = coronavirus disease microneutralization assay; ECL = electrochemiluminescence; HC OC43 = human seasonal coronavirus OC43; MSD ECL CoV-2 N = Meso Scale Discovery’s multiplex electrochemiluminescence assay for SARS-CoV-2 nucleocapsid protein; MSD ECL CoV-2 S = Meso Scale Discovery’s multiplex electrochemiluminescence assay for SARS-CoV-2 spike protein; MSD ECL CoV-2 RBD = Meso Scale Discovery’s multiplex electrochemiluminescence assay for SARS-CoV-2 receptor-binding domain protein; RBD = receptor-binding protein. (TIF) [file pone.0262922.s001.tif]
