# Supplementary material for: Robust validation and performance comparison of immunogenicity assays assessing IgG and neutralizing antibodies to SARS-CoV-2
Source: PLoS One. 2022 Feb 7;17(2):e0262922. doi: 10.1371/journal.pone.0262922 (PMC8820625; doi:10.1371/journal.pone.0262922)
Supplement: S1 Table — %GCV = percent geometric coefficient of variation; GMC = geometric mean concentration; HQC = high quality control; LQC = low quality control; MNT = microneutralization; MQC = mid quality control. (PDF) [file pone.0262922.s002.pdf]

S1 Table. MNT assay precision profiles.

Sample	GMC (AU/ml)	%GCV
1	18	100.00
2	40	48.14
3	79	20.95
4	37	55.88
5	65	19.18
6	98	18.61
7	96	20.65
8	368	34.14
9	193	16.49
10	116	54.00
11	166	22.25
12	173	19.42
13	236	19.09
14	304	26.90
15	239	14.61
16	340	22.52
17	470	22.99
18	496	56.94
19	576	15.20
20	65	25.53
21	473	24.66
22	1197	22.49
23	1657	22.19
24	14	100.00
HQC	1167	25.18
LQC	59	23.66
MQC	217	21.16

AU = arbitrary units; %GCV = percent geometric coefficient of variation; GMC = geometric mean concentration; HQC = high quality control; LQC = low quality control; MNT = microneutralization; MQC = mid quality control.
