# Supplementary material for: Robust validation and performance comparison of immunogenicity assays assessing IgG and neutralizing antibodies to SARS-CoV-2
Source: PLoS One. 2022 Feb 7;17(2):e0262922. doi: 10.1371/journal.pone.0262922 (PMC8820625; doi:10.1371/journal.pone.0262922)
Supplement: S2 Table — Ab[C] = antibody concentration; MNT = microneutralization; NE = not estimable. (PDF) [file pone.0262922.s003.pdf]

S2 Table. MNT assay accuracy.

Run	Expected Ab[C] (AU/ml)	% Recovery	Run	Expected Ab[C] (AU/ml)	% Recovery
1	7.8	6.912	5	7.8	NE
1	15.6	NE	5	15.6	NE
1	31.3	NE	5	31.3	NE
1	62.5	0.912	5	62.5	NE
1	125	0.664	5	125	NE
1	250	1.004	5	250	NE
1	500	0.664	5	500	NE
1	1000	1.001	5	1000	NE
2	7.8	NE	6	7.8	NE
2	15.6	NE	6	15.6	NE
2	31.3	NE	6	31.3	NE
2	62.5	NE	6	62.5	NE
2	125	NE	6	125	NE
2	250	NE	6	250	NE
2	500	NE	6	500	NE
2	1000	NE	6	1000	NE
3	7.8	4.864	7	7.8	0.4
3	15.6	1.856	7	15.6	0.4
3	31.3	1.28	7	31.3	1.376
3	62.5	1.12	7	62.5	1.088
3	125	1.44	7	125	1.296
3	250	NE	7	250	0.716
3	500	0.87	7	500	1.018
3	1000	0.917	7	1000	1.646
4	7.8	1.664	8	7.8	0.512
4	15.6	1.344	8	15.6	0.064
4	31.3	0.928	8	31.3	0.64
4	62.5	0.912	8	62.5	1.216
4	125	1.176	8	125	1.04
4	250	1.036	8	250	1.396
4	500	1.172	8	500	1.098
4	1000	0.97	8	1000	0.906

Ab[C] = antibody concentration; AU = arbitrary units; MNT = microneutralization; NE = not estimable.
