# Supplementary material for: Robust validation and performance comparison of immunogenicity assays assessing IgG and neutralizing antibodies to SARS-CoV-2
Source: PLoS One. 2022 Feb 7;17(2):e0262922. doi: 10.1371/journal.pone.0262922 (PMC8820625; doi:10.1371/journal.pone.0262922)
Supplement: S3 Table — MSD ECL assay precision profiles: (A) spike, (B) nucleocapsid, and (C) receptor-binding domain antigens. A = accuracy; GMC = geometric mean concentration; %GCV = percent geometric coefficient of variation; MSD ECL = multiplex electrochemiluminescence; N = nucleocapsid; P = precision; RBD = receptor-binding domain; S = spike; SARS-CoV-2 = severe acute respiratory syndrome coronavirus 2. (PDF) [file pone.0262922.s004.pdf]

S3 Table. MSD ECL assay precision profiles.

(A) Spike antigen

Antigen	Dilution	Sample #	Panel	%GCV	GMC (AU/ml)
SARS-CoV-2 S	500	1	A	3.73%	22848
SARS-CoV-2 S	500	2	A	4.29%	8828
SARS-CoV-2 S	500	3	A	5.32%	3381
SARS-CoV-2 S	500	4	A	5.13%	1351
SARS-CoV-2 S	500	5	A	6.04%	537
SARS-CoV-2 S	500	6	A	6.01%	221
SARS-CoV-2 S	500	7	A	6.68%	88
SARS-CoV-2 S	500	8	A	5.91%	35
SARS-CoV-2 S	500	9	A	8.63%	13
SARS-CoV-2 S	500	10	A	29.90%	4
SARS-CoV-2 S	500	1	P	6.69%	122297
SARS-CoV-2 S	500	2	P	4.05%	56141
SARS-CoV-2 S	500	3	P	4.95%	9150
SARS-CoV-2 S	500	4	P	4.59%	95969
SARS-CoV-2 S	500	5	P	2.95%	4131
SARS-CoV-2 S	500	6	P	4.72%	15432
SARS-CoV-2 S	500	7	P	4.38%	8575
SARS-CoV-2 S	500	8	P	7.39%	13594
SARS-CoV-2 S	500	9	P	8.96%	37201
SARS-CoV-2 S	500	10	P	3.38%	29917
SARS-CoV-2 S	500	11	P	4.88%	8745
SARS-CoV-2 S	500	12	P	7.17%	856
SARS-CoV-2 S	500	13	P	9.50%	76
SARS-CoV-2 S	500	14	P	13.85%	2655
SARS-CoV-2 S	500	15	P	18.31%	36
SARS-CoV-2 S	500	16	P	18.68%	35
SARS-CoV-2 S	500	17	P	20.68%	17
SARS-CoV-2 S	500	18	P	13.85%	20
SARS-CoV-2 S	500	19	P	16.07%	27
SARS-CoV-2 S	500	20	P	13.05%	24
SARS-CoV-2 S	500	21	P	19.87%	20

A = accuracy; AU = arbitrary units; %GCV = percent geometric coefficient of variation; GMC = geometric mean concentration; MSD ECL = multiplex electrochemiluminescence; P = precision; S = spike; SARS-CoV-2 = severe acute respiratory syndrome coronavirus 2.

(A) Spike antigen (continued)

Antigen	Dilution	Sample #	Panel	%GCV	GMC (AU/ml)
SARS-CoV-2 S	5000	1	Q	4.42%	1960
SARS-CoV-2 S	5000	1	A	4.45%	19489
SARS-CoV-2 S	5000	2	A	3.79%	7647
SARS-CoV-2 S	5000	3	A	5.14%	3100
SARS-CoV-2 S	5000	4	A	5.04%	1273
SARS-CoV-2 S	5000	5	A	4.99%	518
SARS-CoV-2 S	5000	6	A	7.99%	205
SARS-CoV-2 S	5000	7	A	14.78%	78
SARS-CoV-2 S	5000	8	A	100.00%	20
SARS-CoV-2 S	5000	9	A	100.00%	4
SARS-CoV-2 S	5000	10	A	100.00%	2
SARS-CoV-2 S	5000	1	P	4.14%	51171
SARS-CoV-2 S	5000	2	P	7.63%	210860
SARS-CoV-2 S	5000	3	P	3.71%	7041
SARS-CoV-2 S	5000	4	P	4.43%	97851
SARS-CoV-2 S	5000	5	P	4.08%	3222
SARS-CoV-2 S	5000	6	P	5.85%	13418
SARS-CoV-2 S	5000	7	P	7.50%	7285
SARS-CoV-2 S	5000	8	P	4.65%	12695
SARS-CoV-2 S	5000	9	P	6.91%	32626
SARS-CoV-2 S	5000	10	P	6.14%	27550
SARS-CoV-2 S	5000	11	P	5.77%	7833
SARS-CoV-2 S	5000	12	P	8.97%	849
SARS-CoV-2 S	5000	13	P	75.18%	79
SARS-CoV-2 S	5000	14	P	17.79%	2726
SARS-CoV-2 S	5000	15	P	100.00%	21
SARS-CoV-2 S	5000	16	P	100.00%	18
SARS-CoV-2 S	5000	17	P	100.00%	4
SARS-CoV-2 S	5000	18	P	100.00%	6
SARS-CoV-2 S	5000	19	P	100.00%	13
SARS-CoV-2 S	5000	20	P	100.00%	11
SARS-CoV-2 S	5000	21	P	100.00%	5

A = accuracy; AU = arbitrary units; %GCV = percent geometric coefficient of variation; GMC = geometric mean concentration; P = precision; Q = quality control; S = spike; SARS-CoV-2 = severe acute respiratory syndrome coronavirus 2.

(B) Nucleocapsid antigen

Antigen	Dilution	Sample #	Panel	%GCV	GMC (AU/ml)
SARS-CoV-2 N	500	1	A	3.16%	23874
SARS-CoV-2 N	500	2	A	3.79%	9082
SARS-CoV-2 N	500	3	A	3.97%	3743
SARS-CoV-2 N	500	4	A	4.78%	1458
SARS-CoV-2 N	500	5	A	5.02%	580
SARS-CoV-2 N	500	6	A	5.31%	232
SARS-CoV-2 N	500	7	A	6.05%	95
SARS-CoV-2 N	500	8	A	6.21%	35
SARS-CoV-2 N	500	9	A	7.38%	11
SARS-CoV-2 N	500	10	A	63.80%	2
SARS-CoV-2 N	500	1	P	4.72%	174060
SARS-CoV-2 N	500	2	P	4.59%	60442
SARS-CoV-2 N	500	3	P	6.84%	83517
SARS-CoV-2 N	500	4	P	10.84%	198385
SARS-CoV-2 N	500	5	P	3.92%	14570
SARS-CoV-2 N	500	6	P	5.03%	44523
SARS-CoV-2 N	500	7	P	4.87%	12693
SARS-CoV-2 N	500	8	P	9.67%	20202
SARS-CoV-2 N	500	9	P	5.04%	106852
SARS-CoV-2 N	500	10	P	2.97%	143928
SARS-CoV-2 N	500	11	P	5.75%	27877
SARS-CoV-2 N	500	12	P	4.49%	38048
SARS-CoV-2 N	500	13	P	7.51%	1229
SARS-CoV-2 N	500	14	P	4.66%	3099
SARS-CoV-2 N	500	15	P	10.36%	74
SARS-CoV-2 N	500	16	P	30.26%	42
SARS-CoV-2 N	500	17	P	12.70%	39
SARS-CoV-2 N	500	18	P	7.81%	296
SARS-CoV-2 N	500	19	P	24.19%	105
SARS-CoV-2 N	500	20	P	13.70%	73
SARS-CoV-2 N	500	21	P	55.08%	182

A = accuracy; AU = arbitrary units; %GCV = percent geometric coefficient of variation; GMC = geometric mean concentration; N = nucleocapsid; P = precision; SARS-CoV-2 = severe acute respiratory syndrome coronavirus 2.

(B) Nucleocapsid antigen (continued)

Antigen	Dilution	Sample #	Panel	%GCV	GMC (AU/ml)
SARS-CoV-2 N	5000	1	Q	3.69%	5008
SARS-CoV-2 N	5000	1	A	6.48%	20524
SARS-CoV-2 N	5000	2	A	4.86%	7864
SARS-CoV-2 N	5000	3	A	6.57%	3370
SARS-CoV-2 N	5000	4	A	4.84%	1350
SARS-CoV-2 N	5000	5	A	5.69%	533
SARS-CoV-2 N	5000	6	A	6.49%	207
SARS-CoV-2 N	5000	7	A	6.80%	71
SARS-CoV-2 N	5000	8	A	100.00%	11
SARS-CoV-2 N	5000	9	A	0.00%	1
SARS-CoV-2 N	5000	10	A	0.00%	1
SARS-CoV-2 N	5000	1	P	7.58%	213826
SARS-CoV-2 N	5000	2	P	5.64%	54748
SARS-CoV-2 N	5000	3	P	4.29%	69313
SARS-CoV-2 N	5000	4	P	5.03%	510056
SARS-CoV-2 N	5000	5	P	7.88%	11932
SARS-CoV-2 N	5000	6	P	5.36%	41297
SARS-CoV-2 N	5000	7	P	9.61%	11144
SARS-CoV-2 N	5000	8	P	4.47%	18849
SARS-CoV-2 N	5000	9	P	8.42%	108632
SARS-CoV-2 N	5000	10	P	13.10%	177818
SARS-CoV-2 N	5000	11	P	5.86%	26170
SARS-CoV-2 N	5000	12	P	6.80%	33684
SARS-CoV-2 N	5000	13	P	10.20%	1083
SARS-CoV-2 N	5000	14	P	7.40%	2894
SARS-CoV-2 N	5000	15	P	45.02%	56
SARS-CoV-2 N	5000	16	P	100.00%	8
SARS-CoV-2 N	5000	17	P	100.00%	15
SARS-CoV-2 N	5000	18	P	12.05%	272
SARS-CoV-2 N	5000	19	P	18.64%	86
SARS-CoV-2 N	5000	20	P	35.86%	49
SARS-CoV-2 N	5000	21	P	57.18%	71

A = accuracy; AU = arbitrary units; %GCV = percent geometric coefficient of variation; GMC = geometric mean concentration; N = nucleocapsid; P = precision; Q = quality control; SARS-CoV-2 = severe acute respiratory syndrome coronavirus 2.

(C) Receptor-binding domain antigens

Antigen	Dilution	Sample #	Panel	%GCV	GMC (AU/ml)
SARS-CoV-2 RBD	500	1	A	7.91%	5123
SARS-CoV-2 RBD	500	2	A	7.06%	1880
SARS-CoV-2 RBD	500	3	A	14.07%	767
SARS-CoV-2 RBD	500	4	A	11.36%	306
SARS-CoV-2 RBD	500	5	A	16.40%	129
SARS-CoV-2 RBD	500	6	A	6.21%	50
SARS-CoV-2 RBD	500	7	A	7.51%	19
SARS-CoV-2 RBD	500	8	A	18.50%	6
SARS-CoV-2 RBD	500	9	A	33.24%	2
SARS-CoV-2 RBD	500	10	A	0.00%	1
SARS-CoV-2 RBD	500	1	P	13.18%	55482
SARS-CoV-2 RBD	500	2	P	10.76%	12768
SARS-CoV-2 RBD	500	3	P	8.72%	2951
SARS-CoV-2 RBD	500	4	P	12.49%	37892
SARS-CoV-2 RBD	500	5	P	7.39%	1055
SARS-CoV-2 RBD	500	6	P	7.21%	5086
SARS-CoV-2 RBD	500	7	P	12.03%	3061
SARS-CoV-2 RBD	500	8	P	10.09%	4071
SARS-CoV-2 RBD	500	9	P	8.54%	13888
SARS-CoV-2 RBD	500	10	P	6.84%	8239
SARS-CoV-2 RBD	500	11	P	6.25%	3224
SARS-CoV-2 RBD	500	12	P	7.85%	192
SARS-CoV-2 RBD	500	13	P	16.27%	93
SARS-CoV-2 RBD	500	14	P	5.38%	709
SARS-CoV-2 RBD	500	15	P	14.72%	33
SARS-CoV-2 RBD	500	16	P	19.00%	25
SARS-CoV-2 RBD	500	17	P	22.09%	22
SARS-CoV-2 RBD	500	18	P	33.41%	15
SARS-CoV-2 RBD	500	19	P	33.61%	61
SARS-CoV-2 RBD	500	20	P	22.06%	23
SARS-CoV-2 RBD	500	21	P	34.58%	15

A = accuracy; AU = arbitrary units; %GCV = percent geometric coefficient of variation; GMC = geometric mean concentration; P = precision; RBD = receptor-binding domain; SARS-CoV-2 = severe acute respiratory syndrome coronavirus 2.

(C) Receptor-binding domain antigens (continued)

Antigen	Dilution	Sample #	Panel	%GCV	GMC (AU/ml)
SARS-CoV-2 RBD	5000	1	Q	7.71%	622
SARS-CoV-2 RBD	5000	1	A	6.95%	4359
SARS-CoV-2 RBD	5000	2	A	8.95%	1681
SARS-CoV-2 RBD	5000	3	A	9.34%	709
SARS-CoV-2 RBD	5000	4	A	10.97%	283
SARS-CoV-2 RBD	5000	5	A	8.00%	121
SARS-CoV-2 RBD	5000	6	A	16.37%	47
SARS-CoV-2 RBD	5000	7	A	100.00%	12
SARS-CoV-2 RBD	5000	8	A	100.00%	4
SARS-CoV-2 RBD	5000	9	A	100.00%	3
SARS-CoV-2 RBD	5000	10	A	100.00%	2
SARS-CoV-2 RBD	5000	1	P	14.42%	112921
SARS-CoV-2 RBD	5000	2	P	6.87%	10802
SARS-CoV-2 RBD	5000	3	P	9.95%	2397
SARS-CoV-2 RBD	5000	4	P	17.33%	37260
SARS-CoV-2 RBD	5000	5	P	10.73%	873
SARS-CoV-2 RBD	5000	6	P	4.91%	4774
SARS-CoV-2 RBD	5000	7	P	11.15%	2570
SARS-CoV-2 RBD	5000	8	P	7.49%	3812
SARS-CoV-2 RBD	5000	9	P	9.43%	12453
SARS-CoV-2 RBD	5000	10	P	8.71%	7271
SARS-CoV-2 RBD	5000	11	P	11.21%	3068
SARS-CoV-2 RBD	5000	12	P	19.50%	206
SARS-CoV-2 RBD	5000	13	P	38.65%	109
SARS-CoV-2 RBD	5000	14	P	11.29%	694
SARS-CoV-2 RBD	5000	15	P	100.00%	30
SARS-CoV-2 RBD	5000	16	P	68.78%	22
SARS-CoV-2 RBD	5000	17	P	100.00%	21
SARS-CoV-2 RBD	5000	18	P	96.98%	18
SARS-CoV-2 RBD	5000	19	P	31.38%	72
SARS-CoV-2 RBD	5000	20	P	92.25%	27
SARS-CoV-2 RBD	5000	21	P	100.00%	16

A = accuracy; AU = arbitrary units; %GCV = percent geometric coefficient of variation; GMC = geometric mean concentration; P = precision; Q = quality control; RBD = receptor-binding domain; SARS-CoV-2 = severe acute respiratory syndrome coronavirus 2.
