# Supplementary material for: Robust validation and performance comparison of immunogenicity assays assessing IgG and neutralizing antibodies to SARS-CoV-2
Source: PLoS One. 2022 Feb 7;17(2):e0262922. doi: 10.1371/journal.pone.0262922 (PMC8820625; doi:10.1371/journal.pone.0262922)
Supplement: S4 Table — MSD ECL assay accuracy: (A) spike, (B) nucleocapsid, and (C) receptor-binding domain antigens. MSD ECL = multiplex electrochemiluminescence; N = nucleocapsid; NE = not estimable; RBD = receptor-binding domain; S = spike; SARS-CoV-2 = severe acute respiratory syndrome coronavirus 2. (PDF) [file pone.0262922.s005.pdf]

S4 Table. MSD ECL assay accuracy.

(A) Spike antigen

Dilution	Antigen	Sample #	Expected concentration (AU/ml)	% Recovery
500	SARS-CoV-2 S	1	51171.00	1.13
500	SARS-CoV-2 S	1	20468.40	1.16
500	SARS-CoV-2 S	1	8187.36	1.11
500	SARS-CoV-2 S	1	3274.94	1.00
500	SARS-CoV-2 S	1	1309.98	1.00
500	SARS-CoV-2 S	1	523.99	1.00
500	SARS-CoV-2 S	1	209.60	1.02
500	SARS-CoV-2 S	1	83.84	0.98
500	SARS-CoV-2 S	1	33.54	0.98
500	SARS-CoV-2 S	1	13.41	0.89
500	SARS-CoV-2 S	1	5.37	0.75
5000	SARS-CoV-2 S	1	51171.00	1.07
5000	SARS-CoV-2 S	1	20468.40	0.98
5000	SARS-CoV-2 S	1	8187.36	0.92
5000	SARS-CoV-2 S	1	3274.94	0.91
5000	SARS-CoV-2 S	1	1309.98	0.93
5000	SARS-CoV-2 S	1	523.99	0.96
5000	SARS-CoV-2 S	1	209.60	0.93
5000	SARS-CoV-2 S	1	83.84	0.83
5000	SARS-CoV-2 S	1	33.54	0.45
5000	SARS-CoV-2 S	1	13.41	NE
5000	SARS-CoV-2 S	1	5.37	NE
50000	SARS-CoV-2 S	1	51171.00	0.88
50000	SARS-CoV-2 S	1	20468.40	0.86
50000	SARS-CoV-2 S	1	8187.36	0.87
50000	SARS-CoV-2 S	1	3274.94	0.91
50000	SARS-CoV-2 S	1	1309.98	0.92
50000	SARS-CoV-2 S	1	523.99	0.83
50000	SARS-CoV-2 S	1	209.60	1.01
50000	SARS-CoV-2 S	1	83.84	0.40
50000	SARS-CoV-2 S	1	33.54	NE
50000	SARS-CoV-2 S	1	13.41	NE
50000	SARS-CoV-2 S	1	5.37	NE
500	SARS-CoV-2 S	2	51171.00	1.08
500	SARS-CoV-2 S	2	20468.40	1.09
500	SARS-CoV-2 S	2	8187.36	1.02
500	SARS-CoV-2 S	2	3274.94	1.03
500	SARS-CoV-2 S	2	1309.98	0.99
500	SARS-CoV-2 S	2	523.99	1.02
500	SARS-CoV-2 S	2	209.60	1.05
500	SARS-CoV-2 S	2	83.84	1.07
500	SARS-CoV-2 S	2	33.54	1.04
500	SARS-CoV-2 S	2	13.41	0.97
500	SARS-CoV-2 S	2	5.37	0.93
5000	SARS-CoV-2 S	2	51171.00	0.99
5000	SARS-CoV-2 S	2	20468.40	0.93

5000	SARS-CoV-2 S	2	8187.36	0.92
5000	SARS-CoV-2 S	2	3274.94	0.94
5000	SARS-CoV-2 S	2	1309.98	0.95
5000	SARS-CoV-2 S	2	523.99	1.01
5000	SARS-CoV-2 S	2	209.60	1.01
5000	SARS-CoV-2 S	2	83.84	0.94
5000	SARS-CoV-2 S	2	33.54	0.51
5000	SARS-CoV-2 S	2	13.41	NE
5000	SARS-CoV-2 S	2	5.37	NE
50000	SARS-CoV-2 S	2	51171.00	0.94
50000	SARS-CoV-2 S	2	20468.40	0.91
50000	SARS-CoV-2 S	2	8187.36	0.94
50000	SARS-CoV-2 S	2	3274.94	0.98
50000	SARS-CoV-2 S	2	1309.98	1.00
50000	SARS-CoV-2 S	2	523.99	1.06
50000	SARS-CoV-2 S	2	209.60	1.25
50000	SARS-CoV-2 S	2	83.84	2.00
50000	SARS-CoV-2 S	2	33.54	NE
50000	SARS-CoV-2 S	2	13.41	NE
50000	SARS-CoV-2 S	2	5.37	NE

AU = arbitrary units; MSD ECL = multiplex electrochemiluminescence; NE = not estimable; S = spike; SARS-CoV-2 = severe acute respiratory syndrome coronavirus 2.

(A) Spike antigen (continued)

Dilution	Antigen	Sample #	Expected concentration (AU/ml)	% Recovery
500	SARS-CoV-2 S	3	51171.00	1.07
500	SARS-CoV-2 S	3	20468.40	1.12
500	SARS-CoV-2 S	3	8187.36	1.10
500	SARS-CoV-2 S	3	3274.94	1.08
500	SARS-CoV-2 S	3	1309.98	1.09
500	SARS-CoV-2 S	3	523.99	1.07
500	SARS-CoV-2 S	3	209.60	1.11
500	SARS-CoV-2 S	3	83.84	1.16
500	SARS-CoV-2 S	3	33.54	1.10
500	SARS-CoV-2 S	3	13.41	1.12
500	SARS-CoV-2 S	3	5.37	0.75
5000	SARS-CoV-2 S	3	51171.00	1.02
5000	SARS-CoV-2 S	3	20468.40	0.99
5000	SARS-CoV-2 S	3	8187.36	0.97
5000	SARS-CoV-2 S	3	3274.94	0.98
5000	SARS-CoV-2 S	3	1309.98	1.00
5000	SARS-CoV-2 S	3	523.99	1.01
5000	SARS-CoV-2 S	3	209.60	1.04
5000	SARS-CoV-2 S	3	83.84	1.00
5000	SARS-CoV-2 S	3	33.54	0.83
5000	SARS-CoV-2 S	3	13.41	0.40
5000	SARS-CoV-2 S	3	5.37	NE
50000	SARS-CoV-2 S	3	51171.00	1.01
50000	SARS-CoV-2 S	3	20468.40	0.96
50000	SARS-CoV-2 S	3	8187.36	1.04
50000	SARS-CoV-2 S	3	3274.94	1.00
50000	SARS-CoV-2 S	3	1309.98	1.01
50000	SARS-CoV-2 S	3	523.99	0.86
50000	SARS-CoV-2 S	3	209.60	0.93
50000	SARS-CoV-2 S	3	83.84	NE
50000	SARS-CoV-2 S	3	33.54	NE
50000	SARS-CoV-2 S	3	13.41	NE
50000	SARS-CoV-2 S	3	5.37	NE
500	SARS-CoV-2 S	4	51171.00	1.15
500	SARS-CoV-2 S	4	20468.40	1.17
500	SARS-CoV-2 S	4	8187.36	1.11
500	SARS-CoV-2 S	4	3274.94	1.08
500	SARS-CoV-2 S	4	1309.98	1.09
500	SARS-CoV-2 S	4	523.99	1.09
500	SARS-CoV-2 S	4	209.60	1.13
500	SARS-CoV-2 S	4	83.84	1.11
500	SARS-CoV-2 S	4	33.54	1.10
500	SARS-CoV-2 S	4	13.41	0.97
500	SARS-CoV-2 S	4	5.37	0.93
5000	SARS-CoV-2 S	4	51171.00	0.97
5000	SARS-CoV-2 S	4	20468.40	0.93

5000	SARS-CoV-2 S	4	8187.36	0.92
5000	SARS-CoV-2 S	4	3274.94	0.93
5000	SARS-CoV-2 S	4	1309.98	0.93
5000	SARS-CoV-2 S	4	523.99	0.98
5000	SARS-CoV-2 S	4	209.60	0.96
5000	SARS-CoV-2 S	4	83.84	0.99
5000	SARS-CoV-2 S	4	33.54	0.75
5000	SARS-CoV-2 S	4	13.41	0.40
5000	SARS-CoV-2 S	4	5.37	NE
50000	SARS-CoV-2 S	4	51171.00	0.87
50000	SARS-CoV-2 S	4	20468.40	0.89
50000	SARS-CoV-2 S	4	8187.36	0.87
50000	SARS-CoV-2 S	4	3274.94	0.93
50000	SARS-CoV-2 S	4	1309.98	0.97
50000	SARS-CoV-2 S	4	523.99	1.04
50000	SARS-CoV-2 S	4	209.60	0.83
50000	SARS-CoV-2 S	4	83.84	1.81
50000	SARS-CoV-2 S	4	33.54	NE
50000	SARS-CoV-2 S	4	13.41	0.40
50000	SARS-CoV-2 S	4	5.37	2.00

AU = arbitrary units; NE = not estimable; S = spike; SARS-CoV-2 = severe acute respiratory syndrome coronavirus 2.

(A) Spike antigen (continued)

Dilution	Antigen	Sample #	Expected concentration (AU/ml)	% Recovery
500	SARS-CoV-2 S	5	51171.00	1.05
500	SARS-CoV-2 S	5	20468.40	1.04
500	SARS-CoV-2 S	5	8187.36	1.00
500	SARS-CoV-2 S	5	3274.94	0.94
500	SARS-CoV-2 S	5	1309.98	0.95
500	SARS-CoV-2 S	5	523.99	0.91
500	SARS-CoV-2 S	5	209.60	0.94
500	SARS-CoV-2 S	5	83.84	0.94
500	SARS-CoV-2 S	5	33.54	0.92
500	SARS-CoV-2 S	5	13.41	0.89
500	SARS-CoV-2 S	5	5.37	0.75
5000	SARS-CoV-2 S	5	51171.00	0.97
5000	SARS-CoV-2 S	5	20468.40	0.90
5000	SARS-CoV-2 S	5	8187.36	0.88
5000	SARS-CoV-2 S	5	3274.94	0.84
5000	SARS-CoV-2 S	5	1309.98	0.90
5000	SARS-CoV-2 S	5	523.99	0.88
5000	SARS-CoV-2 S	5	209.60	0.88
5000	SARS-CoV-2 S	5	83.84	0.80
5000	SARS-CoV-2 S	5	33.54	0.48
5000	SARS-CoV-2 S	5	13.41	0.40
5000	SARS-CoV-2 S	5	5.37	NE
50000	SARS-CoV-2 S	5	51171.00	0.87
50000	SARS-CoV-2 S	5	20468.40	0.87
50000	SARS-CoV-2 S	5	8187.36	0.85
50000	SARS-CoV-2 S	5	3274.94	0.86
50000	SARS-CoV-2 S	5	1309.98	0.89
50000	SARS-CoV-2 S	5	523.99	0.64
50000	SARS-CoV-2 S	5	209.60	0.40
50000	SARS-CoV-2 S	5	83.84	NE
50000	SARS-CoV-2 S	5	33.54	NE
50000	SARS-CoV-2 S	5	13.41	NE
50000	SARS-CoV-2 S	5	5.37	NE
5000	SARS-CoV-2 S	6	51171.00	0.95
5000	SARS-CoV-2 S	6	20468.40	0.92
5000	SARS-CoV-2 S	6	8187.36	0.88
5000	SARS-CoV-2 S	6	3274.94	0.93
5000	SARS-CoV-2 S	6	1309.98	0.94
5000	SARS-CoV-2 S	6	523.99	0.93
5000	SARS-CoV-2 S	6	209.60	0.93
5000	SARS-CoV-2 S	6	83.84	0.78
5000	SARS-CoV-2 S	6	33.54	0.81
5000	SARS-CoV-2 S	6	13.41	NE
5000	SARS-CoV-2 S	6	5.37	NE
5000	SARS-CoV-2 S	7	51171.00	1.05
5000	SARS-CoV-2 S	7	20468.40	1.02

5000	SARS-CoV-2 S	7	8187.36	0.99
5000	SARS-CoV-2 S	7	3274.94	1.01
5000	SARS-CoV-2 S	7	1309.98	1.03
5000	SARS-CoV-2 S	7	523.99	1.07
5000	SARS-CoV-2 S	7	209.60	1.10
5000	SARS-CoV-2 S	7	83.84	1.10
5000	SARS-CoV-2 S	7	33.54	0.86
5000	SARS-CoV-2 S	7	13.41	1.04
5000	SARS-CoV-2 S	7	5.37	1.86
5000	SARS-CoV-2 S	8	51171.00	0.96
5000	SARS-CoV-2 S	8	20468.40	0.91
5000	SARS-CoV-2 S	8	8187.36	0.92
5000	SARS-CoV-2 S	8	3274.94	0.93
5000	SARS-CoV-2 S	8	1309.98	0.96
5000	SARS-CoV-2 S	8	523.99	0.99
5000	SARS-CoV-2 S	8	209.60	0.96
5000	SARS-CoV-2 S	8	83.84	1.05
5000	SARS-CoV-2 S	8	33.54	1.07
5000	SARS-CoV-2 S	8	13.41	0.97
5000	SARS-CoV-2 S	8	5.37	0.75

AU = arbitrary units; NE = not estimable; S = spike; SARS-CoV-2 = severe acute respiratory syndrome coronavirus 2.

(A) Spike antigen (continued)

Dilution	Antigen	Sample #	Expected concentration (AU/ml)	% Recovery
5000	SARS-CoV-2 S	9	51171.00	0.97
5000	SARS-CoV-2 S	9	20468.40	0.92
5000	SARS-CoV-2 S	9	8187.36	0.93
5000	SARS-CoV-2 S	9	3274.94	0.97
5000	SARS-CoV-2 S	9	1309.98	1.01
5000	SARS-CoV-2 S	9	523.99	1.03
5000	SARS-CoV-2 S	9	209.60	1.05
5000	SARS-CoV-2 S	9	83.84	1.03
5000	SARS-CoV-2 S	9	33.54	0.63
5000	SARS-CoV-2 S	9	13.41	0.40
5000	SARS-CoV-2 S	9	5.37	0.40
5000	SARS-CoV-2 S	10	51171.00	0.99
5000	SARS-CoV-2 S	10	20468.40	0.90
5000	SARS-CoV-2 S	10	8187.36	0.91
5000	SARS-CoV-2 S	10	3274.94	0.92
5000	SARS-CoV-2 S	10	1309.98	0.93
5000	SARS-CoV-2 S	10	523.99	1.02
5000	SARS-CoV-2 S	10	209.60	1.02
5000	SARS-CoV-2 S	10	83.84	1.00
5000	SARS-CoV-2 S	10	33.54	0.83
5000	SARS-CoV-2 S	10	13.41	0.40
5000	SARS-CoV-2 S	10	5.37	NE
500	SARS-CoV-2 S	11	51171.00	NE
500	SARS-CoV-2 S	11	20468.40	NE
500	SARS-CoV-2 S	11	8187.36	NE
500	SARS-CoV-2 S	11	3274.94	NE
500	SARS-CoV-2 S	11	1309.98	NE
500	SARS-CoV-2 S	11	523.99	NE
500	SARS-CoV-2 S	11	209.60	NE
500	SARS-CoV-2 S	11	83.84	NE
500	SARS-CoV-2 S	11	33.54	NE
500	SARS-CoV-2 S	11	13.41	NE
500	SARS-CoV-2 S	11	5.37	NE
5000	SARS-CoV-2 S	11	51171.00	NE
5000	SARS-CoV-2 S	11	20468.40	NE
5000	SARS-CoV-2 S	11	8187.36	NE
5000	SARS-CoV-2 S	11	3274.94	NE
5000	SARS-CoV-2 S	11	1309.98	NE
5000	SARS-CoV-2 S	11	523.99	NE
5000	SARS-CoV-2 S	11	209.60	NE
5000	SARS-CoV-2 S	11	83.84	NE
5000	SARS-CoV-2 S	11	33.54	NE
5000	SARS-CoV-2 S	11	13.41	NE
5000	SARS-CoV-2 S	11	5.37	NE
50000	SARS-CoV-2 S	11	51171.00	NE
50000	SARS-CoV-2 S	11	20468.40	NE

50000	SARS-CoV-2 S	11	8187.36	NE
50000	SARS-CoV-2 S	11	3274.94	NE
50000	SARS-CoV-2 S	11	1309.98	NE
50000	SARS-CoV-2 S	11	523.99	NE
50000	SARS-CoV-2 S	11	209.60	NE
50000	SARS-CoV-2 S	11	83.84	NE
50000	SARS-CoV-2 S	11	33.54	NE
50000	SARS-CoV-2 S	11	13.41	NE
50000	SARS-CoV-2 S	11	5.37	NE

AU = arbitrary units; NE = not estimable; S = spike; SARS-CoV-2 = severe acute respiratory syndrome coronavirus 2.

(A) Spike antigen (continued)

Dilution	Antigen	Sample #	Expected concentration (AU/ml)	% Recovery
500	SARS-CoV-2 S	12	51171.00	1.11
500	SARS-CoV-2 S	12	20468.40	1.12
500	SARS-CoV-2 S	12	8187.36	1.07
500	SARS-CoV-2 S	12	3274.94	1.01
500	SARS-CoV-2 S	12	1309.98	1.03
500	SARS-CoV-2 S	12	523.99	1.02
500	SARS-CoV-2 S	12	209.60	1.07
500	SARS-CoV-2 S	12	83.84	1.07
500	SARS-CoV-2 S	12	33.54	1.04
500	SARS-CoV-2 S	12	13.41	1.04
500	SARS-CoV-2 S	12	5.37	0.56
5000	SARS-CoV-2 S	12	51171.00	1.04
5000	SARS-CoV-2 S	12	20468.40	0.97
5000	SARS-CoV-2 S	12	8187.36	0.95
5000	SARS-CoV-2 S	12	3274.94	1.01
5000	SARS-CoV-2 S	12	1309.98	1.02
5000	SARS-CoV-2 S	12	523.99	1.00
5000	SARS-CoV-2 S	12	209.60	0.84
5000	SARS-CoV-2 S	12	83.84	0.69
5000	SARS-CoV-2 S	12	33.54	NE
5000	SARS-CoV-2 S	12	13.41	NE
5000	SARS-CoV-2 S	12	5.37	NE
50000	SARS-CoV-2 S	12	51171.00	0.93
50000	SARS-CoV-2 S	12	20468.40	0.90
50000	SARS-CoV-2 S	12	8187.36	0.93
50000	SARS-CoV-2 S	12	3274.94	0.97
50000	SARS-CoV-2 S	12	1309.98	0.97
50000	SARS-CoV-2 S	12	523.99	0.72
50000	SARS-CoV-2 S	12	209.60	0.40
50000	SARS-CoV-2 S	12	83.84	NE
50000	SARS-CoV-2 S	12	33.54	NE
50000	SARS-CoV-2 S	12	13.41	NE
50000	SARS-CoV-2 S	12	5.37	NE
500	SARS-CoV-2 S	13	51171.00	1.10
500	SARS-CoV-2 S	13	20468.40	1.11
500	SARS-CoV-2 S	13	8187.36	1.08
500	SARS-CoV-2 S	13	3274.94	1.09
500	SARS-CoV-2 S	13	1309.98	1.09
500	SARS-CoV-2 S	13	523.99	1.08
500	SARS-CoV-2 S	13	209.60	1.10
500	SARS-CoV-2 S	13	83.84	1.09
500	SARS-CoV-2 S	13	33.54	1.04
500	SARS-CoV-2 S	13	13.41	0.97
500	SARS-CoV-2 S	13	5.37	0.56
5000	SARS-CoV-2 S	13	51171.00	1.03
5000	SARS-CoV-2 S	13	20468.40	1.00

5000	SARS-CoV-2 S	13	8187.36	0.96
5000	SARS-CoV-2 S	13	3274.94	1.00
5000	SARS-CoV-2 S	13	1309.98	1.07
5000	SARS-CoV-2 S	13	523.99	1.02
5000	SARS-CoV-2 S	13	209.60	1.07
5000	SARS-CoV-2 S	13	83.84	0.97
5000	SARS-CoV-2 S	13	33.54	0.83
5000	SARS-CoV-2 S	13	13.41	0.67
5000	SARS-CoV-2 S	13	5.37	NE
50000	SARS-CoV-2 S	13	51171.00	0.93
50000	SARS-CoV-2 S	13	20468.40	0.94
50000	SARS-CoV-2 S	13	8187.36	0.95
50000	SARS-CoV-2 S	13	3274.94	0.97
50000	SARS-CoV-2 S	13	1309.98	0.95
50000	SARS-CoV-2 S	13	523.99	0.76
50000	SARS-CoV-2 S	13	209.60	0.86
50000	SARS-CoV-2 S	13	83.84	NE
50000	SARS-CoV-2 S	13	33.54	NE
50000	SARS-CoV-2 S	13	13.41	NE
50000	SARS-CoV-2 S	13	5.37	NE

AU = arbitrary units; NE = not estimable; S = spike; SARS-CoV-2 = severe acute respiratory syndrome coronavirus 2.

(A) Spike antigen (continued)

Dilution	Antigen	Sample #	Expected concentration (AU/ml)	% Recovery
500	SARS-CoV-2 S	14	51171.00	1.11
500	SARS-CoV-2 S	14	20468.40	1.13
500	SARS-CoV-2 S	14	8187.36	1.13
500	SARS-CoV-2 S	14	3274.94	1.02
500	SARS-CoV-2 S	14	1309.98	1.02
500	SARS-CoV-2 S	14	523.99	1.00
500	SARS-CoV-2 S	14	209.60	1.02
500	SARS-CoV-2 S	14	83.84	1.03
500	SARS-CoV-2 S	14	33.54	1.04
500	SARS-CoV-2 S	14	13.41	1.12
500	SARS-CoV-2 S	14	5.37	1.12
5000	SARS-CoV-2 S	14	51171.00	1.01
5000	SARS-CoV-2 S	14	20468.40	0.97
5000	SARS-CoV-2 S	14	8187.36	0.98
5000	SARS-CoV-2 S	14	3274.94	0.95
5000	SARS-CoV-2 S	14	1309.98	0.99
5000	SARS-CoV-2 S	14	523.99	0.99
5000	SARS-CoV-2 S	14	209.60	0.94
5000	SARS-CoV-2 S	14	83.84	0.93
5000	SARS-CoV-2 S	14	33.54	0.98
5000	SARS-CoV-2 S	14	13.41	0.75
5000	SARS-CoV-2 S	14	5.37	1.68
50000	SARS-CoV-2 S	14	51171.00	0.95
50000	SARS-CoV-2 S	14	20468.40	0.89
50000	SARS-CoV-2 S	14	8187.36	0.95
50000	SARS-CoV-2 S	14	3274.94	0.91
50000	SARS-CoV-2 S	14	1309.98	0.97
50000	SARS-CoV-2 S	14	523.99	1.07
50000	SARS-CoV-2 S	14	209.60	1.27
50000	SARS-CoV-2 S	14	83.84	2.00
50000	SARS-CoV-2 S	14	33.54	2.00
50000	SARS-CoV-2 S	14	13.41	2.00
50000	SARS-CoV-2 S	14	5.37	2.00
500	SARS-CoV-2 S	15	51171.00	1.04
500	SARS-CoV-2 S	15	20468.40	1.10
500	SARS-CoV-2 S	15	8187.36	1.08
500	SARS-CoV-2 S	15	3274.94	1.05
500	SARS-CoV-2 S	15	1309.98	1.04
500	SARS-CoV-2 S	15	523.99	1.04
500	SARS-CoV-2 S	15	209.60	1.04
500	SARS-CoV-2 S	15	83.84	1.05
500	SARS-CoV-2 S	15	33.54	1.07
500	SARS-CoV-2 S	15	13.41	0.97
500	SARS-CoV-2 S	15	5.37	1.12
5000	SARS-CoV-2 S	15	51171.00	0.99
5000	SARS-CoV-2 S	15	20468.40	1.00

5000	SARS-CoV-2 S	15	8187.36	0.96
5000	SARS-CoV-2 S	15	3274.94	0.93
5000	SARS-CoV-2 S	15	1309.98	0.96
5000	SARS-CoV-2 S	15	523.99	0.98
5000	SARS-CoV-2 S	15	209.60	1.03
5000	SARS-CoV-2 S	15	83.84	1.09
5000	SARS-CoV-2 S	15	33.54	1.25
5000	SARS-CoV-2 S	15	13.41	2.00
5000	SARS-CoV-2 S	15	5.37	2.00
50000	SARS-CoV-2 S	15	51171.00	0.88
50000	SARS-CoV-2 S	15	20468.40	0.90
50000	SARS-CoV-2 S	15	8187.36	0.95
50000	SARS-CoV-2 S	15	3274.94	1.04
50000	SARS-CoV-2 S	15	1309.98	1.10
50000	SARS-CoV-2 S	15	523.99	1.35
50000	SARS-CoV-2 S	15	209.60	1.37
50000	SARS-CoV-2 S	15	83.84	2.00
50000	SARS-CoV-2 S	15	33.54	2.00
50000	SARS-CoV-2 S	15	13.41	2.00
50000	SARS-CoV-2 S	15	5.37	2.00

AU = arbitrary units; S = spike; SARS-CoV-2 = severe acute respiratory syndrome coronavirus 2.

S4 Table. MSD ECL assay accuracy.

(B) Nucleocapsid antigen

Dilution	Antigen	Sample #	Expected concentration (AU/ml)	% Recovery
500	SARS-CoV-2 N	1	54748.00	1.13
500	SARS-CoV-2 N	1	21899.20	1.15
500	SARS-CoV-2 N	1	8759.68	1.10
500	SARS-CoV-2 N	1	3503.87	1.12
500	SARS-CoV-2 N	1	1401.55	1.09
500	SARS-CoV-2 N	1	560.62	1.09
500	SARS-CoV-2 N	1	224.25	1.08
500	SARS-CoV-2 N	1	89.70	1.08
500	SARS-CoV-2 N	1	35.88	0.98
500	SARS-CoV-2 N	1	14.35	0.77
500	SARS-CoV-2 N	1	5.74	0.40
5000	SARS-CoV-2 N	1	54748.00	1.07
5000	SARS-CoV-2 N	1	21899.20	0.99
5000	SARS-CoV-2 N	1	8759.68	0.91
5000	SARS-CoV-2 N	1	3503.87	0.90
5000	SARS-CoV-2 N	1	1401.55	0.90
5000	SARS-CoV-2 N	1	560.62	0.90
5000	SARS-CoV-2 N	1	224.25	0.85
5000	SARS-CoV-2 N	1	89.70	0.71
5000	SARS-CoV-2 N	1	35.88	0.42
5000	SARS-CoV-2 N	1	14.35	NE
5000	SARS-CoV-2 N	1	5.74	NE
50000	SARS-CoV-2 N	1	54748.00	0.89
50000	SARS-CoV-2 N	1	21899.20	0.86
50000	SARS-CoV-2 N	1	8759.68	0.85
50000	SARS-CoV-2 N	1	3503.87	0.87
50000	SARS-CoV-2 N	1	1401.55	0.86
50000	SARS-CoV-2 N	1	560.62	0.66
50000	SARS-CoV-2 N	1	224.25	0.40
50000	SARS-CoV-2 N	1	89.70	NE
50000	SARS-CoV-2 N	1	35.88	NE
50000	SARS-CoV-2 N	1	14.35	NE
50000	SARS-CoV-2 N	1	5.74	NE
500	SARS-CoV-2 N	2	54748.00	1.04
500	SARS-CoV-2 N	2	21899.20	1.04
500	SARS-CoV-2 N	2	8759.68	1.01
500	SARS-CoV-2 N	2	3503.87	1.01
500	SARS-CoV-2 N	2	1401.55	0.98
500	SARS-CoV-2 N	2	560.62	0.98
500	SARS-CoV-2 N	2	224.25	1.00
500	SARS-CoV-2 N	2	89.70	0.98
500	SARS-CoV-2 N	2	35.88	0.98

500	SARS-CoV-2 N	2	14.35	0.70
500	SARS-CoV-2 N	2	5.74	0.40
5000	SARS-CoV-2 N	2	54748.00	1.01
5000	SARS-CoV-2 N	2	21899.20	0.96
5000	SARS-CoV-2 N	2	8759.68	0.94
5000	SARS-CoV-2 N	2	3503.87	0.96
5000	SARS-CoV-2 N	2	1401.55	1.00
5000	SARS-CoV-2 N	2	560.62	0.98
5000	SARS-CoV-2 N	2	224.25	0.98
5000	SARS-CoV-2 N	2	89.70	0.75
5000	SARS-CoV-2 N	2	35.88	0.84
5000	SARS-CoV-2 N	2	14.35	NE
5000	SARS-CoV-2 N	2	5.74	NE
50000	SARS-CoV-2 N	2	54748.00	0.92
50000	SARS-CoV-2 N	2	21899.20	0.92
50000	SARS-CoV-2 N	2	8759.68	0.94
50000	SARS-CoV-2 N	2	3503.87	0.99
50000	SARS-CoV-2 N	2	1401.55	0.95
50000	SARS-CoV-2 N	2	560.62	0.79
50000	SARS-CoV-2 N	2	224.25	0.52
50000	SARS-CoV-2 N	2	89.70	NE
50000	SARS-CoV-2 N	2	35.88	NE
50000	SARS-CoV-2 N	2	14.35	NE
50000	SARS-CoV-2 N	2	5.74	NE

AU = arbitrary units; MSD ECL = multiplex electrochemiluminescence; N = nucleocapsid; NE = not estimable; SARS-CoV-2 = severe acute respiratory syndrome coronavirus 2.

(B) Nucleocapsid antigen (continued)

Dilution	Antigen	Sample #	Expected concentration (AU/ml)	% Recovery
500	SARS-CoV-2 N	3	54748.00	1.08
500	SARS-CoV-2 N	3	21899.20	1.10
500	SARS-CoV-2 N	3	8759.68	1.02
500	SARS-CoV-2 N	3	3503.87	1.11
500	SARS-CoV-2 N	3	1401.55	1.08
500	SARS-CoV-2 N	3	560.62	1.08
500	SARS-CoV-2 N	3	224.25	1.09
500	SARS-CoV-2 N	3	89.70	1.17
500	SARS-CoV-2 N	3	35.88	0.98
500	SARS-CoV-2 N	3	14.35	0.70
500	SARS-CoV-2 N	3	5.74	0.52
5000	SARS-CoV-2 N	3	54748.00	1.02
5000	SARS-CoV-2 N	3	21899.20	0.97
5000	SARS-CoV-2 N	3	8759.68	0.90
5000	SARS-CoV-2 N	3	3503.87	1.02
5000	SARS-CoV-2 N	3	1401.55	0.99
5000	SARS-CoV-2 N	3	560.62	1.02
5000	SARS-CoV-2 N	3	224.25	1.03
5000	SARS-CoV-2 N	3	89.70	0.80
5000	SARS-CoV-2 N	3	35.88	0.40
5000	SARS-CoV-2 N	3	14.35	NE
5000	SARS-CoV-2 N	3	5.74	NE
50000	SARS-CoV-2 N	3	54748.00	0.94
50000	SARS-CoV-2 N	3	21899.20	0.95
50000	SARS-CoV-2 N	3	8759.68	0.92
50000	SARS-CoV-2 N	3	3503.87	0.97
50000	SARS-CoV-2 N	3	1401.55	0.88
50000	SARS-CoV-2 N	3	560.62	0.54
50000	SARS-CoV-2 N	3	224.25	0.40
50000	SARS-CoV-2 N	3	89.70	NE
50000	SARS-CoV-2 N	3	35.88	NE
50000	SARS-CoV-2 N	3	14.35	NE
50000	SARS-CoV-2 N	3	5.74	NE
500	SARS-CoV-2 N	4	54748.00	1.08
500	SARS-CoV-2 N	4	21899.20	1.10
500	SARS-CoV-2 N	4	8759.68	1.04
500	SARS-CoV-2 N	4	3503.87	1.06
500	SARS-CoV-2 N	4	1401.55	1.05
500	SARS-CoV-2 N	4	560.62	1.04
500	SARS-CoV-2 N	4	224.25	1.05
500	SARS-CoV-2 N	4	89.70	1.05
500	SARS-CoV-2 N	4	35.88	0.95

500	SARS-CoV-2 N	4	14.35	0.84
500	SARS-CoV-2 N	4	5.74	0.52
5000	SARS-CoV-2 N	4	54748.00	0.94
5000	SARS-CoV-2 N	4	21899.20	0.89
5000	SARS-CoV-2 N	4	8759.68	0.87
5000	SARS-CoV-2 N	4	3503.87	0.92
5000	SARS-CoV-2 N	4	1401.55	0.92
5000	SARS-CoV-2 N	4	560.62	0.91
5000	SARS-CoV-2 N	4	224.25	0.88
5000	SARS-CoV-2 N	4	89.70	0.79
5000	SARS-CoV-2 N	4	35.88	0.40
5000	SARS-CoV-2 N	4	14.35	NE
5000	SARS-CoV-2 N	4	5.74	NE
50000	SARS-CoV-2 N	4	54748.00	0.85
50000	SARS-CoV-2 N	4	21899.20	0.84
50000	SARS-CoV-2 N	4	8759.68	0.83
50000	SARS-CoV-2 N	4	3503.87	0.89
50000	SARS-CoV-2 N	4	1401.55	0.80
50000	SARS-CoV-2 N	4	560.62	0.85
50000	SARS-CoV-2 N	4	224.25	0.83
50000	SARS-CoV-2 N	4	89.70	NE
50000	SARS-CoV-2 N	4	35.88	NE
50000	SARS-CoV-2 N	4	14.35	0.56
50000	SARS-CoV-2 N	4	5.74	NE

AU = arbitrary units; N = nucleocapsid; NE = not estimable; SARS-CoV-2 = severe acute respiratory syndrome coronavirus 2.

(B) Nucleocapsid antigen (continued)

Dilution	Antigen	Sample #	Expected concentration (AU/ml)	% Recovery
500	SARS-CoV-2 N	5	54748.00	1.05
500	SARS-CoV-2 N	5	21899.20	1.06
500	SARS-CoV-2 N	5	8759.68	0.98
500	SARS-CoV-2 N	5	3503.87	1.01
500	SARS-CoV-2 N	5	1401.55	0.94
500	SARS-CoV-2 N	5	560.62	0.94
500	SARS-CoV-2 N	5	224.25	0.92
500	SARS-CoV-2 N	5	89.70	1.01
500	SARS-CoV-2 N	5	35.88	0.84
500	SARS-CoV-2 N	5	14.35	0.77
500	SARS-CoV-2 N	5	5.74	0.40
5000	SARS-CoV-2 N	5	54748.00	0.98
5000	SARS-CoV-2 N	5	21899.20	0.91
5000	SARS-CoV-2 N	5	8759.68	0.82
5000	SARS-CoV-2 N	5	3503.87	0.92
5000	SARS-CoV-2 N	5	1401.55	0.93
5000	SARS-CoV-2 N	5	560.62	0.93
5000	SARS-CoV-2 N	5	224.25	0.84
5000	SARS-CoV-2 N	5	89.70	0.74
5000	SARS-CoV-2 N	5	35.88	0.40
5000	SARS-CoV-2 N	5	14.35	NE
5000	SARS-CoV-2 N	5	5.74	NE
50000	SARS-CoV-2 N	5	54748.00	0.85
50000	SARS-CoV-2 N	5	21899.20	0.85
50000	SARS-CoV-2 N	5	8759.68	0.82
50000	SARS-CoV-2 N	5	3503.87	0.93
50000	SARS-CoV-2 N	5	1401.55	0.91
50000	SARS-CoV-2 N	5	560.62	0.85
50000	SARS-CoV-2 N	5	224.25	0.40
50000	SARS-CoV-2 N	5	89.70	0.87
50000	SARS-CoV-2 N	5	35.88	NE
50000	SARS-CoV-2 N	5	14.35	NE
50000	SARS-CoV-2 N	5	5.74	NE
5000	SARS-CoV-2 N	6	54748.00	0.94
5000	SARS-CoV-2 N	6	21899.20	0.90
5000	SARS-CoV-2 N	6	8759.68	0.87
5000	SARS-CoV-2 N	6	3503.87	0.94
5000	SARS-CoV-2 N	6	1401.55	0.96
5000	SARS-CoV-2 N	6	560.62	0.92
5000	SARS-CoV-2 N	6	224.25	0.91
5000	SARS-CoV-2 N	6	89.70	0.76
5000	SARS-CoV-2 N	6	35.88	0.40

5000	SARS-CoV-2 N	6	14.35	NE
5000	SARS-CoV-2 N	6	5.74	NE
5000	SARS-CoV-2 N	7	54748.00	1.05
5000	SARS-CoV-2 N	7	21899.20	1.05
5000	SARS-CoV-2 N	7	8759.68	0.98
5000	SARS-CoV-2 N	7	3503.87	1.08
5000	SARS-CoV-2 N	7	1401.55	1.05
5000	SARS-CoV-2 N	7	560.62	1.05
5000	SARS-CoV-2 N	7	224.25	0.98
5000	SARS-CoV-2 N	7	89.70	0.87
5000	SARS-CoV-2 N	7	35.88	0.40
5000	SARS-CoV-2 N	7	14.35	NE
5000	SARS-CoV-2 N	7	5.74	NE
5000	SARS-CoV-2 N	8	54748.00	0.91
5000	SARS-CoV-2 N	8	21899.20	0.86
5000	SARS-CoV-2 N	8	8759.68	0.86
5000	SARS-CoV-2 N	8	3503.87	0.91
5000	SARS-CoV-2 N	8	1401.55	0.92
5000	SARS-CoV-2 N	8	560.62	0.88
5000	SARS-CoV-2 N	8	224.25	0.91
5000	SARS-CoV-2 N	8	89.70	0.82
5000	SARS-CoV-2 N	8	35.88	0.70
5000	SARS-CoV-2 N	8	14.35	NE
5000	SARS-CoV-2 N	8	5.74	NE

AU = arbitrary units; N = nucleocapsid; NE = not estimable; SARS-CoV-2 = severe acute respiratory syndrome coronavirus 2.

(B) Nucleocapsid antigen (continued)

Dilution	Antigen	Sample #	Expected concentration (AU/ml)	% Recovery
5000	SARS-CoV-2 N	9	54748.00	0.97
5000	SARS-CoV-2 N	9	21899.20	0.94
5000	SARS-CoV-2 N	9	8759.68	0.91
5000	SARS-CoV-2 N	9	3503.87	0.99
5000	SARS-CoV-2 N	9	1401.55	0.95
5000	SARS-CoV-2 N	9	560.62	0.95
5000	SARS-CoV-2 N	9	224.25	0.95
5000	SARS-CoV-2 N	9	89.70	0.84
5000	SARS-CoV-2 N	9	35.88	0.50
5000	SARS-CoV-2 N	9	14.35	NE
5000	SARS-CoV-2 N	9	5.74	NE
5000	SARS-CoV-2 N	10	54748.00	0.94
5000	SARS-CoV-2 N	10	21899.20	0.88
5000	SARS-CoV-2 N	10	8759.68	0.86
5000	SARS-CoV-2 N	10	3503.87	0.90
5000	SARS-CoV-2 N	10	1401.55	0.91
5000	SARS-CoV-2 N	10	560.62	0.94
5000	SARS-CoV-2 N	10	224.25	0.89
5000	SARS-CoV-2 N	10	89.70	0.79
5000	SARS-CoV-2 N	10	35.88	0.50
5000	SARS-CoV-2 N	10	14.35	NE
5000	SARS-CoV-2 N	10	5.74	NE
500	SARS-CoV-2 N	11	54748.00	NE
500	SARS-CoV-2 N	11	21899.20	NE
500	SARS-CoV-2 N	11	8759.68	NE
500	SARS-CoV-2 N	11	3503.87	NE
500	SARS-CoV-2 N	11	1401.55	NE
500	SARS-CoV-2 N	11	560.62	NE
500	SARS-CoV-2 N	11	224.25	NE
500	SARS-CoV-2 N	11	89.70	NE
500	SARS-CoV-2 N	11	35.88	NE
500	SARS-CoV-2 N	11	14.35	NE
500	SARS-CoV-2 N	11	5.74	NE
5000	SARS-CoV-2 N	11	54748.00	NE
5000	SARS-CoV-2 N	11	21899.20	NE
5000	SARS-CoV-2 N	11	8759.68	NE
5000	SARS-CoV-2 N	11	3503.87	NE
5000	SARS-CoV-2 N	11	1401.55	NE
5000	SARS-CoV-2 N	11	560.62	NE
5000	SARS-CoV-2 N	11	224.25	NE
5000	SARS-CoV-2 N	11	89.70	NE
5000	SARS-CoV-2 N	11	35.88	NE

5000	SARS-CoV-2 N	11	14.35	NE
5000	SARS-CoV-2 N	11	5.74	NE
50000	SARS-CoV-2 N	11	54748.00	NE
50000	SARS-CoV-2 N	11	21899.20	NE
50000	SARS-CoV-2 N	11	8759.68	NE
50000	SARS-CoV-2 N	11	3503.87	NE
50000	SARS-CoV-2 N	11	1401.55	NE
50000	SARS-CoV-2 N	11	560.62	NE
50000	SARS-CoV-2 N	11	224.25	NE
50000	SARS-CoV-2 N	11	89.70	NE
50000	SARS-CoV-2 N	11	35.88	NE
50000	SARS-CoV-2 N	11	14.35	NE
50000	SARS-CoV-2 N	11	5.74	NE

AU = arbitrary units; N = nucleocapsid; NE = not estimable; SARS-CoV-2 = severe acute respiratory syndrome coronavirus 2.

(B) Nucleocapsid antigen (continued)

Dilution	Antigen	Sample #	Expected concentration (AU/ml)	% Recovery
500	SARS-CoV-2 N	12	54748.00	1.11
500	SARS-CoV-2 N	12	21899.20	1.11
500	SARS-CoV-2 N	12	8759.68	1.04
500	SARS-CoV-2 N	12	3503.87	1.09
500	SARS-CoV-2 N	12	1401.55	1.05
500	SARS-CoV-2 N	12	560.62	1.06
500	SARS-CoV-2 N	12	224.25	1.06
500	SARS-CoV-2 N	12	89.70	1.08
500	SARS-CoV-2 N	12	35.88	1.00
500	SARS-CoV-2 N	12	14.35	0.84
500	SARS-CoV-2 N	12	5.74	0.40
5000	SARS-CoV-2 N	12	54748.00	1.04
5000	SARS-CoV-2 N	12	21899.20	0.97
5000	SARS-CoV-2 N	12	8759.68	0.93
5000	SARS-CoV-2 N	12	3503.87	1.04
5000	SARS-CoV-2 N	12	1401.55	1.01
5000	SARS-CoV-2 N	12	560.62	1.00
5000	SARS-CoV-2 N	12	224.25	0.93
5000	SARS-CoV-2 N	12	89.70	0.81
5000	SARS-CoV-2 N	12	35.88	0.40
5000	SARS-CoV-2 N	12	14.35	NE
5000	SARS-CoV-2 N	12	5.74	NE
50000	SARS-CoV-2 N	12	54748.00	0.92
50000	SARS-CoV-2 N	12	21899.20	0.91
50000	SARS-CoV-2 N	12	8759.68	0.84
50000	SARS-CoV-2 N	12	3503.87	0.93
50000	SARS-CoV-2 N	12	1401.55	0.90
50000	SARS-CoV-2 N	12	560.62	0.93
50000	SARS-CoV-2 N	12	224.25	0.40
50000	SARS-CoV-2 N	12	89.70	NE
50000	SARS-CoV-2 N	12	35.88	NE
50000	SARS-CoV-2 N	12	14.35	NE
50000	SARS-CoV-2 N	12	5.74	NE
500	SARS-CoV-2 N	13	54748.00	1.17
500	SARS-CoV-2 N	13	21899.20	1.11
500	SARS-CoV-2 N	13	8759.68	1.08
500	SARS-CoV-2 N	13	3503.87	1.08
500	SARS-CoV-2 N	13	1401.55	1.06
500	SARS-CoV-2 N	13	560.62	1.03
500	SARS-CoV-2 N	13	224.25	1.04
500	SARS-CoV-2 N	13	89.70	1.03
500	SARS-CoV-2 N	13	35.88	0.95

500	SARS-CoV-2 N	13	14.35	0.77
500	SARS-CoV-2 N	13	5.74	0.40
5000	SARS-CoV-2 N	13	54748.00	1.01
5000	SARS-CoV-2 N	13	21899.20	0.94
5000	SARS-CoV-2 N	13	8759.68	0.93
5000	SARS-CoV-2 N	13	3503.87	0.96
5000	SARS-CoV-2 N	13	1401.55	1.00
5000	SARS-CoV-2 N	13	560.62	0.96
5000	SARS-CoV-2 N	13	224.25	0.95
5000	SARS-CoV-2 N	13	89.70	0.78
5000	SARS-CoV-2 N	13	35.88	0.42
5000	SARS-CoV-2 N	13	14.35	NE
5000	SARS-CoV-2 N	13	5.74	NE
50000	SARS-CoV-2 N	13	54748.00	0.92
50000	SARS-CoV-2 N	13	21899.20	0.89
50000	SARS-CoV-2 N	13	8759.68	0.87
50000	SARS-CoV-2 N	13	3503.87	0.94
50000	SARS-CoV-2 N	13	1401.55	0.81
50000	SARS-CoV-2 N	13	560.62	0.47
50000	SARS-CoV-2 N	13	224.25	NE
50000	SARS-CoV-2 N	13	89.70	NE
50000	SARS-CoV-2 N	13	35.88	NE
50000	SARS-CoV-2 N	13	14.35	NE
50000	SARS-CoV-2 N	13	5.74	NE

AU = arbitrary units; N = nucleocapsid; NE = not estimable; SARS-CoV-2 = severe acute respiratory syndrome coronavirus 2.

(B) Nucleocapsid antigen (continued)

Dilution	Antigen	Sample #	Expected concentration (AU/ml)	% Recovery
500	SARS-CoV-2 N	14	54748.00	1.06
500	SARS-CoV-2 N	14	21899.20	1.08
500	SARS-CoV-2 N	14	8759.68	1.03
500	SARS-CoV-2 N	14	3503.87	1.08
500	SARS-CoV-2 N	14	1401.55	1.07
500	SARS-CoV-2 N	14	560.62	1.05
500	SARS-CoV-2 N	14	224.25	1.06
500	SARS-CoV-2 N	14	89.70	1.09
500	SARS-CoV-2 N	14	35.88	1.03
500	SARS-CoV-2 N	14	14.35	0.84
500	SARS-CoV-2 N	14	5.74	0.40
5000	SARS-CoV-2 N	14	54748.00	0.99
5000	SARS-CoV-2 N	14	21899.20	0.99
5000	SARS-CoV-2 N	14	8759.68	0.92
5000	SARS-CoV-2 N	14	3503.87	1.01
5000	SARS-CoV-2 N	14	1401.55	1.00
5000	SARS-CoV-2 N	14	560.62	0.99
5000	SARS-CoV-2 N	14	224.25	0.98
5000	SARS-CoV-2 N	14	89.70	0.79
5000	SARS-CoV-2 N	14	35.88	0.40
5000	SARS-CoV-2 N	14	14.35	NE
5000	SARS-CoV-2 N	14	5.74	NE
50000	SARS-CoV-2 N	14	54748.00	0.92
50000	SARS-CoV-2 N	14	21899.20	0.92
50000	SARS-CoV-2 N	14	8759.68	0.90
50000	SARS-CoV-2 N	14	3503.87	0.94
50000	SARS-CoV-2 N	14	1401.55	0.89
50000	SARS-CoV-2 N	14	560.62	0.64
50000	SARS-CoV-2 N	14	224.25	0.60
50000	SARS-CoV-2 N	14	89.70	0.57
50000	SARS-CoV-2 N	14	35.88	1.00
50000	SARS-CoV-2 N	14	14.35	NE
50000	SARS-CoV-2 N	14	5.74	NE
500	SARS-CoV-2 N	15	54748.00	1.04
500	SARS-CoV-2 N	15	21899.20	1.06
500	SARS-CoV-2 N	15	8759.68	1.03
500	SARS-CoV-2 N	15	3503.87	1.06
500	SARS-CoV-2 N	15	1401.55	1.05
500	SARS-CoV-2 N	15	560.62	1.06
500	SARS-CoV-2 N	15	224.25	1.04
500	SARS-CoV-2 N	15	89.70	1.03
500	SARS-CoV-2 N	15	35.88	1.00

500	SARS-CoV-2 N	15	14.35	0.77
500	SARS-CoV-2 N	15	5.74	0.40
5000	SARS-CoV-2 N	15	54748.00	0.93
5000	SARS-CoV-2 N	15	21899.20	0.89
5000	SARS-CoV-2 N	15	8759.68	0.89
5000	SARS-CoV-2 N	15	3503.87	0.93
5000	SARS-CoV-2 N	15	1401.55	0.95
5000	SARS-CoV-2 N	15	560.62	0.91
5000	SARS-CoV-2 N	15	224.25	0.87
5000	SARS-CoV-2 N	15	89.70	0.91
5000	SARS-CoV-2 N	15	35.88	0.81
5000	SARS-CoV-2 N	15	14.35	NE
5000	SARS-CoV-2 N	15	5.74	NE
50000	SARS-CoV-2 N	15	54748.00	0.83
50000	SARS-CoV-2 N	15	21899.20	0.86
50000	SARS-CoV-2 N	15	8759.68	0.87
50000	SARS-CoV-2 N	15	3503.87	0.93
50000	SARS-CoV-2 N	15	1401.55	0.93
50000	SARS-CoV-2 N	15	560.62	0.90
50000	SARS-CoV-2 N	15	224.25	0.82
50000	SARS-CoV-2 N	15	89.70	NE
50000	SARS-CoV-2 N	15	35.88	NE
50000	SARS-CoV-2 N	15	14.35	NE
50000	SARS-CoV-2 N	15	5.74	NE

AU = arbitrary units; N = nucleocapsid; NE = not estimable; SARS-CoV-2 = severe acute respiratory syndrome coronavirus 2.

S4 Table. MSD ECL assay accuracy.

(C) Receptor-binding domain antigen

Dilution	Antigen	Sample #	Expected concentration (AU/ml)	% Recovery
500	SARS-CoV-2 RBD	1	10802.00	1.12
500	SARS-CoV-2 RBD	1	4320.80	1.13
500	SARS-CoV-2 RBD	1	1728.32	1.11
500	SARS-CoV-2 RBD	1	691.33	1.13
500	SARS-CoV-2 RBD	1	276.53	1.08
500	SARS-CoV-2 RBD	1	110.61	1.14
500	SARS-CoV-2 RBD	1	44.24	1.11
500	SARS-CoV-2 RBD	1	17.70	1.07
500	SARS-CoV-2 RBD	1	7.08	0.71
500	SARS-CoV-2 RBD	1	2.83	0.40
500	SARS-CoV-2 RBD	1	1.13	NE
5000	SARS-CoV-2 RBD	1	10802.00	1.11
5000	SARS-CoV-2 RBD	1	4320.80	1.04
5000	SARS-CoV-2 RBD	1	1728.32	0.96
5000	SARS-CoV-2 RBD	1	691.33	0.85
5000	SARS-CoV-2 RBD	1	276.53	0.92
5000	SARS-CoV-2 RBD	1	110.61	0.99
5000	SARS-CoV-2 RBD	1	44.24	1.02
5000	SARS-CoV-2 RBD	1	17.70	1.30
5000	SARS-CoV-2 RBD	1	7.08	1.13
5000	SARS-CoV-2 RBD	1	2.83	2.00
5000	SARS-CoV-2 RBD	1	1.13	2.00
50000	SARS-CoV-2 RBD	1	10802.00	0.91
50000	SARS-CoV-2 RBD	1	4320.80	0.95
50000	SARS-CoV-2 RBD	1	1728.32	0.80
50000	SARS-CoV-2 RBD	1	691.33	1.06
50000	SARS-CoV-2 RBD	1	276.53	1.01
50000	SARS-CoV-2 RBD	1	110.61	0.90
50000	SARS-CoV-2 RBD	1	44.24	2.00
50000	SARS-CoV-2 RBD	1	17.70	NE
50000	SARS-CoV-2 RBD	1	7.08	NE
50000	SARS-CoV-2 RBD	1	2.83	NE
50000	SARS-CoV-2 RBD	1	1.13	2.00
500	SARS-CoV-2 RBD	2	10802.00	1.24
500	SARS-CoV-2 RBD	2	4320.80	1.10
500	SARS-CoV-2 RBD	2	1728.32	1.03
500	SARS-CoV-2 RBD	2	691.33	0.88
500	SARS-CoV-2 RBD	2	276.53	0.99
500	SARS-CoV-2 RBD	2	110.61	0.94
500	SARS-CoV-2 RBD	2	44.24	1.06
500	SARS-CoV-2 RBD	2	17.70	1.02
500	SARS-CoV-2 RBD	2	7.08	0.85
500	SARS-CoV-2 RBD	2	2.83	0.71
500	SARS-CoV-2 RBD	2	1.13	NE
5000	SARS-CoV-2 RBD	2	10802.00	1.08
5000	SARS-CoV-2 RBD	2	4320.80	0.99

5000	SARS-CoV-2 RBD	2	1728.32	1.03
5000	SARS-CoV-2 RBD	2	691.33	1.04
5000	SARS-CoV-2 RBD	2	276.53	1.12
5000	SARS-CoV-2 RBD	2	110.61	1.22
5000	SARS-CoV-2 RBD	2	44.24	1.36
5000	SARS-CoV-2 RBD	2	17.70	1.30
5000	SARS-CoV-2 RBD	2	7.08	1.55
5000	SARS-CoV-2 RBD	2	2.83	1.77
5000	SARS-CoV-2 RBD	2	1.13	NE
50000	SARS-CoV-2 RBD	2	10802.00	1.08
50000	SARS-CoV-2 RBD	2	4320.80	1.13
50000	SARS-CoV-2 RBD	2	1728.32	1.11
50000	SARS-CoV-2 RBD	2	691.33	1.21
50000	SARS-CoV-2 RBD	2	276.53	0.89
50000	SARS-CoV-2 RBD	2	110.61	0.90
50000	SARS-CoV-2 RBD	2	44.24	0.47
50000	SARS-CoV-2 RBD	2	17.70	NE
50000	SARS-CoV-2 RBD	2	7.08	NE
50000	SARS-CoV-2 RBD	2	2.83	NE
50000	SARS-CoV-2 RBD	2	1.13	NE

AU = arbitrary units; MSD ECL = multiplex electrochemiluminescence; NE = not estimable;
RBD = receptor-binding domain; SARS-CoV-2 = severe acute respiratory syndrome coronavirus 2.

(C) Receptor-binding domain antigen (continued)

Dilution	Antigen	Sample #	Expected concentration (AU/ml)	% Recovery
500	SARS-CoV-2 RBD	3	10802.00	1.12
500	SARS-CoV-2 RBD	3	4320.80	1.31
500	SARS-CoV-2 RBD	3	1728.32	1.10
500	SARS-CoV-2 RBD	3	691.33	1.05
500	SARS-CoV-2 RBD	3	276.53	0.95
500	SARS-CoV-2 RBD	3	110.61	1.13
500	SARS-CoV-2 RBD	3	44.24	1.06
500	SARS-CoV-2 RBD	3	17.70	1.19
500	SARS-CoV-2 RBD	3	7.08	0.85
500	SARS-CoV-2 RBD	3	2.83	0.71
500	SARS-CoV-2 RBD	3	1.13	NE
5000	SARS-CoV-2 RBD	3	10802.00	1.09
5000	SARS-CoV-2 RBD	3	4320.80	1.09
5000	SARS-CoV-2 RBD	3	1728.32	1.07
5000	SARS-CoV-2 RBD	3	691.33	1.13
5000	SARS-CoV-2 RBD	3	276.53	1.11
5000	SARS-CoV-2 RBD	3	110.61	1.21
5000	SARS-CoV-2 RBD	3	44.24	1.45
5000	SARS-CoV-2 RBD	3	17.70	1.30
5000	SARS-CoV-2 RBD	3	7.08	1.27
5000	SARS-CoV-2 RBD	3	2.83	1.41
5000	SARS-CoV-2 RBD	3	1.13	2.00
50000	SARS-CoV-2 RBD	3	10802.00	0.78
50000	SARS-CoV-2 RBD	3	4320.80	0.88
50000	SARS-CoV-2 RBD	3	1728.32	0.81
50000	SARS-CoV-2 RBD	3	691.33	0.99
50000	SARS-CoV-2 RBD	3	276.53	1.26
50000	SARS-CoV-2 RBD	3	110.61	2.00
50000	SARS-CoV-2 RBD	3	44.24	2.00
50000	SARS-CoV-2 RBD	3	17.70	NE
50000	SARS-CoV-2 RBD	3	7.08	NE
50000	SARS-CoV-2 RBD	3	2.83	NE
50000	SARS-CoV-2 RBD	3	1.13	NE
500	SARS-CoV-2 RBD	4	10802.00	1.34
500	SARS-CoV-2 RBD	4	4320.80	1.35
500	SARS-CoV-2 RBD	4	1728.32	1.23
500	SARS-CoV-2 RBD	4	691.33	1.34
500	SARS-CoV-2 RBD	4	276.53	1.30
500	SARS-CoV-2 RBD	4	110.61	1.29
500	SARS-CoV-2 RBD	4	44.24	1.22
500	SARS-CoV-2 RBD	4	17.70	1.19
500	SARS-CoV-2 RBD	4	7.08	0.99
500	SARS-CoV-2 RBD	4	2.83	0.71
500	SARS-CoV-2 RBD	4	1.13	0.88
5000	SARS-CoV-2 RBD	4	10802.00	0.97
5000	SARS-CoV-2 RBD	4	4320.80	0.95

5000	SARS-CoV-2 RBD	4	1728.32	1.01
5000	SARS-CoV-2 RBD	4	691.33	0.96
5000	SARS-CoV-2 RBD	4	276.53	1.02
5000	SARS-CoV-2 RBD	4	110.61	1.08
5000	SARS-CoV-2 RBD	4	44.24	1.15
5000	SARS-CoV-2 RBD	4	17.70	1.19
5000	SARS-CoV-2 RBD	4	7.08	1.98
5000	SARS-CoV-2 RBD	4	2.83	0.40
5000	SARS-CoV-2 RBD	4	1.13	NE
50000	SARS-CoV-2 RBD	4	10802.00	0.88
50000	SARS-CoV-2 RBD	4	4320.80	0.84
50000	SARS-CoV-2 RBD	4	1728.32	0.92
50000	SARS-CoV-2 RBD	4	691.33	1.12
50000	SARS-CoV-2 RBD	4	276.53	1.14
50000	SARS-CoV-2 RBD	4	110.61	1.54
50000	SARS-CoV-2 RBD	4	44.24	2.00
50000	SARS-CoV-2 RBD	4	17.70	2.00
50000	SARS-CoV-2 RBD	4	7.08	NE
50000	SARS-CoV-2 RBD	4	2.83	NE
50000	SARS-CoV-2 RBD	4	1.13	NE

AU = arbitrary units; MSD ECL = multiplex electrochemiluminescence; NE = not estimable; RBD = receptor-binding domain; SARS-CoV-2 = severe acute respiratory syndrome coronavirus 2.

(C) Receptor-binding domain antigen (continued)

Dilution	Antigen	Sample #	Expected concentration (AU/ml)	% Recovery
500	SARS-CoV-2 RBD	5	10802.00	NE
500	SARS-CoV-2 RBD	5	4320.80	NE
500	SARS-CoV-2 RBD	5	1728.32	NE
500	SARS-CoV-2 RBD	5	691.33	NE
500	SARS-CoV-2 RBD	5	276.53	NE
500	SARS-CoV-2 RBD	5	110.61	NE
500	SARS-CoV-2 RBD	5	44.24	NE
500	SARS-CoV-2 RBD	5	17.70	NE
500	SARS-CoV-2 RBD	5	7.08	NE
500	SARS-CoV-2 RBD	5	2.83	NE
500	SARS-CoV-2 RBD	5	1.13	NE
5000	SARS-CoV-2 RBD	5	10802.00	0.95
5000	SARS-CoV-2 RBD	5	4320.80	0.93
5000	SARS-CoV-2 RBD	5	1728.32	0.89
5000	SARS-CoV-2 RBD	5	691.33	1.03
5000	SARS-CoV-2 RBD	5	276.53	1.03
5000	SARS-CoV-2 RBD	5	110.61	1.03
5000	SARS-CoV-2 RBD	5	44.24	1.08
5000	SARS-CoV-2 RBD	5	17.70	0.57
5000	SARS-CoV-2 RBD	5	7.08	0.40
5000	SARS-CoV-2 RBD	5	2.83	2.00
5000	SARS-CoV-2 RBD	5	1.13	NE
50000	SARS-CoV-2 RBD	5	10802.00	0.87
50000	SARS-CoV-2 RBD	5	4320.80	0.84
50000	SARS-CoV-2 RBD	5	1728.32	0.85
50000	SARS-CoV-2 RBD	5	691.33	1.01
50000	SARS-CoV-2 RBD	5	276.53	1.49
50000	SARS-CoV-2 RBD	5	110.61	2.00
50000	SARS-CoV-2 RBD	5	44.24	2.00
50000	SARS-CoV-2 RBD	5	17.70	2.00
50000	SARS-CoV-2 RBD	5	7.08	2.00
50000	SARS-CoV-2 RBD	5	2.83	2.00
50000	SARS-CoV-2 RBD	5	1.13	2.00
5000	SARS-CoV-2 RBD	6	10802.00	0.99
5000	SARS-CoV-2 RBD	6	4320.80	1.03
5000	SARS-CoV-2 RBD	6	1728.32	1.03
5000	SARS-CoV-2 RBD	6	691.33	1.09
5000	SARS-CoV-2 RBD	6	276.53	1.11
5000	SARS-CoV-2 RBD	6	110.61	1.10
5000	SARS-CoV-2 RBD	6	44.24	1.04
5000	SARS-CoV-2 RBD	6	17.70	0.40
5000	SARS-CoV-2 RBD	6	7.08	NE
5000	SARS-CoV-2 RBD	6	2.83	NE
5000	SARS-CoV-2 RBD	6	1.13	NE
5000	SARS-CoV-2 RBD	7	10802.00	0.92
5000	SARS-CoV-2 RBD	7	4320.80	1.14

5000	SARS-CoV-2 RBD	7	1728.32	0.98
5000	SARS-CoV-2 RBD	7	691.33	1.03
5000	SARS-CoV-2 RBD	7	276.53	0.90
5000	SARS-CoV-2 RBD	7	110.61	1.07
5000	SARS-CoV-2 RBD	7	44.24	0.79
5000	SARS-CoV-2 RBD	7	17.70	0.40
5000	SARS-CoV-2 RBD	7	7.08	NE
5000	SARS-CoV-2 RBD	7	2.83	NE
5000	SARS-CoV-2 RBD	7	1.13	NE
5000	SARS-CoV-2 RBD	8	10802.00	0.91
5000	SARS-CoV-2 RBD	8	4320.80	0.92
5000	SARS-CoV-2 RBD	8	1728.32	0.85
5000	SARS-CoV-2 RBD	8	691.33	0.89
5000	SARS-CoV-2 RBD	8	276.53	0.92
5000	SARS-CoV-2 RBD	8	110.61	0.98
5000	SARS-CoV-2 RBD	8	44.24	0.93
5000	SARS-CoV-2 RBD	8	17.70	0.90
5000	SARS-CoV-2 RBD	8	7.08	0.42
5000	SARS-CoV-2 RBD	8	2.83	2.00
5000	SARS-CoV-2 RBD	8	1.13	1.77

AU = arbitrary units; MSD ECL = multiplex electrochemiluminescence; NE = not estimable; RBD = receptor-binding domain; SARS-CoV-2 = severe acute respiratory syndrome coronavirus 2.

(C) Receptor-binding domain antigen (continued)

Dilution	Antigen	Sample #	Expected concentration (AU/ml)	% Recovery
5000	SARS-CoV-2 RBD	9	10802.00	0.94
5000	SARS-CoV-2 RBD	9	4320.80	1.01
5000	SARS-CoV-2 RBD	9	1728.32	0.88
5000	SARS-CoV-2 RBD	9	691.33	1.05
5000	SARS-CoV-2 RBD	9	276.53	0.89
5000	SARS-CoV-2 RBD	9	110.61	1.08
5000	SARS-CoV-2 RBD	9	44.24	1.11
5000	SARS-CoV-2 RBD	9	17.70	1.13
5000	SARS-CoV-2 RBD	9	7.08	1.27
5000	SARS-CoV-2 RBD	9	2.83	1.06
5000	SARS-CoV-2 RBD	9	1.13	0.88
5000	SARS-CoV-2 RBD	10	10802.00	0.96
5000	SARS-CoV-2 RBD	10	4320.80	0.92
5000	SARS-CoV-2 RBD	10	1728.32	0.91
5000	SARS-CoV-2 RBD	10	691.33	0.96
5000	SARS-CoV-2 RBD	10	276.53	0.98
5000	SARS-CoV-2 RBD	10	110.61	1.00
5000	SARS-CoV-2 RBD	10	44.24	0.99
5000	SARS-CoV-2 RBD	10	17.70	1.02
5000	SARS-CoV-2 RBD	10	7.08	1.70
5000	SARS-CoV-2 RBD	10	2.83	NE
5000	SARS-CoV-2 RBD	10	1.13	1.77
500	SARS-CoV-2 RBD	11	10802.00	NE
500	SARS-CoV-2 RBD	11	4320.80	NE
500	SARS-CoV-2 RBD	11	1728.32	NE
500	SARS-CoV-2 RBD	11	691.33	NE
500	SARS-CoV-2 RBD	11	276.53	NE
500	SARS-CoV-2 RBD	11	110.61	NE
500	SARS-CoV-2 RBD	11	44.24	NE
500	SARS-CoV-2 RBD	11	17.70	NE
500	SARS-CoV-2 RBD	11	7.08	NE
500	SARS-CoV-2 RBD	11	2.83	NE
500	SARS-CoV-2 RBD	11	1.13	NE
5000	SARS-CoV-2 RBD	11	10802.00	NE
5000	SARS-CoV-2 RBD	11	4320.80	NE
5000	SARS-CoV-2 RBD	11	1728.32	NE
5000	SARS-CoV-2 RBD	11	691.33	NE
5000	SARS-CoV-2 RBD	11	276.53	NE
5000	SARS-CoV-2 RBD	11	110.61	NE
5000	SARS-CoV-2 RBD	11	44.24	NE
5000	SARS-CoV-2 RBD	11	17.70	NE
5000	SARS-CoV-2 RBD	11	7.08	NE
5000	SARS-CoV-2 RBD	11	2.83	NE
5000	SARS-CoV-2 RBD	11	1.13	NE
50000	SARS-CoV-2 RBD	11	10802.00	NE
50000	SARS-CoV-2 RBD	11	4320.80	NE

50000	SARS-CoV-2 RBD	11	1728.32	NE
50000	SARS-CoV-2 RBD	11	691.33	NE
50000	SARS-CoV-2 RBD	11	276.53	NE
50000	SARS-CoV-2 RBD	11	110.61	NE
50000	SARS-CoV-2 RBD	11	44.24	NE
50000	SARS-CoV-2 RBD	11	17.70	NE
50000	SARS-CoV-2 RBD	11	7.08	NE
50000	SARS-CoV-2 RBD	11	2.83	NE
50000	SARS-CoV-2 RBD	11	1.13	NE

AU = arbitrary units; MSD ECL = multiplex electrochemiluminescence; NE = not estimable; RBD = receptor-binding domain; SARS-CoV-2 = severe acute respiratory syndrome coronavirus 2.

(C) Receptor-binding domain antigen (continued)

Dilution	Antigen	Sample #	Expected concentration (AU/ml)	% Recovery
500	SARS-CoV-2 RBD	12	10802.00	1.25
500	SARS-CoV-2 RBD	12	4320.80	1.15
500	SARS-CoV-2 RBD	12	1728.32	1.02
500	SARS-CoV-2 RBD	12	691.33	1.15
500	SARS-CoV-2 RBD	12	276.53	1.11
500	SARS-CoV-2 RBD	12	110.61	1.13
500	SARS-CoV-2 RBD	12	44.24	1.08
500	SARS-CoV-2 RBD	12	17.70	1.02
500	SARS-CoV-2 RBD	12	7.08	0.85
500	SARS-CoV-2 RBD	12	2.83	0.71
500	SARS-CoV-2 RBD	12	1.13	NE
5000	SARS-CoV-2 RBD	12	10802.00	1.03
5000	SARS-CoV-2 RBD	12	4320.80	1.03
5000	SARS-CoV-2 RBD	12	1728.32	0.95
5000	SARS-CoV-2 RBD	12	691.33	1.12
5000	SARS-CoV-2 RBD	12	276.53	1.08
5000	SARS-CoV-2 RBD	12	110.61	1.07
5000	SARS-CoV-2 RBD	12	44.24	0.99
5000	SARS-CoV-2 RBD	12	17.70	0.40
5000	SARS-CoV-2 RBD	12	7.08	NE
5000	SARS-CoV-2 RBD	12	2.83	NE
5000	SARS-CoV-2 RBD	12	1.13	NE
50000	SARS-CoV-2 RBD	12	10802.00	0.88
50000	SARS-CoV-2 RBD	12	4320.80	0.94
50000	SARS-CoV-2 RBD	12	1728.32	0.80
50000	SARS-CoV-2 RBD	12	691.33	0.96
50000	SARS-CoV-2 RBD	12	276.53	1.29
50000	SARS-CoV-2 RBD	12	110.61	1.90
50000	SARS-CoV-2 RBD	12	44.24	2.00
50000	SARS-CoV-2 RBD	12	17.70	NE
50000	SARS-CoV-2 RBD	12	7.08	0.40
50000	SARS-CoV-2 RBD	12	2.83	NE
50000	SARS-CoV-2 RBD	12	1.13	NE
500	SARS-CoV-2 RBD	13	10802.00	1.20
500	SARS-CoV-2 RBD	13	4320.80	1.11
500	SARS-CoV-2 RBD	13	1728.32	1.00
500	SARS-CoV-2 RBD	13	691.33	1.07
500	SARS-CoV-2 RBD	13	276.53	1.10
500	SARS-CoV-2 RBD	13	110.61	1.10
500	SARS-CoV-2 RBD	13	44.24	1.06
500	SARS-CoV-2 RBD	13	17.70	1.02
500	SARS-CoV-2 RBD	13	7.08	0.71
500	SARS-CoV-2 RBD	13	2.83	0.71
500	SARS-CoV-2 RBD	13	1.13	NE
5000	SARS-CoV-2 RBD	13	10802.00	1.04
5000	SARS-CoV-2 RBD	13	4320.80	1.02

5000	SARS-CoV-2 RBD	13	1728.32	1.02
5000	SARS-CoV-2 RBD	13	691.33	1.18
5000	SARS-CoV-2 RBD	13	276.53	1.25
5000	SARS-CoV-2 RBD	13	110.61	1.26
5000	SARS-CoV-2 RBD	13	44.24	1.20
5000	SARS-CoV-2 RBD	13	17.70	0.57
5000	SARS-CoV-2 RBD	13	7.08	0.40
5000	SARS-CoV-2 RBD	13	2.83	NE
5000	SARS-CoV-2 RBD	13	1.13	NE
50000	SARS-CoV-2 RBD	13	10802.00	0.93
50000	SARS-CoV-2 RBD	13	4320.80	0.92
50000	SARS-CoV-2 RBD	13	1728.32	0.96
50000	SARS-CoV-2 RBD	13	691.33	1.01
50000	SARS-CoV-2 RBD	13	276.53	0.84
50000	SARS-CoV-2 RBD	13	110.61	0.40
50000	SARS-CoV-2 RBD	13	44.24	0.75
50000	SARS-CoV-2 RBD	13	17.70	NE
50000	SARS-CoV-2 RBD	13	7.08	NE
50000	SARS-CoV-2 RBD	13	2.83	NE
50000	SARS-CoV-2 RBD	13	1.13	NE

AU = arbitrary units; MSD ECL = multiplex electrochemiluminescence; NE = not estimable; RBD = receptor-binding domain; SARS-CoV-2 = severe acute respiratory syndrome coronavirus 2.

(C) Receptor-binding domain antigen (continued)

Dilution	Antigen	Sample #	Expected concentration (AU/ml)	% Recovery
500	SARS-CoV-2 RBD	14	10802.00	1.15
500	SARS-CoV-2 RBD	14	4320.80	1.14
500	SARS-CoV-2 RBD	14	1728.32	1.13
500	SARS-CoV-2 RBD	14	691.33	1.28
500	SARS-CoV-2 RBD	14	276.53	1.28
500	SARS-CoV-2 RBD	14	110.61	1.54
500	SARS-CoV-2 RBD	14	44.24	1.24
500	SARS-CoV-2 RBD	14	17.70	1.19
500	SARS-CoV-2 RBD	14	7.08	1.13
500	SARS-CoV-2 RBD	14	2.83	0.71
500	SARS-CoV-2 RBD	14	1.13	NE
5000	SARS-CoV-2 RBD	14	10802.00	0.93
5000	SARS-CoV-2 RBD	14	4320.80	1.03
5000	SARS-CoV-2 RBD	14	1728.32	0.92
5000	SARS-CoV-2 RBD	14	691.33	1.06
5000	SARS-CoV-2 RBD	14	276.53	0.99
5000	SARS-CoV-2 RBD	14	110.61	1.09
5000	SARS-CoV-2 RBD	14	44.24	0.99
5000	SARS-CoV-2 RBD	14	17.70	0.40
5000	SARS-CoV-2 RBD	14	7.08	NE
5000	SARS-CoV-2 RBD	14	2.83	NE
5000	SARS-CoV-2 RBD	14	1.13	NE
50000	SARS-CoV-2 RBD	14	10802.00	1.09
50000	SARS-CoV-2 RBD	14	4320.80	1.10
50000	SARS-CoV-2 RBD	14	1728.32	1.11
50000	SARS-CoV-2 RBD	14	691.33	1.23
50000	SARS-CoV-2 RBD	14	276.53	1.39
50000	SARS-CoV-2 RBD	14	110.61	1.65
50000	SARS-CoV-2 RBD	14	44.24	2.00
50000	SARS-CoV-2 RBD	14	17.70	1.36
50000	SARS-CoV-2 RBD	14	7.08	0.71
50000	SARS-CoV-2 RBD	14	2.83	2.00
50000	SARS-CoV-2 RBD	14	1.13	NE
500	SARS-CoV-2 RBD	15	10802.00	1.21
500	SARS-CoV-2 RBD	15	4320.80	1.21
500	SARS-CoV-2 RBD	15	1728.32	1.10
500	SARS-CoV-2 RBD	15	691.33	1.03
500	SARS-CoV-2 RBD	15	276.53	1.08
500	SARS-CoV-2 RBD	15	110.61	1.11
500	SARS-CoV-2 RBD	15	44.24	1.13
500	SARS-CoV-2 RBD	15	17.70	1.07
500	SARS-CoV-2 RBD	15	7.08	0.85
500	SARS-CoV-2 RBD	15	2.83	0.40
500	SARS-CoV-2 RBD	15	1.13	NE
5000	SARS-CoV-2 RBD	15	10802.00	1.04
5000	SARS-CoV-2 RBD	15	4320.80	1.07

5000	SARS-CoV-2 RBD	15	1728.32	1.16
5000	SARS-CoV-2 RBD	15	691.33	1.04
5000	SARS-CoV-2 RBD	15	276.53	1.09
5000	SARS-CoV-2 RBD	15	110.61	1.13
5000	SARS-CoV-2 RBD	15	44.24	1.04
5000	SARS-CoV-2 RBD	15	17.70	1.24
5000	SARS-CoV-2 RBD	15	7.08	1.13
5000	SARS-CoV-2 RBD	15	2.83	2.00
5000	SARS-CoV-2 RBD	15	1.13	2.00
50000	SARS-CoV-2 RBD	15	10802.00	0.92
50000	SARS-CoV-2 RBD	15	4320.80	1.01
50000	SARS-CoV-2 RBD	15	1728.32	0.90
50000	SARS-CoV-2 RBD	15	691.33	1.17
50000	SARS-CoV-2 RBD	15	276.53	1.53
50000	SARS-CoV-2 RBD	15	110.61	2.00
50000	SARS-CoV-2 RBD	15	44.24	2.00
50000	SARS-CoV-2 RBD	15	17.70	2.00
50000	SARS-CoV-2 RBD	15	7.08	2.00
50000	SARS-CoV-2 RBD	15	2.83	2.00
50000	SARS-CoV-2 RBD	15	1.13	2.00

AU = arbitrary units; MSD ECL = multiplex electrochemiluminescence; NE = not estimable; RBD = receptor-binding domain; SARS-CoV-2 = severe acute respiratory syndrome coronavirus 2.
