# Supplementary material for: Robust validation and performance comparison of immunogenicity assays assessing IgG and neutralizing antibodies to SARS-CoV-2
Source: PLoS One. 2022 Feb 7;17(2):e0262922. doi: 10.1371/journal.pone.0262922 (PMC8820625; doi:10.1371/journal.pone.0262922)
Supplement: S5 Table — Correlation between the results of the MNT and MSD ECL assays: (A) spike, (B) nucleocapsid, and (C) receptor-binding domain antigens. Ab[C] = antibody concentration; MNT = microneutralization; MSD ECL = multiplex electrochemiluminescence; N = nucleocapsid; NE = not estimable; RBD = receptor-binding domain; S = spike; SARS-CoV-2 = severe acute respiratory syndrome coronavirus 2. (PDF) [file pone.0262922.s006.pdf]

S5 Table. Correlation between the results of the MNT and MSD ECL assays

(A) Spike antigen

Sample #	MSD Antigen	Status	MNT Ab[C] (AU/ml)	MSD ECL Ab[C] (AU/ml)
1	S	Vaccinated / clinical trial	NE	77833
2	S	Vaccinated / clinical trial	50	15620
3	S	Vaccinated / clinical trial	2310	541598
4	S	Vaccinated / clinical trial	NE	245554
5	S	Vaccinated / clinical trial	119	107396
6	S	Vaccinated / clinical trial	698	238439
7	S	Vaccinated / clinical trial	580	248678
8	S	Vaccinated / clinical trial	NE	194304
9	S	Vaccinated / clinical trial	NE	373573
10	S	Vaccinated / clinical trial	NE	NE
11	S	Vaccinated / clinical trial	1749	8230
12	S	Vaccinated / clinical trial	50	7790
13	S	Vaccinated / clinical trial	1423	283713
14	S	Vaccinated / clinical trial	311	142912
15	S	Vaccinated / clinical trial	648	172125
16	S	Vaccinated / clinical trial	7887	801525
17	S	Vaccinated / clinical trial	3969	470916
18	S	Vaccinated / clinical trial	264	97833
19	S	Vaccinated / clinical trial	83	101623
20	S	Vaccinated / clinical trial	328	173382
21	S	Vaccinated / clinical trial	1503	328500
22	S	Vaccinated / clinical trial	459	178218
23	S	Vaccinated / clinical trial	820	231477
24	S	Vaccinated / clinical trial	673	165356
25	S	Vaccinated / clinical trial	1239	342445
26	S	Vaccinated / clinical trial	1099	238261
27	S	Vaccinated / clinical trial	1072	187211
28	S	Vaccinated / clinical trial	1514	352741
29	S	Vaccinated / clinical trial	NE	212235
30	S	Vaccinated / clinical trial	939	181645
31	S	Vaccinated / clinical trial	753	155810
32	S	Vaccinated / clinical trial	2087	395255
33	S	Vaccinated / clinical trial	3661	372797
34	S	Vaccinated / clinical trial	2374	379060
35	S	Vaccinated / clinical trial	716	144258
36	S	Vaccinated / clinical trial	844	265125
37	S	Vaccinated / clinical trial	NE	361963

38	S	Vaccinated / clinical trial	NE	231795
39	S	Vaccinated / clinical trial	1452	390367
40	S	Vaccinated / clinical trial	NE	205145
41	S	Vaccinated / clinical trial	3529	456917
42	S	Vaccinated / clinical trial	1649	375620
43	S	Vaccinated / clinical trial	1330	223603
44	S	Vaccinated / clinical trial	1630	334526
45	S	Vaccinated / clinical trial	865	195570
46	S	Vaccinated / clinical trial	224	68886
47	S	Vaccinated / clinical trial	958	246579
48	S	Vaccinated / clinical trial	1232	372575
49	S	Vaccinated / clinical trial	1144	263551
50	S	Vaccinated / clinical trial	3241	525476
51	S	Vaccinated / clinical trial	510	166176
52	S	Vaccinated / clinical trial	3425	969092
53	S	Vaccinated / clinical trial	50	216
54	S	Vaccinated / clinical trial	828	166560
55	S	Vaccinated / clinical trial	50	55
56	S	Vaccinated / clinical trial	348	94231
57	S	Vaccinated / clinical trial	50	23
58	S	Vaccinated / clinical trial	949	322828
59	S	Vaccinated / clinical trial	50	23
60	S	Vaccinated / clinical trial	114	74214
61	S	Vaccinated / clinical trial	50	23
62	S	Vaccinated / clinical trial	1169	174083
63	S	Vaccinated / clinical trial	50	23
64	S	Vaccinated / clinical trial	636	121046
65	S	Vaccinated / clinical trial	50	23
66	S	Vaccinated / clinical trial	525	137000
67	S	Vaccinated / clinical trial	50	95
68	S	Vaccinated / clinical trial	386	85452
69	S	Vaccinated / clinical trial	50	35
70	S	Vaccinated / clinical trial	1086	374381
71	S	Vaccinated / clinical trial	50	23
72	S	Vaccinated / clinical trial	482	135979

Ab[C] = antibody concentration; AU = arbitrary units; MNT = microneutralization;
MSD ECL = multiplex electrochemiluminescence; NE = not estimable; S = spike.

(A) Spike antigen (continued)

Sample #	MSD Antigen	Status	MNT Ab[C] (AU/ml)	MSD ECL Ab[C] (AU/ml)
73	S	Vaccinated / clinical trial	50	89
74	S	Vaccinated / clinical trial	2723	490350
75	S	Vaccinated / clinical trial	50	23
76	S	Vaccinated / clinical trial	598	150507
77	S	Vaccinated / clinical trial	50	67
78	S	Vaccinated / clinical trial	2653	338048
79	S	Vaccinated / clinical trial	50	86
80	S	Vaccinated / clinical trial	4349	459170
81	S	Vaccinated / clinical trial	50	23
82	S	Vaccinated / clinical trial	2581	315277
83	S	Vaccinated / clinical trial	50	51
84	S	Vaccinated / clinical trial	392	211921
85	S	Vaccinated / clinical trial	50	269
86	S	Vaccinated / clinical trial	5752	1016706
87	S	Vaccinated / clinical trial	50	99
88	S	Vaccinated / clinical trial	NE	314025
89	S	Vaccinated / clinical trial	50	92
90	S	Vaccinated / clinical trial	2672	730687
91	S	Vaccinated / clinical trial	50	23
92	S	Vaccinated / clinical trial	610	304524
93	S	Vaccinated / clinical trial	50	35
94	S	Vaccinated / clinical trial	5382	1041230
95	S	Vaccinated / clinical trial	50	23
96	S	Vaccinated / clinical trial	554	204872
97	S	Vaccinated / clinical trial	50	23
98	S	Vaccinated / clinical trial	2028	585840
99	S	Vaccinated / clinical trial	50	23
100	S	Vaccinated / clinical trial	9676	1400000
101	S	Vaccinated / clinical trial	50	23
102	S	Vaccinated / clinical trial	448	302464
103	S	Vaccinated / clinical trial	50	232
104	S	Vaccinated / clinical trial	50	206195
105	S	Vaccinated / clinical trial	619	85
106	S	Vaccinated / clinical trial	214	75683
107	S	Vaccinated / clinical trial	6115	92833
108	S	Vaccinated / clinical trial	50	427939
109	S	Vaccinated / clinical trial	2094	151

110	S	Vaccinated / clinical trial	50	460932
111	S	Vaccinated / clinical trial	3258	62
112	S	Vaccinated / clinical trial	1598	592741
113	S	Convalescent	NE	5606
114	S	Convalescent	NE	18438
115	S	Convalescent	NE	2045
116	S	Convalescent	NE	6598
117	S	Convalescent	NE	6021
118	S	Convalescent	NE	15886
119	S	Convalescent	NE	43
120	S	Convalescent	NE	5717
121	S	Convalescent	NE	4469
122	S	Convalescent	53	17437
123	S	Convalescent	55	6766
124	S	Convalescent	58	6597
125	S	Convalescent	59	16562
126	S	Convalescent	81	35706
127	S	Convalescent	82	13307
128	S	Convalescent	97	14483
129	S	Convalescent	119	210731
130	S	Convalescent	120	10041
131	S	Convalescent	162	26339
132	S	Convalescent	180	201035
133	S	Convalescent	207	80663
134	S	Convalescent	248	49870
135	S	Convalescent	261	31841
136	S	Convalescent	287	34288
137	S	Convalescent	307	169048
138	S	Convalescent	328	174337
139	S	Convalescent	361	78879
140	S	Convalescent	446	207450
141	S	Convalescent	494	NE
142	S	Convalescent	590	490090
143	S	Convalescent	659	NE
144	S	Convalescent	711	280828

Ab[C] = antibody concentration; AU = arbitrary units; MNT = microneutralization;
MSD ECL = multiplex electrochemiluminescence; NE = not estimable; S = spike.

(A) Spike antigen (continued)

Sample #	MSD Antigen	Status	MNT Ab[C] (AU/ml)	MSD ECL Ab[C] (AU/ml)
113	S	Convalescent	NE	5606
114	S	Convalescent	NE	18438
115	S	Convalescent	NE	2045
116	S	Convalescent	NE	6598
117	S	Convalescent	NE	6021
118	S	Convalescent	NE	15886
119	S	Convalescent	NE	43
120	S	Convalescent	NE	5717
121	S	Convalescent	NE	4469
122	S	Convalescent	53	17437
123	S	Convalescent	55	6766
124	S	Convalescent	58	6597
125	S	Convalescent	59	16562
126	S	Convalescent	81	35706
127	S	Convalescent	82	13307
128	S	Convalescent	97	14483
129	S	Convalescent	119	210731
130	S	Convalescent	120	10041
131	S	Convalescent	162	26339
132	S	Convalescent	180	201035
133	S	Convalescent	207	80663
134	S	Convalescent	248	49870
135	S	Convalescent	261	31841
136	S	Convalescent	287	34288
137	S	Convalescent	307	169048
138	S	Convalescent	328	174337
139	S	Convalescent	361	78879
140	S	Convalescent	446	207450
141	S	Convalescent	494	NE
142	S	Convalescent	590	490090
143	S	Convalescent	659	NE

Ab[C] = antibody concentration; AU = arbitrary units; MNT = microneutralization;
MSD ECL = multiplex electrochemiluminescence; NE = not estimable; S = spike.

S5 Table. Correlation between the results of the MNT and MSD ECL assays

(B) Nucleocapsid antigen

Sample #	MSD Antigen	Status	MNT Ab[C] (AU/ml)	MSD ECL Ab[C] (AU/ml)
1	N	Vaccinated / clinical trial	NE	27
2	N	Vaccinated / clinical trial	50	130
3	N	Vaccinated / clinical trial	2310	217
4	N	Vaccinated / clinical trial	NE	5804
5	N	Vaccinated / clinical trial	119	167
6	N	Vaccinated / clinical trial	698	47
7	N	Vaccinated / clinical trial	580	785
8	N	Vaccinated / clinical trial	NE	1056
9	N	Vaccinated / clinical trial	NE	44
10	N	Vaccinated / clinical trial	NE	156283
11	N	Vaccinated / clinical trial	1749	NE
12	N	Vaccinated / clinical trial	50	791
13	N	Vaccinated / clinical trial	1423	NE
14	N	Vaccinated / clinical trial	311	134
15	N	Vaccinated / clinical trial	648	807
16	N	Vaccinated / clinical trial	7887	36725
17	N	Vaccinated / clinical trial	3969	13369
18	N	Vaccinated / clinical trial	264	578
19	N	Vaccinated / clinical trial	83	72
20	N	Vaccinated / clinical trial	328	600
21	N	Vaccinated / clinical trial	1503	61
22	N	Vaccinated / clinical trial	459	27
23	N	Vaccinated / clinical trial	820	96
24	N	Vaccinated / clinical trial	673	248
25	N	Vaccinated / clinical trial	1239	117
26	N	Vaccinated / clinical trial	1099	27
27	N	Vaccinated / clinical trial	1072	63
28	N	Vaccinated / clinical trial	1514	30
29	N	Vaccinated / clinical trial	NE	27
30	N	Vaccinated / clinical trial	939	NE
31	N	Vaccinated / clinical trial	753	252
32	N	Vaccinated / clinical trial	2087	180
33	N	Vaccinated / clinical trial	3661	123
34	N	Vaccinated / clinical trial	2374	7713
35	N	Vaccinated / clinical trial	716	27
36	N	Vaccinated / clinical trial	844	2587
37	N	Vaccinated / clinical trial	NE	460
38	N	Vaccinated / clinical trial	NE	48
39	N	Vaccinated / clinical trial	1452	179
40	N	Vaccinated / clinical trial	NE	7193
41	N	Vaccinated / clinical trial	3529	NE
42	N	Vaccinated / clinical trial	1649	NE
43	N	Vaccinated / clinical trial	1330	314

44	N	Vaccinated / clinical trial	1630	4776
45	N	Vaccinated / clinical trial	865	NE
46	N	Vaccinated / clinical trial	224	341
47	N	Vaccinated / clinical trial	958	352
48	N	Vaccinated / clinical trial	1232	3267
49	N	Vaccinated / clinical trial	1144	NE
50	N	Vaccinated / clinical trial	3241	NE
51	N	Vaccinated / clinical trial	510	100
52	N	Vaccinated / clinical trial	3425	65
53	N	Vaccinated / clinical trial	50	75
54	N	Vaccinated / clinical trial	828	86
55	N	Vaccinated / clinical trial	50	143
56	N	Vaccinated / clinical trial	348	147
57	N	Vaccinated / clinical trial	50	623
58	N	Vaccinated / clinical trial	949	713
59	N	Vaccinated / clinical trial	50	27
60	N	Vaccinated / clinical trial	114	27
61	N	Vaccinated / clinical trial	50	34
62	N	Vaccinated / clinical trial	1169	38
63	N	Vaccinated / clinical trial	50	27
64	N	Vaccinated / clinical trial	636	27
65	N	Vaccinated / clinical trial	50	56
66	N	Vaccinated / clinical trial	525	46
67	N	Vaccinated / clinical trial	50	133
68	N	Vaccinated / clinical trial	386	136
69	N	Vaccinated / clinical trial	50	126
70	N	Vaccinated / clinical trial	1086	147
71	N	Vaccinated / clinical trial	50	108
72	N	Vaccinated / clinical trial	482	151

Ab[C] = antibody concentration; AU = arbitrary units; MNT = microneutralization;
MSD ECL = multiplex electrochemiluminescence; N = nucleocapsid; NE = not estimable.

(B) Nucleocapsid antigen (continued)

Sample #	MSD Antigen	Status	MNT Ab[C] (AU/ml)	MSD ECL Ab[C] (AU/ml)
73	N	Vaccinated / clinical trial	50	990
74	N	Vaccinated / clinical trial	2723	796
75	N	Vaccinated / clinical trial	50	884
76	N	Vaccinated / clinical trial	598	861
77	N	Vaccinated / clinical trial	50	361
78	N	Vaccinated / clinical trial	2653	606
79	N	Vaccinated / clinical trial	50	50
80	N	Vaccinated / clinical trial	4349	47
81	N	Vaccinated / clinical trial	50	27
82	N	Vaccinated / clinical trial	2581	37
83	N	Vaccinated / clinical trial	50	52
84	N	Vaccinated / clinical trial	392	65
85	N	Vaccinated / clinical trial	50	479
86	N	Vaccinated / clinical trial	5752	578
87	N	Vaccinated / clinical trial	50	147
88	N	Vaccinated / clinical trial	NE	161
89	N	Vaccinated / clinical trial	50	132
90	N	Vaccinated / clinical trial	2672	158
91	N	Vaccinated / clinical trial	50	27
92	N	Vaccinated / clinical trial	610	NE
93	N	Vaccinated / clinical trial	50	79
94	N	Vaccinated / clinical trial	5382	116
95	N	Vaccinated / clinical trial	50	92
96	N	Vaccinated / clinical trial	554	138
97	N	Vaccinated / clinical trial	50	1072
98	N	Vaccinated / clinical trial	2028	1136
99	N	Vaccinated / clinical trial	50	29
100	N	Vaccinated / clinical trial	9676	7379
101	N	Vaccinated / clinical trial	50	174
102	N	Vaccinated / clinical trial	448	193
103	N	Vaccinated / clinical trial	50	338
104	N	Vaccinated / clinical trial	50	297
105	N	Vaccinated / clinical trial	619	86
106	N	Vaccinated / clinical trial	214	88
107	N	Vaccinated / clinical trial	6115	70167
108	N	Vaccinated / clinical trial	50	27226
109	N	Vaccinated / clinical trial	2094	465
110	N	Vaccinated / clinical trial	50	475
111	N	Vaccinated / clinical trial	3258	NE
112	N	Vaccinated / clinical trial	1598	29
113	N	Convalescent	NE	13343
114	N	Convalescent	NE	21232
115	N	Convalescent	NE	20580

116	N	Convalescent	NE	72559
117	N	Convalescent	NE	51586
118	N	Convalescent	NE	47526
119	N	Convalescent	NE	1478
120	N	Convalescent	NE	9575
121	N	Convalescent	NE	8953
122	N	Convalescent	53	32208
123	N	Convalescent	55	32254
124	N	Convalescent	58	13182
125	N	Convalescent	59	73824
126	N	Convalescent	81	84876
127	N	Convalescent	82	42069
128	N	Convalescent	97	19014
129	N	Convalescent	119	395458
130	N	Convalescent	120	66576
131	N	Convalescent	162	126650
132	N	Convalescent	180	434408
133	N	Convalescent	207	1600000
134	N	Convalescent	248	53305
135	N	Convalescent	261	31335
136	N	Convalescent	287	13234
137	N	Convalescent	307	290548
138	N	Convalescent	328	1600000
139	N	Convalescent	361	126360
140	N	Convalescent	446	85929
141	N	Convalescent	494	NE
142	N	Convalescent	590	295070
143	N	Convalescent	659	NE
144	N	Convalescent	711	NE

Ab[C] = antibody concentration; AU = arbitrary units; MNT = microneutralization;
MSD ECL = multiplex electrochemiluminescence; N = nucleocapsid; NE = not estimable.

S5 Table. Correlation between the results of the MNT and MSD ECL assays

(C) Receptor-binding domain antigen

Sample #	MSD Antigen	Status	MNT Ab[C] (AU/ml)	MSD ECL Ab[C] (AU/ml)
1	RBD	Vaccinated / clinical trial	NE	NE
2	RBD	Vaccinated / clinical trial	50	9402
3	RBD	Vaccinated / clinical trial	2310	291219
4	RBD	Vaccinated / clinical trial	NE	107194
5	RBD	Vaccinated / clinical trial	119	39254
6	RBD	Vaccinated / clinical trial	698	99479
7	RBD	Vaccinated / clinical trial	580	100989
8	RBD	Vaccinated / clinical trial	NE	69499
9	RBD	Vaccinated / clinical trial	NE	190600
10	RBD	Vaccinated / clinical trial	NE	506427
11	RBD	Vaccinated / clinical trial	1749	1743
12	RBD	Vaccinated / clinical trial	50	469
13	RBD	Vaccinated / clinical trial	1423	119339
14	RBD	Vaccinated / clinical trial	311	64377
15	RBD	Vaccinated / clinical trial	648	65901
16	RBD	Vaccinated / clinical trial	7887	292988
17	RBD	Vaccinated / clinical trial	3969	193789
18	RBD	Vaccinated / clinical trial	264	35283
19	RBD	Vaccinated / clinical trial	83	32812
20	RBD	Vaccinated / clinical trial	328	70272
21	RBD	Vaccinated / clinical trial	1503	151600
22	RBD	Vaccinated / clinical trial	459	76221
23	RBD	Vaccinated / clinical trial	820	85261
24	RBD	Vaccinated / clinical trial	673	62557
25	RBD	Vaccinated / clinical trial	1239	143183
26	RBD	Vaccinated / clinical trial	1099	117554
27	RBD	Vaccinated / clinical trial	1072	81598
28	RBD	Vaccinated / clinical trial	1514	138207
29	RBD	Vaccinated / clinical trial	NE	97052
30	RBD	Vaccinated / clinical trial	939	77974
31	RBD	Vaccinated / clinical trial	753	77174
32	RBD	Vaccinated / clinical trial	2087	139647
33	RBD	Vaccinated / clinical trial	3661	188458
34	RBD	Vaccinated / clinical trial	2374	187199
35	RBD	Vaccinated / clinical trial	716	74168
36	RBD	Vaccinated / clinical trial	844	116574
37	RBD	Vaccinated / clinical trial	NE	128412
38	RBD	Vaccinated / clinical trial	NE	103793
39	RBD	Vaccinated / clinical trial	1452	193974
40	RBD	Vaccinated / clinical trial	NE	86471
41	RBD	Vaccinated / clinical trial	3529	247877
42	RBD	Vaccinated / clinical trial	1649	160158
43	RBD	Vaccinated / clinical trial	1330	102269

44	RBD	Vaccinated / clinical trial	1630	136643
45	RBD	Vaccinated / clinical trial	865	78869
46	RBD	Vaccinated / clinical trial	224	25637
47	RBD	Vaccinated / clinical trial	958	103338
48	RBD	Vaccinated / clinical trial	1232	174450
49	RBD	Vaccinated / clinical trial	1144	130807
50	RBD	Vaccinated / clinical trial	3241	236282
51	RBD	Vaccinated / clinical trial	510	77738
52	RBD	Vaccinated / clinical trial	3425	485300
53	RBD	Vaccinated / clinical trial	50	192
54	RBD	Vaccinated / clinical trial	828	68532
55	RBD	Vaccinated / clinical trial	50	22
56	RBD	Vaccinated / clinical trial	348	31207
57	RBD	Vaccinated / clinical trial	50	19
58	RBD	Vaccinated / clinical trial	949	152511
59	RBD	Vaccinated / clinical trial	50	19
60	RBD	Vaccinated / clinical trial	114	27820
61	RBD	Vaccinated / clinical trial	50	19
62	RBD	Vaccinated / clinical trial	1169	89754
63	RBD	Vaccinated / clinical trial	50	19
64	RBD	Vaccinated / clinical trial	636	51082
65	RBD	Vaccinated / clinical trial	50	19
66	RBD	Vaccinated / clinical trial	525	54883
67	RBD	Vaccinated / clinical trial	50	19
68	RBD	Vaccinated / clinical trial	386	35431
69	RBD	Vaccinated / clinical trial	50	27
70	RBD	Vaccinated / clinical trial	1086	160952
71	RBD	Vaccinated / clinical trial	50	19
72	RBD	Vaccinated / clinical trial	482	51314

Ab[C] = antibody concentration; AU = arbitrary units; MNT = microneutralization; MSD ECL = multiplex electrochemiluminescence; NE = not estimable; RBD = receptor-binding domain.

(C) Receptor-binding domain antigen (continued)

Sample #	MSD Antigen	Status	MNT Ab[C] (AU/ml)	MSD ECL Ab[C] (AU/ml)
73	RBD	Vaccinated / clinical trial	50	19
74	RBD	Vaccinated / clinical trial	2723	211743
75	RBD	Vaccinated / clinical trial	50	19
76	RBD	Vaccinated / clinical trial	598	65699
77	RBD	Vaccinated / clinical trial	50	105
78	RBD	Vaccinated / clinical trial	2653	137807
79	RBD	Vaccinated / clinical trial	50	19
80	RBD	Vaccinated / clinical trial	4349	197715
81	RBD	Vaccinated / clinical trial	50	19
82	RBD	Vaccinated / clinical trial	2581	148673
83	RBD	Vaccinated / clinical trial	50	20
84	RBD	Vaccinated / clinical trial	392	97929
85	RBD	Vaccinated / clinical trial	50	852
86	RBD	Vaccinated / clinical trial	5752	485268
87	RBD	Vaccinated / clinical trial	50	164
88	RBD	Vaccinated / clinical trial	NE	125952
89	RBD	Vaccinated / clinical trial	50	101
90	RBD	Vaccinated / clinical trial	2672	369297
91	RBD	Vaccinated / clinical trial	50	19
92	RBD	Vaccinated / clinical trial	610	152439
93	RBD	Vaccinated / clinical trial	50	19
94	RBD	Vaccinated / clinical trial	5382	511086
95	RBD	Vaccinated / clinical trial	50	19
96	RBD	Vaccinated / clinical trial	554	88272
97	RBD	Vaccinated / clinical trial	50	19
98	RBD	Vaccinated / clinical trial	2028	284702
99	RBD	Vaccinated / clinical trial	50	19
100	RBD	Vaccinated / clinical trial	9676	600000
101	RBD	Vaccinated / clinical trial	50	38
102	RBD	Vaccinated / clinical trial	448	116098
103	RBD	Vaccinated / clinical trial	50	396
104	RBD	Vaccinated / clinical trial	50	96689
105	RBD	Vaccinated / clinical trial	619	19
106	RBD	Vaccinated / clinical trial	214	28909
107	RBD	Vaccinated / clinical trial	6115	28477
108	RBD	Vaccinated / clinical trial	50	147463
109	RBD	Vaccinated / clinical trial	2094	19
110	RBD	Vaccinated / clinical trial	50	180303
111	RBD	Vaccinated / clinical trial	3258	19
112	RBD	Vaccinated / clinical trial	1598	293563
113	RBD	Convalescent	NE	1052
114	RBD	Convalescent	NE	8047
115	RBD	Convalescent	NE	538

116	RBD	Convalescent	NE	2111
117	RBD	Convalescent	NE	2431
118	RBD	Convalescent	NE	3152
119	RBD	Convalescent	NE	19
120	RBD	Convalescent	NE	2216
121	RBD	Convalescent	NE	1817
122	RBD	Convalescent	53	4157
123	RBD	Convalescent	55	1630
124	RBD	Convalescent	58	1913
125	RBD	Convalescent	59	5934
126	RBD	Convalescent	81	14076
127	RBD	Convalescent	82	4587
128	RBD	Convalescent	97	3612
129	RBD	Convalescent	119	54663
130	RBD	Convalescent	120	2132
131	RBD	Convalescent	162	8321
132	RBD	Convalescent	180	78790
133	RBD	Convalescent	207	24316
134	RBD	Convalescent	248	9165
135	RBD	Convalescent	261	11465
136	RBD	Convalescent	287	12260
137	RBD	Convalescent	307	57211
138	RBD	Convalescent	328	67484
139	RBD	Convalescent	361	19306
140	RBD	Convalescent	446	77353
141	RBD	Convalescent	494	NE
142	RBD	Convalescent	590	195697
143	RBD	Convalescent	659	NE
144	RBD	Convalescent	711	71145

Ab[C] = antibody concentration; AU = arbitrary units; MNT = microneutralization; MSD ECL = multiplex electrochemiluminescence; NE = not estimable; RBD = receptor-binding domain.
