# Supplementary material for: Robust validation and performance comparison of immunogenicity assays assessing IgG and neutralizing antibodies to SARS-CoV-2
Source: PLoS One. 2022 Feb 7;17(2):e0262922. doi: 10.1371/journal.pone.0262922 (PMC8820625; doi:10.1371/journal.pone.0262922)
Supplement: S6 Table — MNT = microneutralization; PPD = Pharmaceutical Product Development; WHO = World Health Organization. (PDF) [file pone.0262922.s007.pdf]

S6 Table. MNT reference standard calibration to WHO international reference panel.

Run #	Concentration (IU/ml)	Value	Response	Category	Run #	Concentration (IU/ml)	Value	Response	Category
1	25.5	4.50	8.14	PPD	4	25.5	9.50	12.91	PPD
1	12.75	20.00	22.45	PPD	4	12.75	30.50	32.68	PPD
1	6.375	55.00	56.84	PPD	4	6.375	76.50	77.85	PPD
1	3.1875	122.00	120.20	PPD	4	3.1875	167.00	163.49	PPD
1	1.59375	200.50	194.96	PPD	4	1.59375	290.50	280.44	PPD
1	0.796875	239.50	248.04	PPD	4	0.796875	362.50	384.74	PPD
1	0.3984375	273.50	273.68	PPD	4	0.3984375	440.50	447.88	PPD
1	0.19921875	275.50	283.77	PPD	4	0.19921875	522.00	477.59	PPD
1	0.099609375	309.00	287.42	PPD	4	0.099609375	505.00	489.91	PPD
1	0.049804688	279.00	288.69	PPD	4	0.049804688	454.50	494.75	PPD
1	20	40.00	33.67	WHO	4	20	33.50	29.77	WHO
1	10	33.00	46.96	WHO	4	10	51.00	62.71	WHO
1	5	92.00	82.70	WHO	4	5	111.50	121.98	WHO
1	2.5	156.50	153.41	WHO	4	2.5	226.50	208.47	WHO
1	1.25	220.50	235.18	WHO	4	1.25	308.00	302.71	WHO
1	0.625	305.00	287.07	WHO	4	0.625	364.50	377.66	WHO
1	0.3125	296.00	308.70	WHO	4	0.3125	417.50	423.72	WHO
1	0.15625	346.00	316.11	WHO	4	0.15625	450.00	447.69	WHO
1	0.078125	292.00	318.48	WHO	4	0.078125	452.50	459.09	WHO
1	0.0390625	320.50	319.21	WHO	4	0.0390625	477.50	464.28	WHO
2	25.5	6.00	20.53	PPD	5	25.5	14.00	33.37	PPD
2	12.75	27.50	27.10	PPD	5	12.75	42.50	58.69	PPD
2	6.375	75.50	49.88	PPD	5	6.375	105.00	99.54	PPD
2	3.1875	98.00	111.14	PPD	5	3.1875	147.00	159.63	PPD
2	1.59375	199.50	203.71	PPD	5	1.59375	247.00	237.00	PPD
2	0.796875	277.00	267.90	PPD	5	0.796875	351.50	321.18	PPD
2	0.3984375	308.50	292.39	PPD	5	0.3984375	420.00	397.56	PPD
2	0.19921875	295.00	299.52	PPD	5	0.19921875	386.00	456.23	PPD
2	0.099609375	283.50	301.42	PPD	5	0.099609375	462.50	495.80	PPD
2	0.049804688	305.00	301.91	PPD	5	0.049804688	579.00	520.20	PPD
2	20	7.50	16.65	WHO	5	20	12.50	20.70	WHO
2	10	37.00	38.33	WHO	5	10	38.50	49.10	WHO
2	5	80.00	81.92	WHO	5	5	108.00	104.61	WHO
2	2.5	157.00	152.98	WHO	5	2.5	203.00	185.90	WHO
2	1.25	235.50	236.97	WHO	5	1.25	246.00	265.76	WHO
2	0.625	313.50	305.49	WHO	5	0.625	328.00	318.70	WHO
2	0.3125	347.50	346.41	WHO	5	0.3125	336.00	345.31	WHO
2	0.15625	327.00	366.46	WHO	5	0.15625	366.00	356.83	WHO
2	0.078125	389.50	375.33	WHO	5	0.078125	335.50	361.49	WHO
2	0.0390625	396.00	379.09	WHO	5	0.0390625	387.50	363.33	WHO
3	25.5	15.00	33.62	PPD	6	25.5	9.50	25.35	PPD
3	12.75	60.50	46.83	PPD	6	12.75	25.50	47.07	PPD
3	6.375	93.50	84.48	PPD	6	6.375	83.00	83.56	PPD

3	3.1875	173.50	170.52	PPD
3	1.59375	277.00	296.23	PPD
3	0.796875	407.00	396.93	PPD
3	0.3984375	473.00	445.52	PPD
3	0.19921875	456.50	463.29	PPD
3	0.099609375	431.00	469.12	PPD
3	0.049804688	490.50	470.96	PPD
3	20	39.50	38.25	WHO
3	10	55.50	58.54	WHO
3	5	124.00	123.96	WHO
3	2.5	268.50	258.21	WHO
3	1.25	355.00	385.08	WHO
3	0.625	481.50	441.98	WHO
3	0.3125	475.00	459.03	WHO
3	0.15625	439.50	463.48	WHO
3	0.078125	449.50	464.60	WHO
3	0.0390625	470.00	464.87	WHO

6	3.1875	148.50	138.16	PPD
6	1.59375	218.50	207.19	PPD
6	0.796875	298.00	278.00	PPD
6	0.3984375	304.50	336.66	PPD
6	0.19921875	352.50	377.24	PPD
6	0.099609375	422.00	401.95	PPD
6	0.049804688	426.00	415.83	PPD
6	20	30.50	43.69	WHO
6	10	62.50	76.09	WHO
6	5	130.50	125.50	WHO
6	2.5	214.00	191.56	WHO
6	1.25	275.50	265.86	WHO
6	0.625	311.50	334.81	WHO
6	0.3125	354.00	388.29	WHO
6	0.15625	453.50	424.27	WHO
6	0.078125	492.50	446.22	WHO
6	0.0390625	420.00	458.83	WHO

IU = international units; MNT = microneutralization; PPD = Pharmaceutical Product Development;
WHO = World Health Organization.

Run #	Concentration (IU/ml)	Value	Response	Category
7	25.5	13.50	27.85	PPD
7	12.75	31.00	34.67	PPD
7	6.375	96.50	65.34	PPD
7	3.1875	147.50	166.61	PPD
7	1.59375	324.00	318.65	PPD
7	0.796875	411.50	400.17	PPD
7	0.3984375	414.00	422.41	PPD
7	0.19921875	449.50	427.21	PPD
7	0.099609375	419.50	428.20	PPD
7	0.049804688	412.50	428.39	PPD
7	20	29.50	28.10	WHO
7	10	53.50	56.63	WHO
7	5	119.00	112.15	WHO
7	2.5	186.00	198.33	WHO
7	1.25	302.00	294.26	WHO
7	0.625	378.00	368.48	WHO
7	0.3125	403.50	411.34	WHO
7	0.15625	415.00	432.03	WHO
7	0.078125	436.50	441.16	WHO
7	0.0390625	464.50	445.02	WHO
8	25.5	15.50	27.16	PPD
8	12.75	42.50	39.27	PPD
8	6.375	94.50	78.17	PPD
8	3.1875	164.00	170.07	PPD
8	1.59375	269.00	287.61	PPD
8	0.796875	400.50	359.76	PPD
8	0.3984375	382.00	386.36	PPD
8	0.19921875	355.00	394.23	PPD
8	0.099609375	392.50	396.40	PPD
8	0.049804688	420.50	396.99	PPD
8	20	21.00	24.82	WHO
8	10	61.50	54.39	WHO
8	5	117.00	119.96	WHO
8	2.5	223.50	227.88	WHO
8	1.25	352.00	340.90	WHO
8	0.625	402.50	414.92	WHO
8	0.3125	450.50	449.76	WHO
8	0.15625	479.00	463.59	WHO
8	0.078125	454.50	468.71	WHO
8	0.0390625	474.00	470.56	WHO
9	25.5	14.50	9.54	PPD
9	12.75	17.00	23.59	PPD
9	6.375	56.00	55.27	PPD

Run #	Concentration (IU/ml)	Value	Response	Category
10	25.5	5.50	21.26	PPD
10	12.75	27.00	34.72	PPD
10	6.375	42.50	55.75	PPD
10	3.1875	90.50	87.22	PPD
10	1.59375	158.00	131.43	PPD
10	0.796875		188.29	PPD
10	0.3984375		253.69	PPD
10	0.19921875	291.50	319.89	PPD
10	0.099609375	398.50	378.78	PPD
10	0.049804688	423.50	425.44	PPD
10	20	23.50	22.54	WHO
10	10	44.00	44.79	WHO
10	5	93.00	92.35	WHO
10	2.5	172.50	173.53	WHO
10	1.25	262.00	270.36	WHO
10	0.625	380.50	346.65	WHO
10	0.3125	343.50	389.43	WHO
10	0.15625	409.00	408.98	WHO
10	0.078125	458.50	417.08	WHO
10	0.0390625	399.50	420.29	WHO
11	25.5	20.50	50.84	PPD
11	12.75	62.50	52.39	PPD
11	6.375	77.00	61.78	PPD
11	3.1875	124.00	110.85	PPD
11	1.59375	236.00	252.53	PPD
11	0.796875	383.50	374.91	PPD
11	0.3984375	453.50	409.87	PPD
11	0.19921875	430.00	416.20	PPD
11	0.099609375	421.50	417.24	PPD
11	0.049804688	355.50	417.40	PPD
11	20	26.50	24.88	WHO
11	10	49.00	49.25	WHO
11	5	101.50	100.61	WHO
11	2.5	179.00	188.76	WHO
11	1.25	311.00	297.50	WHO
11	0.625	382.50	387.96	WHO
11	0.3125	448.50	441.66	WHO
11	0.15625	441.50	467.40	WHO
11	0.078125	485.50	478.47	WHO
11	0.0390625	494.50	483.01	WHO
12	25.5	10.00	14.78	PPD
12	12.75	36.50	30.28	PPD
12	6.375	63.00	66.03	PPD

9	3.1875	116.50	115.76	PPD
9	1.59375	202.00	201.63	PPD
9	0.796875	278.50	283.45	PPD
9	0.3984375	360.00	336.60	PPD
9	0.19921875	320.00	363.09	PPD
9	0.099609375	397.50	374.56	PPD
9	0.049804688	380.50	379.22	PPD
9	20	10.00	19.82	WHO
9	10	16.50	33.57	WHO
9	5	57.00	55.52	WHO
9	2.5	112.50	88.50	WHO
9	1.25	146.50	133.74	WHO
9	0.625	170.00	188.70	WHO
9	0.3125	229.50	246.39	WHO
9	0.15625	296.50	298.35	WHO
9	0.078125	382.00	339.09	WHO
9	0.0390625	342.00	367.70	WHO

12	3.1875	142.50	138.41	PPD
12	1.59375	248.50	252.73	PPD
12	0.796875	382.50	377.66	PPD
12	0.3984375	456.50	469.49	PPD
12	0.19921875	537.50	519.21	PPD
12	0.099609375	541.50	541.76	PPD
12	0.049804688	543.00	551.17	PPD
12	20	16.50	27.02	WHO
12	10	46.50	56.45	WHO
12	5	122.00	110.80	WHO
12	2.5	183.00	195.62	WHO
12	1.25	322.50	298.55	WHO
12	0.625	382.00	391.27	WHO
12	0.3125	436.00	454.88	WHO
12	0.15625	500.50	490.81	WHO
12	0.078125	507.00	508.92	WHO
12	0.0390625	524.00	517.51	WHO

IU = international units; MNT = microneutralization; PPD = Pharmaceutical Product Development;
WHO = World Health Organization.

Run #	Concentration (IU/ml)	Value	Response	Category
13	25.5	29.00	33.75	PPD
13	12.75	60.50	54.96	PPD
13	6.375	103.50	102.55	PPD
13	3.1875	187.50	193.85	PPD
13	1.59375	340.00	326.06	PPD
13	0.796875	440.50	456.27	PPD
13	0.3984375	533.50	543.91	PPD
13	0.19921875	634.50	588.88	PPD
13	0.099609375	586.00	608.77	PPD
13	0.049804688	611.00	616.99	PPD
13	20	25.50	37.13	WHO
13	10	65.00	63.45	WHO
13	5	146.00	122.00	WHO
13	2.5	213.50	230.36	WHO
13	1.25	382.00	376.28	WHO
13	0.625	483.50	506.95	WHO
13	0.3125	630.50	587.58	WHO
13	0.15625	625.50	626.49	WHO
13	0.078125	607.00	643.05	WHO
13	0.0390625	664.50	649.72	WHO
14	25.5	17.50	26.76	PPD
14	12.75	58.50	52.65	PPD
14	6.375	121.50	110.14	PPD
14	3.1875	209.50	216.01	PPD
14	1.59375	344.00	357.33	PPD
14	0.796875	505.50	482.50	PPD
14	0.3984375	554.50	559.03	PPD
14	0.19921875	570.50	595.74	PPD
14	0.099609375	642.50	611.30	PPD
14	0.049804688	605.00	617.54	PPD
14	20	45.00	48.36	WHO
14	10	71.00	68.07	WHO
14	5	113.00	114.18	WHO
14	2.5	212.50	204.17	WHO
14	1.25	315.50	330.73	WHO
14	0.625	457.00	445.92	WHO
14	0.3125	520.00	515.97	WHO
14	0.15625	544.00	548.70	WHO
14	0.078125	549.00	562.10	WHO
14	0.0390625	578.50	567.29	WHO
15	25.5	8.50	37.49	PPD
15	12.75	46.50	46.17	PPD
15	6.375	114.00	76.65	PPD

Run #	Concentration (IU/ml)	Value	Response	Category
16	25.5	8.50	14.38	PPD
16	12.75	32.00	38.90	PPD
16	6.375	110.50	96.96	PPD
16	3.1875	202.50	203.74	PPD
16	1.59375	317.50	332.23	PPD
16	0.796875	439.00	426.75	PPD
16	0.3984375	503.00	474.11	PPD
16	0.19921875	441.00	493.34	PPD
16	0.099609375	500.00	500.46	PPD
16	0.049804688	525.50	503.01	PPD
16	20	39.00	29.52	WHO
16	10	57.00	73.87	WHO
16	5	158.50	164.87	WHO
16	2.5	317.50	301.19	WHO
16	1.25	431.00	432.56	WHO
16	0.625	515.50	515.10	WHO
16	0.3125	535.00	553.93	WHO
16	0.15625	547.50	569.70	WHO
16	0.078125	568.50	575.73	WHO
16	0.0390625	621.00	577.97	WHO
17	25.5	21.50	39.33	PPD
17	12.75	60.00	49.26	PPD
17	6.375	98.00	83.30	PPD
17	3.1875	167.00	176.93	PPD
17	1.59375	335.50	330.59	PPD
17	0.796875	432.00	450.10	PPD
17	0.3984375	543.50	499.99	PPD
17	0.19921875	509.50	515.30	PPD
17	0.099609375	500.50	519.53	PPD
17	0.049804688	517.50	520.67	PPD
17	20	36.00	43.14	WHO
17	10	70.50	60.38	WHO
17	5	101.50	107.36	WHO
17	2.5	214.50	207.65	WHO
17	1.25	337.50	342.25	WHO
17	0.625	435.50	443.03	WHO
17	0.3125	497.00	490.38	WHO
17	0.15625	543.00	507.78	WHO
17	0.078125	504.50	513.58	WHO
17	0.0390625	491.00	515.45	WHO
18	25.5	19.50	39.83	PPD
18	12.75	56.00	50.61	PPD
18	6.375	102.50	85.70	PPD

15	3.1875	170.00	165.31	PPD	18	3.1875	190.50	177.29	PPD
15	1.59375	290.00	326.05	PPD	18	1.59375	287.00	322.68	PPD
15	0.796875	504.50	465.13	PPD	18	0.796875	455.00	436.79	PPD
15	0.3984375	508.50	526.92	PPD	18	0.3984375	529.50	486.48	PPD
15	0.19921875	596.00	546.23	PPD	18	0.19921875	501.00	502.51	PPD
15	0.099609375	572.00	551.57	PPD	18	0.099609375	481.50	507.16	PPD
15	0.049804688	484.50	552.99	PPD	18	0.049804688	495.00	508.46	PPD
15	20	22.50	37.97	WHO	18	20	29.00	28.35	WHO
15	10	67.50	73.76	WHO	18	10	67.00	60.15	WHO
15	5	157.50	133.52	WHO	18	5	117.00	126.95	WHO
15	2.5	199.50	216.99	WHO	18	2.5	221.00	231.93	WHO
15	1.25	321.00	308.57	WHO	18	1.25	374.50	339.75	WHO
15	0.625	373.00	385.90	WHO	18	0.625	391.50	411.47	WHO
15	0.3125	457.50	438.02	WHO	18	0.3125	437.00	446.49	WHO
15	0.15625	444.00	468.03	WHO	18	0.15625	440.00	461.02	WHO
15	0.078125	480.50	483.76	WHO	18	0.078125	439.00	466.64	WHO
15	0.0390625	506.00	491.61	WHO	18	0.0390625	525.50	468.75	WHO

IU = international units; MNT = microneutralization; PPD = Pharmaceutical Product Development;
WHO = World Health Organization.

Run #	Concentration (IU/ml)	Value	Response	Category
19	25.5	9.50	14.36	PPD
19	12.75	30.50	27.72	PPD
19	6.375	69.00	60.68	PPD
19	3.1875	115.00	128.27	PPD
19	1.59375	241.00	226.09	PPD
19	0.796875	303.50	314.45	PPD
19	0.3984375	352.50	366.13	PPD
19	0.19921875	422.00	389.06	PPD
19	0.099609375	412.00	397.98	PPD
19	0.049804688	371.00	401.26	PPD
19	20	13.00	15.12	WHO
19	10	23.50	34.71	WHO
19	5	74.50	74.99	WHO
19	2.5	149.50	144.43	WHO
19	1.25	238.00	234.35	WHO
19	0.625	315.50	315.80	WHO
19	0.3125	360.00	368.93	WHO
19	0.15625	391.00	396.58	WHO
19	0.078125	419.00	409.29	WHO
19	0.0390625	416.00	414.80	WHO
20	25.5	16.50	18.80	PPD
20	12.75	18.50	28.99	PPD
20	6.375	75.00	55.89	PPD
20	3.1875	113.50	115.71	PPD
20	1.59375	199.50	209.53	PPD
20	0.796875	301.50	298.44	PPD
20	0.3984375	364.00	350.44	PPD
20	0.19921875	369.50	372.74	PPD
20	0.099609375	367.50	381.02	PPD
20	0.049804688	390.00	383.93	PPD
20	20	13.50	18.48	WHO
20	10	33.50	39.44	WHO
20	5	62.00	78.80	WHO
20	2.5	161.00	140.57	WHO
20	1.25	216.00	214.66	WHO
20	0.625	286.50	279.55	WHO
20	0.3125	294.50	322.47	WHO
20	0.15625	338.50	345.85	WHO
20	0.078125	381.50	357.26	WHO
20	0.0390625	361.00	362.52	WHO

IU = international units; MNT = microneutralization; PPD = Pharmaceutical Product Development;
WHO = World Health Organization.
