# Supplementary material for: Robust validation and performance comparison of immunogenicity assays assessing IgG and neutralizing antibodies to SARS-CoV-2
Source: PLoS One. 2022 Feb 7;17(2):e0262922. doi: 10.1371/journal.pone.0262922 (PMC8820625; doi:10.1371/journal.pone.0262922)
Supplement: S7 Table — Specificity analysis: (A) Competitors used (B) MSD ECL assay, and (C) MNT assay. Ab[C] = antibody concentrations; AU = arbitrary units; MNT = microneutralization; MSD ECL = multiplex electrochemiluminescence; N = nucleocapsid; PBS = phosphate-buffered saline; RBD = receptor-binding domain; S = spike; SARS-CoV-2 = severe acute respiratory syndrome coronavirus 2. (PDF) [file pone.0262922.s008.pdf]

S7 Table. Specificity analysis

(A) Competitors used

Assay	Competitor Type	Competitor	% Inhibition
MSD ECL SARS-CoV-2 S	Homologous	Spike S1/S2	94.6%
MSD ECL SARS-CoV-2 S	Heterologous	Nucleocapsid	2.3%
MSD ECL SARS-CoV-2 S	Known cross-reactive	RBD	51.3%
MSD ECL SARS-CoV-2 S	Irrelevant heterologous	HC OC43	11.6%
MSD ECL SARS-CoV-2 N	Heterologous	Spike S1/S2	-1.8%
MSD ECL SARS-CoV-2 N	Homologous	Nucleocapsid	98.5%
MSD ECL SARS-CoV-2 N	Heterologous	RBD	-1.1%
MSD ECL SARS-CoV-2 N	Irrelevant heterologous	HC OC43	11.5%
MSD ECL SARS-CoV-2 RBD	Known cross-reactive	Spike S1/S2	98.5%
MSD ECL SARS-CoV-2 RBD	Heterologous	Nucleocapsid	0.5%
MSD ECL SARS-CoV-2 RBD	Homologous	RBD	97.4%
MSD ECL SARS-CoV-2 RBD	Irrelevant heterologous	HC OC43	-18.2%
SARS-CoV-2 MNT	Homologous	Spike S1/S2	89.8%
SARS-CoV-2 MNT	Irrelevant heterologous	Influenza A	1.5%
SARS-CoV-2 MNT	Irrelevant heterologous	HC OC43	-36.4%

MNT = microneutralization; MSD ECL = multiplex electrochemiluminescence; N = nucleocapsid; RBD = receptor-binding domain; S = spike; SARS-CoV-2 = severe acute respiratory syndrome coronavirus 2.

S7 Table. Specificity analysis

(B) MSD ECL assay

Antigen	Competitor	% Inhibition
MSD ECL SARS-CoV-2 S	Homologous Competitor (SARS-CoV-2 S)	94.6%
MSD ECL SARS-CoV-2 S	Heterologous Competitor (SARS-CoV-2 N)	2.3%
MSD ECL SARS-CoV-2 S	Heterologous Competitor (SARS-CoV-2 RBD)	51.3%
MSD ECL SARS-CoV-2 S	Heterologous Competitor (Human Coronavirus OC43)	11.6%
MSD ECL SARS-CoV-2 N	Heterologous Competitor (SARS-CoV-2 S)	-1.8%
MSD ECL SARS-CoV-2 N	Homologous Competitor (SARS-CoV-2 N)	98.5%
MSD ECL SARS-CoV-2 N	Heterologous Competitor (SARS-CoV-2 RBD)	-1.1%
MSD ECL SARS-CoV-2 N	Heterologous Competitor (Human Coronavirus OC43)	11.5%
MSD ECL SARS-CoV-2 RBD	Heterologous Competitor (SARS-CoV-2 S)	98.5%
MSD ECL SARS-CoV-2 RBD	Heterologous Competitor (SARS-CoV-2 N)	0.5%
MSD ECL SARS-CoV-2 RBD	Homologous Competitor (SARS-CoV-2 RBD)	97.4%
MSD ECL SARS-CoV-2 RBD	Heterologous Competitor (Human Coronavirus OC43)	-18.2%

MSD ECL = multiplex electrochemiluminescence; N = nucleocapsid; RBD = receptor-binding domain; S = spike; SARS-CoV-2 = severe acute respiratory syndrome coronavirus 2.

(B) MSD ECL assay (continued)

			Pre-adsorbed with:													
		Run	Ab[C] (AU/ml)							Percent specificity						
Sample	Antigen	#	Water	Mock	SARS-CoV-2 S	SARS-CoV-2 N	SARS-CoV-2 RBD	OC43	OC43	Water	Mock	SARS-CoV-2 S	SARS-CoV-2 N	SARS-CoV-2 RBD	OC43	OC43
1	SARS-CoV-2 S	1	97301	100195	3699	99948	43370	98900	102146	0.0%	-3.0%	96.4%	-2.7%	55.6%	-1.6%	-5.0%
1	SARS-CoV-2 N	1	51306	50752	49558	2716	51626	39745	39534	0.0%	1.1%	3.4%	95.2%	-0.6%	22.7%	23.1%
1	SARS-CoV-2 RBD	1	45471	46546	1047	44854	1404	48106	46237	0.0%	-2.4%	98.1%	1.4%	97.3%	-5.8%	-1.7%
2	SARS-CoV-2 S	1	39845	41145	5044	41865	16996	39910	38864	0.0%	-3.3%	87.8%	-5.1%	57.7%	-0.2%	2.5%
2	SARS-CoV-2 N	1	>160000	>160000	>160000	5935	>160000	>160000	>160000	0.0%	-10.2%	-8.3%	97.3%	-6.5%	10.7%	11.1%
2	SARS-CoV-2 RBD	1	17131	17224	719	16530	819	17576	16713	0.0%	-0.5%	96.9%	3.5%	96.3%	-2.6%	2.5%
3	SARS-CoV-2 S	1	14852	15064	1849	14353	6951	13513	13314	0.0%	-1.4%	88.9%	3.4%	54.0%	9.2%	10.5%
3	SARS-CoV-2 N	1	44647	43368	46164	755	45748	35426	35975	0.0%	2.9%	-3.4%	98.9%	-2.5%	20.8%	19.5%
3	SARS-CoV-2 RBD	1	5496	5036	<190	5953	259	5701	5980	0.0%	8.7%	100.0%	-8.6%	98.7%	-3.9%	-9.1%
4	SARS-CoV-2 S	1	17701	17399	1339	17463	6432	17438	17483	0.0%	1.7%	93.7%	1.4%	64.5%	1.5%	1.2%
4	SARS-CoV-2 N	1	10075	9866	10525	<270	10609	9819	9649	0.0%	2.1%	-4.6%	100.0%	-5.4%	2.6%	4.3%
		1	9344	9929	442	9443	776	9964	9452	0.0%	-6.4%	97.2%	-1.1%	93.6%	-6.8%	-1.2%
		1	14495	15579	1266	15520	6949	14480	14360	0.0%	-7.6%	92.7%	-7.2%	52.9%	0.1%	0.9%
		1	>160000	>160000	>160000	2548	>160000	148081	148661	0.0%	2.5%	6.9%	98.7%	2.7%	17.7%	17.4%
5	SARS-CoV-2 RBD	1	5732	5165	260	5569	343	5342	6139	0.0%	10.2%	98.7%	2.9%	97.2%	7.0%	-7.3%
6	SARS-CoV-2 S	1	8827	7831	439	8552	4088	7820	8157	0.0%	11.6%	97.6%	3.2%	55.1%	11.7%	7.8%
6	SARS-CoV-2 N	1	26245	28858	27875	500	27385	24993	24696	0.0%	-10.1%	-6.3%	99.1%	-4.4%	4.8%	6.0%
6	SARS-CoV-2 RBD	1	3012	3435	<190	3113	<190	3349	3202	0.0%	-15.0%	100.0%	-3.6%	100.0%	-11.9%	-6.7%
7	SARS-CoV-2 S	1	915	941	<230	803	555	731	700	0.0%	-3.8%	100.0%	16.4%	52.6%	26.9%	31.4%
7	SARS-CoV-2 N	1	36472	35861	36477	893	35709	27923	27459	0.0%	1.7%	0.0%	98.3%	2.1%	23.6%	24.9%
7	SARS-CoV-2 RBD	1	222	196	<190	226	<190	272	286	0.0%	81.3%	100.0%	-12.5%	100.0%	-156.3%	-200.0%
8	SARS-CoV-2 S	1	2737	2662	<230	2785	1827	1927	1885	0.0%	3.0%	100.0%	-1.9%	36.3%	32.3%	34.0%
8	SARS-CoV-2 N	1	3106	3080	3301	<270	3282	3735	3685	0.0%	0.9%	-6.9%	100.0%	-6.2%	-22.2%	-20.4%
8	SARS-CoV-2 RBD	1	742	779	<190	755	<190	910	832	0.0%	-6.7%	100.0%	-2.4%	100.0%	-30.4%	-16.3%

1	SARS-CoV-2 S	2	105386	106331	4057	107625	44325	102605	101458	0.0%	-0.9%	96.4%	-2.1%	58.1%	2.6%	3.7%
1	SARS-CoV-2 N	2	53250	53706	52072	2694	50276	40453	39594	0.0%	-0.9%	2.2%	95.4%	5.6%	24.2%	25.8%
1	SARS-CoV-2 RBD	2	49227	48391	1220	50327	1640	46546	46796	0.0%	1.7%	97.9%	-2.2%	97.0%	5.5%	5.0%
2	SARS-CoV-2 S	2	45045	43206	5543	43996	18932	43248	42394	0.0%	4.1%	88.1%	2.3%	58.3%	4.0%	5.9%
2	SARS-CoV-2 N	2	>160000	>160000	>160000	5172	>160000	>160000	>160000	0.0%	-1.7%	-1.1%	97.8%	0.0%	17.2%	16.8%
2	SARS-CoV-2 RBD	2	17254	17180	985	17452	1067	18777	18462	0.0%	0.4%	95.3%	-1.2%	94.9%	-8.9%	-7.1%
3	SARS-CoV-2 S	2	15412	15766	1759	14568	7450	13979	14050	0.0%	-2.3%	89.9%	5.6%	52.4%	9.4%	9.0%
3	SARS-CoV-2 N	2	45845	46334	46592	680	47079	36191	36723	0.0%	-1.1%	-1.6%	99.1%	-2.7%	21.2%	20.0%
3	SARS-CoV-2 RBD	2	5902	5397	<190	6065	242	5664	5771	0.0%	8.8%	100.0%	-2.9%	99.1%	4.2%	2.3%
4	SARS-CoV-2 S	2	18142	18566	1272	18568	6666	17754	18506	0.0%	-2.4%	94.2%	-2.4%	64.1%	2.2%	-2.0%
4	SARS-CoV-2 N	2	10631	10296	10805	<270	10581	9797	9755	0.0%	3.2%	-1.7%	100.0%	0.5%	8.0%	8.5%
4	SARS-CoV-2 RBD	2	10124	10405	414	9888	726	9978	9784	0.0%	-2.8%	97.7%	2.4%	94.6%	1.5%	3.4%
5	SARS-CoV-2 S	2	17727	18125	1498	16800	7525	15970	16273	0.0%	-2.3%	92.8%	5.3%	58.3%	10.0%	8.3%
5	SARS-CoV-2 N	2	>160000	>160000	>160000	2062	>160000	>160000	159884	0.0%	-1.0%	4.7%	99.1%	5.7%	18.9%	20.1%
5	SARS-CoV-2 RBD	2	7165	6621	370	6620	539	6392	6202	0.0%	7.8%	97.4%	7.8%	95.0%	11.1%	13.8%
6	SARS-CoV-2 S	2	9487	9629	649	9591	4746	8727	8925	0.0%	-1.5%	95.5%	-1.1%	51.2%	8.2%	6.1%
6	SARS-CoV-2 N	2	29013	31046	30344	522	30359	26630	26465	0.0%	-7.1%	-4.6%	99.1%	-4.7%	8.3%	8.9%
6	SARS-CoV-2 RBD	2	3443	3870	275	3889	361	3738	3650	0.0%	-13.1%	97.4%	-13.7%	94.7%	-9.1%	-6.4%
7	SARS-CoV-2 S	2	1158	1114	<230	1113	780	908	879	0.0%	4.7%	100.0%	4.8%	40.7%	26.9%	30.1%
7	SARS-CoV-2 N	2	35542	37473	37392	772	35598	28853	29115	0.0%	-5.5%	-5.2%	98.6%	-0.2%	19.0%	18.2%
7	SARS-CoV-2 RBD	2	301	273	<190	265	<190	384	396	0.0%	25.2%	100.0%	32.4%	100.0%	-74.8%	-85.6%
8	SARS-CoV-2 S	2	3634	3431	252	3059	3302	2192	2235	0.0%	6.0%	99.4%	16.9%	9.8%	42.4%	41.1%
8	SARS-CoV-2 N	2	3393	3325	3454	<270	3449	3869	3933	0.0%	2.2%	-2.0%	100.0%	-1.8%	-15.2%	-17.3%
8	SARS-CoV-2 RBD	2	931	934	<190	885	<190	881	879	0.0%	-0.4%	100.0%	6.2%	100.0%	6.7%	7.0%

Ab[C] = antibody concentrations; AU = arbitrary units; MSD ECL = multiplex electrochemiluminescence; N = nucleocapsid; RBD = receptor-binding domain; S = spike; SARS-CoV-2 = severe acute respiratory syndrome coronavirus 2.

S7 Table. Specificity analysis

(C) MNT assay

Antigen	Competitor	% Inhibition
SARS-CoV-2 MNT	Mock 1 (Water)	6.9%
SARS-CoV-2 MNT	Mock 2 (PBS)	-0.6%
SARS-CoV-2 MNT	Mock 3 (PBS)	-1.0%
SARS-CoV-2 MNT	Homologous Competitor	89.8%
SARS-CoV-2 MNT	Heterologous Competitor (Influenza A)	1.5%
SARS-CoV-2 MNT	Heterologous Competitor (Human Coronavirus OC43)	-36.4%

MNT = microneutralization; PBS = phosphate-buffered saline; SARS-CoV-2 = severe acute respiratory syndrome coronavirus 2.

(C) MNT assay (continued)

Sample	Treatment	Titer	% Specificity
1	Neat Sample	301	-9.7%
1	Mock 1 (Water)	280	-0.5%
1	Mock 2 (PBS)	270	3.9%
1	Mock 3 (PBS)	288	-4.0%
1	Homologous Competitor	50	100.0%
1	Homologous Competitor	53	98.7%
1	Heterologous Competitor (Influenza A)	233	20.0%
1	Heterologous Competitor (Human Coronavirus OC43)	252	11.7%
2	Neat Sample	390	-14.6%
2	Mock 1 (Water)	334	4.3%
2	Mock 2 (PBS)	406	-20.0%
2	Mock 3 (PBS)	296	17.1%
2	Homologous Competitor	144	68.3%
2	Homologous Competitor	134	71.7%
2	Heterologous Competitor (Influenza A)	RS	NE
2	Heterologous Competitor (Human Coronavirus OC43)	392	-15.3%
3	Neat Sample	168	-5.3%
3	Mock 1 (Water)	143	17.0%
3	Mock 2 (PBS)	146	14.4%
3	Mock 3 (PBS)	180	-16.0%
3	Homologous Competitor	61	90.2%
3	Homologous Competitor	62	89.3%
3	Heterologous Competitor (Influenza A)	170	-7.0%
3	Heterologous Competitor (Human Coronavirus OC43)	247	-75.7%
4	Neat Sample	NP	NE
4	Mock 1 (Water)	NP	NE
4	Mock 2 (PBS)	NP	NE
4	Mock 3 (PBS)	210	0.0%
4	Homologous Competitor	<50	100.0%
4	Homologous Competitor	<50	100.0%
4	Heterologous Competitor (Influenza A)	221	-6.9%
4	Heterologous Competitor (Human Coronavirus OC43)	316	-66.3%

PBS = phosphate-buffered saline.
